# Predicted genetic burden and frequency of phenotype-associated variants in the horse

**DOI:** 10.1038/s41598-024-57872-8

**Published:** 2024-04-10

**Authors:** S. A. Durward-Akhurst, J. L. Marlowe, R. J. Schaefer, K. Springer, B. Grantham, W. K. Carey, R. R. Bellone, J. R. Mickelson, M. E. McCue

**Affiliations:** 1 Department of Veterinary Clinical Sciences, University of Minnesota, C339 VMC, 1353 Boyd Avenue, St. Paul, MN 55108 USA; 2 Department of Veterinary Population Medicine, University of Minnesota, 225 VMC, 1365 Gortner Avenue, St. Paul, MN 55108 USA; 3 Interval Bio LLC, 408 Stierline Road, Mountain View, CA 94043 USA; 4 Veterinary Genetics Laboratory, School of Veterinary Medicine, University of California-Davis, Davis, CA USA; 5 Population Health and Reproduction and Veterinary Genetics Laboratory, School of Veterinary Medicine, University of California, Davis, CA USA; 6 Department of Veterinary and Biomedical Sciences, University of Minnesota, 295F Animal Science Veterinary Medicine Building, 1988 Fitch Avenue, St. Paul, MN 55108 USA

**Keywords:** Biochemistry, Diseases, Genetics, Genomics, Medical genetics, Population genetics

## Abstract

Disease-causing variants have been identified for less than 20% of suspected equine genetic diseases. Whole genome sequencing (WGS) allows rapid identification of rare disease causal variants. However, interpreting the clinical variant consequence is confounded by the number of predicted deleterious variants that healthy individuals carry (predicted genetic burden). Estimation of the predicted genetic burden and baseline frequencies of known deleterious or phenotype associated variants within and across the major horse breeds have not been performed. We used WGS of 605 horses across 48 breeds to identify 32,818,945 variants, demonstrate a high predicted genetic burden (median 730 variants/horse, interquartile range: 613–829), show breed differences in predicted genetic burden across 12 target breeds, and estimate the high frequencies of some previously reported disease variants. This large-scale variant catalog for a major and highly athletic domestic animal species will enhance its ability to serve as a model for human phenotypes and improves our ability to discover the bases for important equine phenotypes.

## Introduction

The horse, unlike other domestic animal species that have predominantly been bred for food, fiber, or appearance, has been selected for athleticism and strength^1^. This makes the horse a useful model for many exercise-related human traits, including: endurance, racing distance^2^, speed^3^, power, and athleticism, musculoskeletal diseases, including osteoarthritis^4^, developmental orthopedic disease^5^, muscle diseases^6–8^, and metabolic diseases^9^. While selective breeding has developed breeds with desirable traits^1,10^, it has also decreased genetic diversity^11^, thereby increasing the risk of inbreeding depression (i.e., the accumulation of deleterious variants^12^ and increasing homozygosity^13^ leading to decreased average phenotypic performance). This has unfortunately resulted in numerous breeds with high incidences of deleterious Mendelian disease traits^10,14–28^. Studies focused on the identification of causal variants for equine diseases with human analogs will shed new light on both horse and human biology and pathophysiology and may demonstrate further utility of the horse as a non-traditional animal model, especially for athletic-related traits and disorders.

The utility of large-scale catalogs of genetic variation for the discovery of disease-causing variants^29–32^ and prioritization of variants in genomic regions of interest^33^ is now well established. These efforts in humans have demonstrated a higher than expected frequency of variants computationally predicted to have a detrimental impact on phenotype (i.e., the predicted genetic burden)^34,35^. Based on higher than expected frequencies in the general population, several previously reported disease-causing variants have been reclassified due to having insufficient evidence to support them causing disease without the contribution of additional variants in a more complex genetic architecture^36,37^. Despite extensive research on genetic disease in the horse, disease-causing variants have been identified for less than 20% of the currently recognized equine suspected genetic traits and diseases (https://omia.org/home/). Large scale catalogs of equine genetic variation from whole genome sequence (WGS) of 88–534 individual horses have been previously reported^38–40^. However, none of these catalogs specifically focus on the predicted genetic burden in the equine population.

Here we have expanded our equine WGS database from 534^38^ to 605 horses, representing 48 breeds (Supplementary Table 1). We demonstrate that the predicted genetic burden per horse is 1.4–2.6-fold higher than in humans, and show that, as in humans, several previously suspected disease- and trait-associated variants are present at much higher frequencies than expected based on the published estimates of disease prevalence. This is the first demonstration of the predicted genetic burden and elevated frequency of disease- and trait-associated variants in domestic animals.

## Results

### Equine genetic variation summary

**Figure 1:**
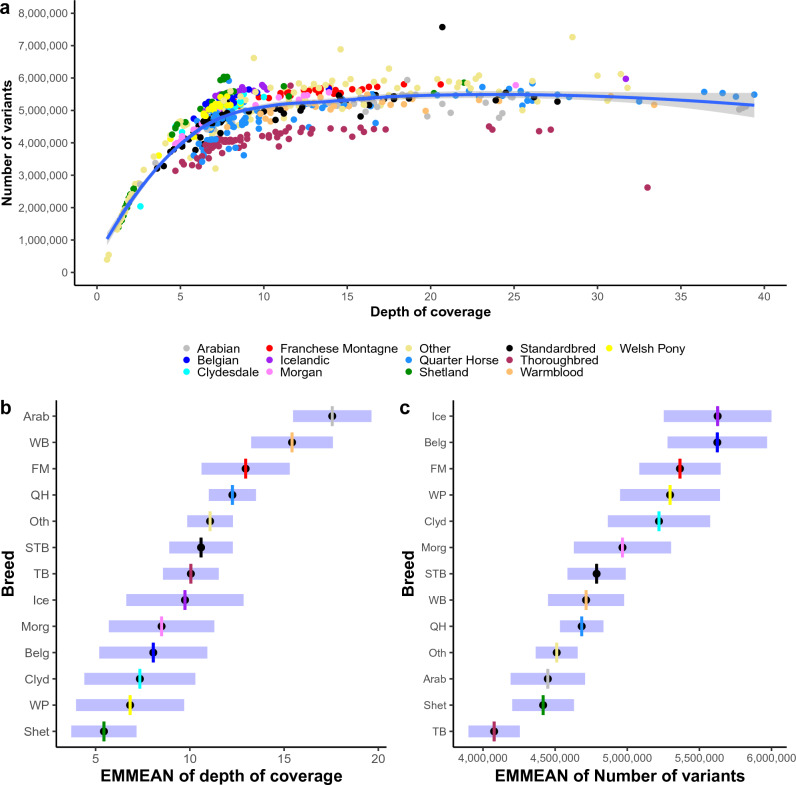
Relationship between the number of variants identified and the depth of coverage. (**a**) The correlation between the number of variants identified in the 605 horses and the WGS depth of coverage (DOC). The blue line represents the non-linear correlation between the number of variants identified and DOC, with grey shadowing representing the 95% confidence intervals around the mean. The breed EMMEAN is represented by a colored line. Breeds comprised: Arabian (Arab), Belgian (Belg), Clydesdale (Clyd), Franches-Montagnes (FM), Icelandic (Ice), Morgan (Morg), Other breeds (Oth), Quarter Horse (QH), Shetland (Shet), Standardbred (STB), Thoroughbred (TB), Warmblood (WB), and Welsh Pony (WP). (**b**) Estimated Marginal Mean (EMMEAN) for the linear regression between depth of coverage (DOC) and breed. c. EMMEAN for the linear regression between the number of variants by breed, accounting for DOC. For b and c the vertical colored lines represent the EMMEAN and the purple horizontal bands represent the 95% confidence limits around the EMMEAN.

All genomes were sequenced using Illumina sequencing-by-synthesis technology^38^. Sequence reads were aligned with the EquCab3.0 reference genome^41^, and single nucleotide polymorphisms (SNPs) and small insertions and deletions (≤ 20 base pairs in length, indels) called using a modified version of the GATK best practices pipeline^42^. In total, 32,818,945 variants (28,913,164 SNPs and 3,905,781 indels) with a mean depth of coverage (DOC) of 10.9 X (range: 0.6–39.4X) were identified. The average number of variants per genome was 4,687,726 variants (4,236,658 SNPs and 451,068 indels). On average, 9978 variants were private to that genome. The number of variants identified was significantly correlated with the depth of coverage (*p* < 0.0001, Fig. 1a). Therefore, estimated marginal means (EMMEANS) accounting for breed and DOC were calculated for the breed specific analyses. Of the 605 horses, 493 horses of twelve breeds met the criteria (> 15 individuals with a mean depth of coverage > 5X) for breed analyses, namely: Arabian, Belgian, Clydesdale, Franches Montagnes, Icelandic, Morgan, Quarter Horse, Shetland, Standardbred, Thoroughbred, Warmblood, and Welsh Pony (Supplementary Table 1). Breed analysis was performed on the 12 target breeds with WGS at a mean depth of coverage > 5X available from 17 of more individuals. The mean depth of coverage was significantly different between breeds (*p* < 0.0001) with the lowest depth of coverage in Shetlands (EMMEAN 5.44 X, 95% confidence interval 3.71–7.17) and highest in Arabians (EMMEAN 17.56 X, 95% confidence interval 15.47–19.64, Fig. 1b). There was a statistically significant difference in the number of variants identified for each breed (*p* < 0.0001) with the lowest number of variants in Thoroughbreds (EMMEAN 4,077,265, 95% confidence interval 3,899,415–4,255,116) and highest in Icelandic horses (EMMEAN 5,626,374, 95% confidence interval 5,253,019–5,999,729, Fig. 1c).

### Estimation of the predicted genetic burden in the horse

Across all 605 genomes, SnpEff identified 36,169 high impact variants, 216,987 moderate impact variants, and 418,334 low impact variants. Ensembl-VEP identified 25,834 high impact variants, 179,827 moderate impact variants, and 361,248 low impact variants. The remaining variants were considered “modifier” variants, which are mostly noncoding and considered difficult to predict the likely impact. 25,550 variants were identified as high impact by both annotators, 179,480 variants were identified as moderate impact by both annotators, and 358,514 variants were identified as low impact by both annotators (Table 1).

**Table 1:** Overlap between SnpEff and Ensembl-VEP predicted variant impact. The variants included in the predicted genetic burden analysis are highlighted in bold.

SnpEff Impact	High	**25,550**	**336**	392
	Moderate	**58**	179,480	2,000
	Low	176	4	358,514
		High	Moderate	Low
		Ensembl-VEP impact		

The number of predicted deleterious variants (predicted genetic burden) was calculated using the number of variants predicted to be high impact by SnpEff and Ensembl-VEP, or high impact by one effect predictor and moderate impact by the other. Across all 605 horses, the predicted genetic burden was 0.08% (i.e., 25,944 variants [18,975 SNPs and 6969 indels] called by both annotators). 25,550 variants were called high impact by both Ensembl-VEP and SnpEff, 336 variants were called high impact by SnpEff and moderate impact by Ensembl-VEP, and 58 variants were called moderate by SnpEff and high impact by Ensembl-VEP (Table 1). Ensembl VEP and SnpEff agreed on the variant type for 23,000 (88.7%) of the predicted genetic burden variants (Table 2). The median predicted genetic burden was 730 variants per horse (interquartile range: 613–829), including a median of 230 (interquartile range: 189–275) homozygous predicted genetic burden variants. The median variant frequency was 0.16% (interquartile range: 0.08%—0.33%), which was significantly lower (*p* < 0.0001, 95% confidence interval 1.75–1.92%) than the median frequency 2.00% (interquartile range: 0.25–10.30%) of the variants not included in the predicted genetic burden.

**Table 2:** Predicted variant type reported by SnpEff and Ensembl-VEP for each predicted genetic burden variant.

SnpEff	Ensembl-VEP	Count
Variant types with agreement between SnpEff and Ensembl-VEP		
Frameshift	Frameshift	15,999
Splice acceptor	Splice acceptor	1800
Splice donor	Splice donor	2066
Start lost	Start lost	277
Stop gained	Stop gained	2685
Stop lost	Stop lost	171
Transcript ablation	Transcript ablation	2
	Total	23,000
Variant types without agreement between SnpEff and Ensembl-VEP		
Exon loss	Frameshift	3
	Inframe deletion	1
	Splice acceptor	11
	Splice donor	25
Frameshift	Inframe deletion	13
	Inframe insertion	3
	Protein altering	2
	Splice acceptor	537
	Splice donor	765
	Start lost	10
	Stop gained	256
	Stop lost	5
Gene fusion	Frameshift	2
	Inframe deletion	1
	Splice acceptor	2
	Splice donor	1
Inframe deletion	Splice acceptor	10
	Splice donor	5
	Start lost	4
	Stop lost	1
Inframe insertion	Frameshift	1
Missense	Splice acceptor	2
	Splice donor	1
	Start lost	34
Splice acceptor	Frameshift	103
	Inframe deletion	3
	Inframe insertion	64
	Missense	14
	Protein altering	6
	Splice donor	404
	Stop gained	40
Splice donor	Frameshift	374
	Inframe insertion	157
	Missense	26
	Protein altering	6
	Stop gained	7
Start lost	Missense	15
Stop gained	Missense	20
	Splice acceptor	1
	Splice donor	1
Stop lost	Missense	5
	Splice acceptor	3
	Total	2944

The 25,944 predicted genetic burden variants were present in 9387 Ensembl gene IDs. Most Ensembl gene IDs (4211) contained only a single predicted genetic burden variant, with a median of 2 (interquartile range: 1–3) per gene. The median variant frequency was 0.15% (interquartile range: 0.08–0.33%). Of the 9387 Ensembl gene IDs, 675 had HGNC symbols. Most (311) contained a single predicted genetic burden variant, with a median of 2 (interquartile range: 1–3) per gene. The median variant frequency was 0.12% (interquartile range: 0.08–0.33%).

The 774 Ensembl gene IDs that contained > 5 predicted genetic burden variants and the 719 Ensembl gene IDs containing variants with a mean variant frequency > 5%, were functionally clustered using DAVID^43^. A single significant (*p* < 6.95 × 10^–5^) functional cluster for the genes containing > 5 predicted genetic burden variants (Supplementary Table 2) was identified. The significant cluster (enrichment score 6.05) term was ATP binding. Three corrected significant (p < 6.46 × 10^–5^) functional clusters for the genes with at least one predicted genetic burden variant that had a frequency > 5% (Supplementary Table 3) were identified. The first cluster (enrichment score 20.98) terms included olfactory receptor activity, olfactory receptor, olfactory transduction, olfaction, G-protein coupled receptor activity and rhodopsin-like, olfaction, and sensory transduction. The second cluster (enrichment score 6.92) terms included integral component of membrane. The third cluster (enrichment score 6.02) terms included odorant binding.

### The frequency of loss of function variants in the equine population

Loss of function (LOF) variants were defined as variants predicted to lead to a frameshift, splice site alteration, start or stop lost, and stop gained. Across all 605 horses, 18,990 of the predicted genetic burden variants were predicted to lead to LOF by both Ensembl-VEP and SnpEff. 3348 predicted genetic burden variants were predicted to be LOF by Ensembl VEP alone and 3036 were predicted to be LOF variants by SnpEff alone and were not considered LOF variants for this analysis. The median number of LOF variants was 417 per horse (interquartile range: 348–483), with a median 127 (interquartile range: 102–156) LOF variants present in a homozygous state. The median variant frequency was 0.16% (interquartile range: 0.08–0.33%).

The 18,990 LOF variants were present in 7682 Ensembl gene IDs. Most Ensembl gene IDs (3720) contained only a single LOF variant, with a median number of variants per Ensembl gene ID of 1 (interquartile range: 1–3). The median variant frequency in each Ensembl gene ID was 0.10% (interquartile range: 0.08–0.25%). Of the 7682 Ensembl gene IDs, 547 had HGNC symbols. Most HGNC genes (277) contained a single LOF variant, with a median of 1 (interquartile range: 1–3) per gene. The median variant frequency in each HGNC gene was 0.10% (interquartile range: 0.08–0.25%).

The 461 Ensembl gene IDs that contained > 5 LOF variants, and the 438 Ensembl genes containing variants with a mean frequency > 5%, were functionally clustered using DAVID^43^. A single significant (*p* < 1.14 × 10^–4^) functional cluster for the genes that contained > 5 LOF variants (supplementary table 4) was identified. The cluster (enrichment score 5.32) terms included ATP binding. Two significant (*p* < 1.28 × 10^–4^) functional clusters for the genes containing at least one LOF variant at > 5% frequency (Supplementary Table 5) were identified. The first cluster (enrichment score 10.8) terms included olfactory receptor, olfactory receptor activity, G-protein coupled receptor activity and rhodopsin-like, olfaction, sensory transduction, and transducer. The second cluster (enrichment score 4.03) terms included odorant binding.

### Estimation of the predicted genetic burden and number of loss of function variants in the 12 target breeds

**Figure 2:**
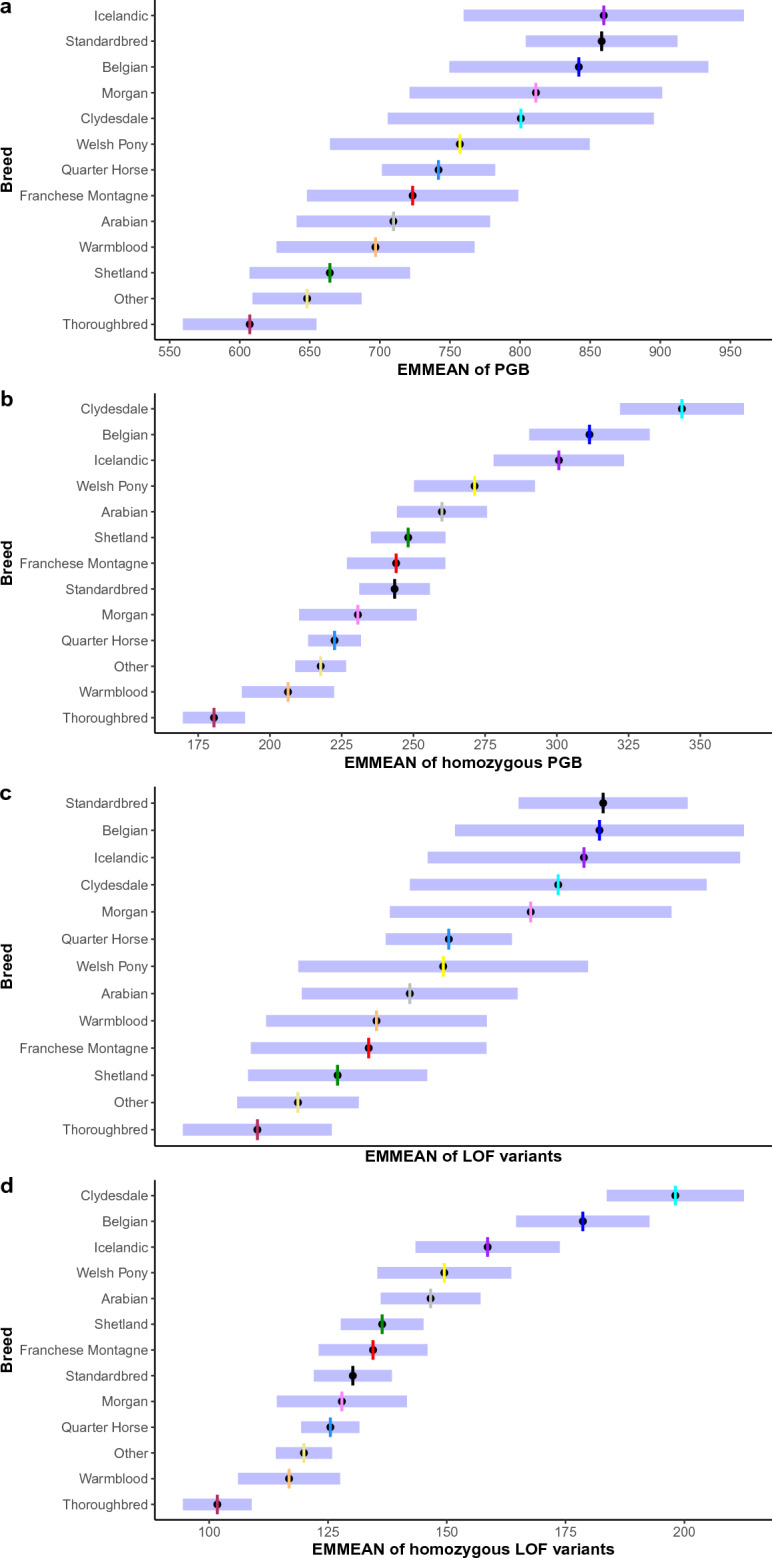
Estimated Marginal Means (EMMEANS) of the predicted genetic burden, the LOF burden, and the homozygous predicted genetic burden and LOF burden. EMMEANs (black circle) and 95% confidence interval (purple shaded line) for all predicted genetic burden variants (**a**), homozygous predicted genetic burden variants (**b**), all LOF variants (**c**), and homozygous LOF variants (**d**) in the 12 target breeds and other horses.

We investigated the predicted genetic burden in the 493 horses of the 12 breeds with whole genome sequence available from 17 or greater in individuals. The predicted genetic burden per individual was lowest in Thoroughbred horses (EMMEAN 607) and highest in Icelandic horses (EMMEAN 860) (Table 3, Fig. 2a). The number of homozygous predicted genetic burden variants per horse after accounting for depth of coverage also varied by breed, with the lowest in Thoroughbreds (EMMEAN 181) and the highest in Clydesdale horses (EMMEAN 344) (Table 3, Fig. 2b). The number of LOF variants per breed was lowest in Thoroughbreds (EMMEAN 355) and highest in Standardbred horses (EMMEAN 497) (Table 4, Fig. 2c). The number of homozygous LOF variants per individual horse after accounting for depth of coverage also varied by breed, with the lowest in Thoroughbred horses (EMMEAN 102) and highest in Clydesdale horses (EMMEAN 198) (Table 4, Fig. 2d).

**Table 3:** Estimated marginal means (EMMEANs) of the predicted genetic burden by breed. EMMEAN accounting for depth of coverage, standard error (SE), and 95% confidence intervals (CI) for the genetic burden for each breed with 17 or greater individuals for all variants and those only present in homozygous states.

Breed	EMMEAN	SE	Lower CI	Upper CI
Homozygous variants				
Thoroughbred	*181*	6	170	191
Warmblood	206	8	190	222
Other	218	5	209	227
Quarter Horse	223	5	213	232
Morgan	231	10	210	251
Standardbred	243	6	231	256
Franches-Montagnes	244	9	227	261
Shetland	248	7	235	261
Arabian	260	8	244	276
Welsh Pony	271	11	250	292
Icelandic	301	12	278	323
Belgian	311	11	290	332
Clydesdale	**344**	11	322	365
All predicted genetic burden variants				
Thoroughbred	*607*	24	560	655
Other	648	20	609	687
Shetland	664	29	607	722
Warmblood	697	36	626	767
Arabian	710	35	641	779
Franches-Montagnes	723	38	648	799
Quarter Horse	742	21	701	782
Welsh Pony	757	47	664	850
Clydesdale	801	48	706	895
Morgan	811	46	721	901
Belgian	842	47	750	934
Standardbred	858	28	804	912
Icelandic	**860**	51	760	960

Maximum EMMEAN (bold) and minimum EMMEAN (italic).

**Table 4:** Estimated marginal means (EMMEANs) of the LOF predicted genetic burden by breed. EMMEAN accounting for depth of coverage, standard error (SE), and 95% confidence intervals (CI) for the LOF predicted genetic burden for each breed with 17 or greater individuals for all variants and those only present in homozygous states.

Breed	EMMEAN	SE	Lower CI	Upper CI
Homozygous LOF variants				
Thoroughbred	*102*	4	94	109
Warmblood	117	5	106	128
Other	120	3	114	126
Quarter Horse	125	3	119	132
Morgan	128	7	114	142
Standardbred	130	4	122	138
Franches-Montagnes	134	6	123	146
Shetland	136	4	128	145
Arabian	147	5	136	157
Welsh Pony	149	7	135	164
Icelandic	159	8	143	174
Belgian	179	7	165	193
Clydesdale	**198**	7	184	213
All LOF variants				
Thoroughbred	*355*	16	324	385
Other	372	13	347	397
Shetland	388	19	351	425
Franches-Montagnes	401	25	352	449
Warmblood	404	23	358	449
Arabian	417	23	373	462
Welsh Pony	431	30	372	491
Quarter Horse	434	13	408	459
Morgan	467	29	409	525
Clydesdale	478	31	418	539
Icelandic	489	33	425	553
Belgian	495	30	436	555
Standardbred	**497**	18	462	532

Maximum EMMEAN (bold) and minimum EMMEAN (italic).

**Figure 3:**
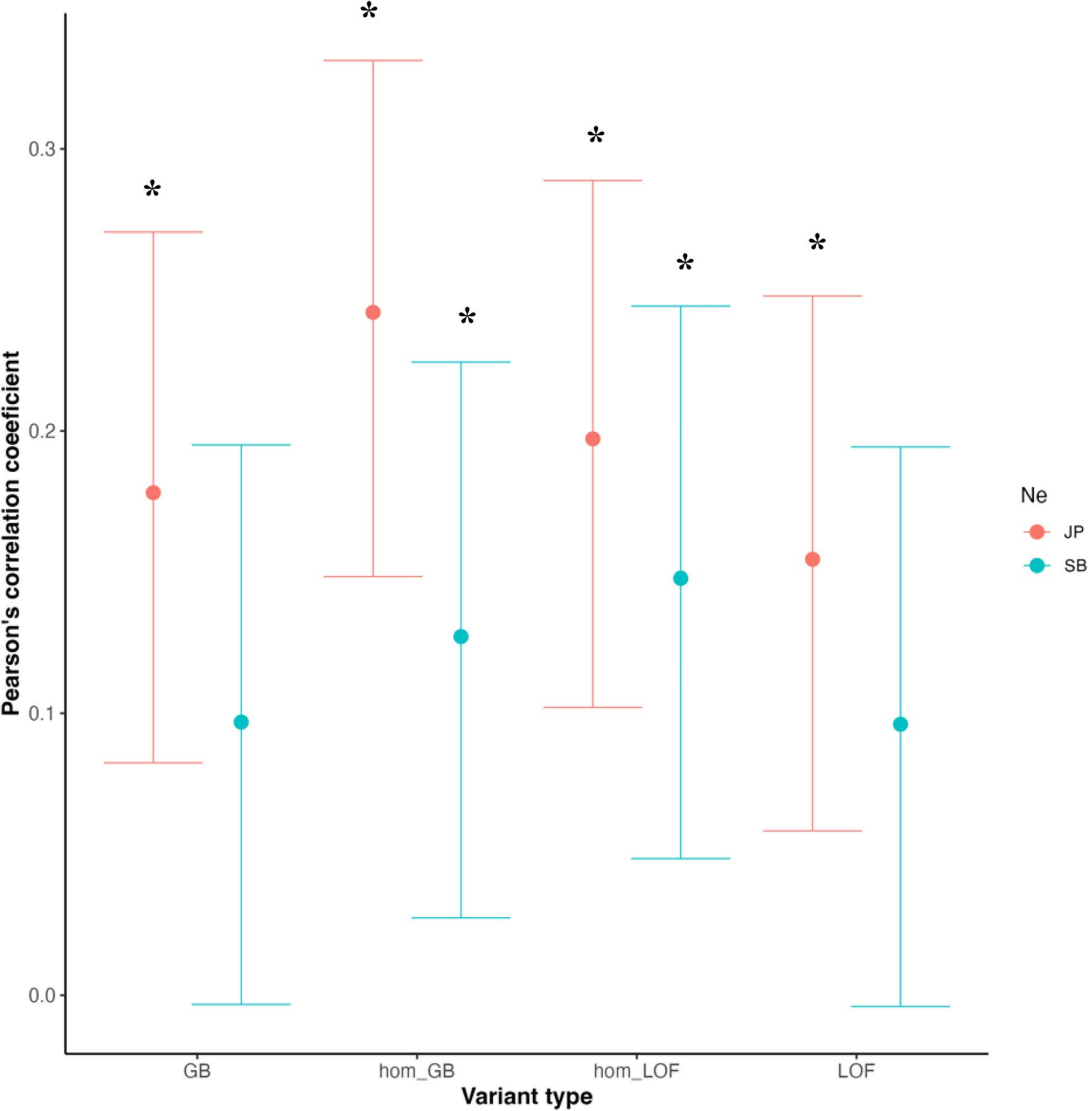
Pearson’s correlation coefficient estimates between the predicted genetic burden and LOF variants and estimates of breed Nes. The round points represent the Pearson’s correlation coefficient estimate with the 95% confidence interval represented by the error bars. Orange represents Ne estimates based on 54 K array data (JP) and teal represents Ne estimates based on 2 million array data (SB). The variant types are the predicted genetic burden (GB), homozygous predicted genetic burden (hom_GB), homozygous LOF (hom_LOF), and loss of function (LOF) variants. *represents significant correlation between the variant type and the estimated Ne.

We explored if the predicted genetic burden and number of LOF variants were correlated with two estimates of the effective population sizes (Ne) of different horse breeds^11,44^. The estimated Ne based on 54 K SNP array data breeds overlapped with 10 of the 12 breeds included in our breed analyses: Arabian, Belgian, Clydesdale, Franches Montagnes, Icelandic, Morgan, Quarter Horse, Shetland, Standardbred, and Thoroughbred^11^. The predicted genetic burden (*p* = 0.0002, Pearson’s correlation = 0.18, 95% confidence interval = 0.08–0.26), homozygous predicted genetic burden (*p* < 0.0001, Pearson’s correlation = 0.24, 95% confidence interval = 0.14–0.32), number of LOF variants (*p* = 0.002, Pearson’s correlation = 0.15, 95% confidence interval = 0.05–0.25), and number of homozygous LOF variants (*p* < 0.0001, Pearson’s correlation = 0.19, 95% confidence interval = 0.10–0.29) were significantly correlated with the estimated breed Nes based on 54 K SNP array data^11^ (Fig. 3).

The estimated Ne based on two million SNP array data breeds overlapped with 10 of the 12 breeds included in our breed analyses: Arabian, Belgian, Franches Montagnes, Icelandic, Morgan, Quarter Horse, Standardbred, Thoroughbred, and Welsh Pony^44^. The homozygous predicted genetic burden (*p* = 0.01, Pearson’s correlation = 0.13, 95% confidence interval = 0.03–0.22) and the number of homozygous LOF variants (*p* = 0.004, Pearson’s correlation = 0.15, 95% confidence interval = 0.05–0.24) were significantly correlated with the estimated breed Nes based on 2 million SNP array data^44^. The predicted genetic burden (*p* = 0.06, Pearson’s correlation = 0.10, 95% confidence interval = − 0.003–0.20) and the number of LOF variants (*p* = 0.06, Pearson’s correlation = 0.10, 95% confidence interval = − 0.004–0.19) were not significantly correlated with the 2 million array estimates of breed Ne^44^ (Fig. 3).

### Presence of previously reported causal and associated variants

**Figure 4:**
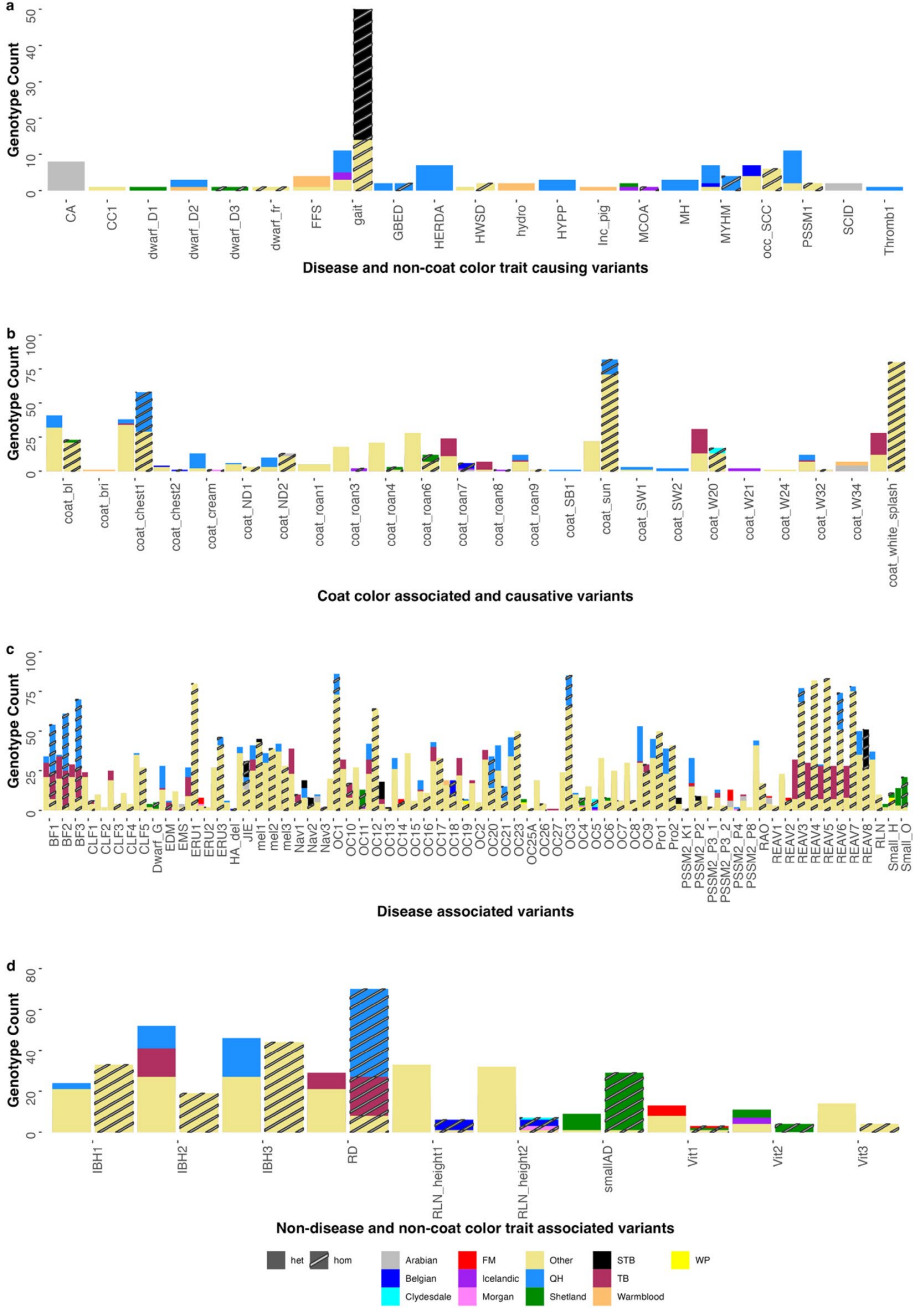
Known variants identified in the 605-horse population. Genotype count of and whether the variants are present in heterozygous (regular shading) or homozygous (diagonal striped shading) states for known disease and non-coat color trait causing variants (**a**), coat color associated and causative variants (**b**), disease associated variants (**c**), and non-disease and non-coat color trait associated variants (**d**) for each of the 12 target breeds and the other breed group. The phenotype abbreviations are detailed in Supplementary table 6.

The Online Mendelian Inheritance in Animals catalogue (https://omia.org/home/) was queried to identify variants that have previously been reported as causal or associated with an equine disease, coat color, or other trait. Reported locations of causal and associated variants for equine phenotypes were extracted from the OMIA catalogue and using a publicly available patent^45^ in January 2023 (Table 5, Supplementary Table 6). There were a reported 36 disease and performance trait causing variants, 62 coat color trait causing or associated variants, 73 disease associated variants, and 10 non-disease and non-coat color trait associated variants (Table 5). In the 605 horses, we identified between 41 and 100% of these variants (Table 5, Fig. 4a–d, Supplementary Table 6). The median variant frequency for all known variants in this cohort was 7.05% (interquartile range: 1.75–31.45%).

**Table 5:** Classification of OMIA variants by type. Number of: causative variants for disease and non-coat color traits, associated and causative variants for coat color, associated variants for disease, and associated variants for non-disease and non-coat color traits present in this cohort, and the median and range of the variant frequency (VF).

OMIA variant type	Number of OMIA variants	Number of OMIA variants in this cohort	Median variant frequency (%)	VF interquartile range (%)
Disease and non-coat color trait causing	36	21	0.33	0.16–0.58
Coat color causing and associated	62	24	2.60	0.64–10.60
Disease associated	73	67	16.40	5.80–43.80
Non-disease and non-coat color trait associated	10	10	13.15	5.58–31.65

## Discussion

Through large-scale whole genome sequencing we have quantified the predicted genetic burden and frequency of LOF variants in 605 horses and across 12 target breeds. Previously reported phenotype-causing and -associated variants were present in this cohort, with some variants occurring at higher than expected frequencies. Certain pathways were enriched for common variants (frequency > 5%) and multiple variants (> 5), including cell function, immunologic, and olfactory receptor pathways. Variant and predicted genetic burden information and details of the frequency of previously reported variants will greatly facilitate phenotype-causing variant identification for equine genetic traits.

The estimated median predicted genetic burden and LOF burden in the horse is higher (730 and 417 variants per horse, respectively) than the reported human mean predicted genetic burden per person of 281–515^35^ and mean LOF burden per person of 250–300^46^. This is consistent with the extreme historical population bottlenecks leading to smaller effective population sizes^11,12^, selection for particular traits^10^, and possible errors due to a poorer quality reference genome^41,47^ in the horse as compared with humans. Differences in predicted genetic burden between breeds are likely due to differences in their selective breeding histories and relatedness to the Thoroughbred, the source for the equine reference genome. The homozygous genetic and LOF burdens in the 12 target breeds were weakly but significantly correlated with estimates of breed Ne based on 54 K and 2 million SNP array data^11,44^. The predicted genetic burden and LOF burden were significantly correlated with estimates of breed Ne based on the 54 K array^11^, but not with estimates of breed Ne based on the two million SNP array^44^. This may be related to the different breeds investigated in the two Ne papers. The 54 K array paper^11^ included 10 out of 12 of the target breeds explored here, with estimates of Ne for Warmbloods and Welsh Ponies not performed. The two million SNP array paper^44^ also included 10 out of 12 of the target breeds explored here, with estimates of Ne size for Clydesdales and Warmbloods not performed. The correlations with the Ne are in the opposite direction to expected. We would expect smaller Ne sizes to have a higher predicted genetic burden because selection pressure to remove deleterious can be less effective in small populations^12^. This may be related to the effective population sizes not being small enough to suffer the negative effects on selection pressure. This is an issue that warrants further investigation.

The lower frequency of predicted genetic burden variants compared to variants not included as predicted genetic burden variants is consistent with reports in humans and other species, where likely damaging variants are less common than likely benign variants due to purifying selection^34,48^. The median frequency of predicted genetic burden variants in the 605 horses (0.16%) is consistent with reports that most LOF variants present in humans have a frequency < 5% in the general population^34^. There are some LOF variants with frequencies over 50% in humans^34^, and we found a number of predicted genetic burden and LOF variants with high frequencies in the horse as well. The mean and median predicted genetic burden variant frequencies in the 605 horses were 1.85% and 0.16% respectively, which fits with the skewed frequency distribution that we would expect for predicted genetic burden variants, with rare variants representing more of the predicted genetic burden than common variants.

The predicted genetic burden variants were present in a large number (9387 Ensembl IDs, with 675 having HGNC symbols) of genes. Most genes contained low numbers of predicted genetic burden variants, with the majority being present at a frequency < 2%. Functional clustering of genes with more than five predicted genetic burden or LOF variants identified a pathway involving ATP binding. This differs from human studies of LOF variants, where olfactory pathways were significantly enriched^34^. This may be related to mapping errors, as genes found to contain three or more LOF variants in humans were more likely to be caused by mapping errors^34^. Further investigation into the reason certain genes contain more than five predicted genetic burden variants is required. In comparison, functional clustering of genes carrying predicted genetic burden variants with a frequency greater than 5% identified clusters related to olfaction which aligns more closely with human LOF variant gene clustering^34^. Almost 75% of the equine predicted genetic burden variants were predicted to be LOF variants. The clustering of genes with LOF variant frequencies over 5% largely mirrored the predicted genetic burden variant gene clustering.

The low frequency of predicted genetic burden and LOF variants in the Thoroughbred was not expected, as it is well established that the Ne in the Thoroughbred is small^11,49^. However, the reference genome of the horse is derived from a Thoroughbred, so the true breed-specific predicted genetic burden may not be observed because Thoroughbred specific predicted genetic burden variants are considered reference. This illustrates an important consideration when investigating the predicted genetic burden and disease-causing variants for domestic animal species. Domestic animal reference genomes are usually based on either a single individual or only a few individuals from the same breed. At the same time, it is possible that individuals from breeds that are genetically far removed from the reference have an inflated predicted genetic burden due to their breed-specific variants not being present in the reference genome. This may be the reason that the Icelandic horses in the 605 horses have a higher predicted genetic burden, despite being one of the most outbred breeds with one of the highest reported Nes^11^. At this time, there is no solution for this issue in the horse. The Sardinian human genome project has attempted to account for this by creating a population specific reference based on the major allele of that population rather than the reference allele^50^. Creation of an equine pangenome is underway and will help resolve this issue. We only had WGS of 17 Icelandic horses in the 605 horses and it is possible that ascertainment bias also contributed to Icelandic horses having the highest genetic burden in this study.

Our study was blinded to phenotype so we cannot comment on the frequencies of any phenotype in these 605 horses. However, to ensure that we did not inadvertently select horses with a particular disease, no more than 10 horses are from any single suspected Mendelian disease phenotype and no more than 40 horses come from a single study of a complex trait were included. This, combined with our goal of having a minimum of 17 horses from the 12 target breeds that represent major breed groupings of genetic diversity, led to our conclusion that this catalog of genetic variation can be used to investigate the frequency of putative phenotype-causing variants in this cohort.

Variants present at high frequencies in a population are highly unlikely to be deleterious due to purifying selection. However, with intense selective breeding for certain phenotypes, higher than expected frequencies of a variant can occur in a particular breed or subtype of the breed under selection for that trait. For example, the *GBE1* variant responsible for the autosomal recessive trait Glycogen Branching Enzyme deficiency (GBED) has a much higher than expected frequency (~ 13%) in Western pleasure horses^51^, a subtype of the American Quarter Horse breed, than would be expected based on disease frequency in the Quarter Horse breed as a whole^8^. Whether the higher-than-expected variant frequency in the Western Pleasure horse subpopulation is due to subpopulation-specific genetic drift, selection or hitch-hiking, or popular sire effect is unknown. Interestingly, in the 605 horses of 48 different breeds, the variant is only found in Quarter Horses, and at a frequency of 2.8%, which is slightly lower than reports in other studies (5–8.3%)^19,51, 52^, This may reflect the subpopulation type of Quarter Horses that are present in the study cohort, as the variant frequency of GBED varies markedly based on the discipline that the Quarter Horse is bred for^51^, or non-random sampling resulting in bias in the study cohort. Conversely, this could also reflect the success of breeding programs to reduce the frequency of GBED since the variant was first reported almost 20 years ago^8^. Interestingly, there were two homozygous variant adult Quarter Horses in this cohort, one with 4X coverage at that site (4/4 reads) and the second with 6X coverage at that site (6/6 reads). Both horses were adults, one was 11 and the other was > 20 years at the time of collection. Follow up genotyping of the 11 year old established that the horse was heterozygous for the variant rather than homozygous. Follow up genotyping of the older horse was not possible due to DNA degradation, so it is unclear if this horse was truly homozygous for the GBED variant or if this was a genotype-by-sequencing error. Since GBED is a fatal neonatal glycogen storage disease and to-date adult horses homozygous for this variant have not been reported it seems most likely that the homozygous genotype represents sequencing errors due to lower coverage at that site^8,19^.

For myosin heavy chain myopathy (MYHM), which follows a dominant inheritance pattern with incomplete penetrance, the only breed with homozygotes identified for the variant was the Quarter Horse. The Quarter Horse MAF of 7.1% is higher than a previous report of 146 Quarter Horses (MAF 3.4%)^27^. The variant has only been reported in Quarter Horses and related breeds^27,52^, however, here we also identified a single copy of the variant in two Belgian horses, a Welsh Pony, and a Tennessee Walking Horse. Manual visualization of these variants showed them to be present in 2/7 reads for two horses, 2/6 and 2/12 reads. Three of these horses (two Belgians and the Welsh Pony) were sampled by our lab group and the breed confirmed at the time of sample collection. The Tennessee Walking Horse breed was reported by the owner. Follow up genotyping of the two Belgians and the Welsh Pony revealed that the Welsh Pony and one of the Belgians were homozygous reference, this is likely due to alignment errors and that these reads are mapped to one or more psudeogenes. The other Belgian was confirmed to be heterozygous for the MYHM variant using follow up genotyping. This is the first non-Quarter Horse related breed to be found to carry this variant.

Most of the other established disease-causing variants were present at frequencies $\le$ 5% consistent with detrimental Mendelian disease, although not necessarily at the previously reported variant frequencies (Fig. 4a, Supplementary Table 6). The frequency of the cerebellar abiotrophy variant^53^ in Arabians (10.5%) is higher than we would expect for a Mendelian disease. The eight Arabians that carried the variant were heterozygous with allele ratios of approximately 50% for the reference and nonreference allele based on read depth, which is consistent with the recessive inheritance pattern. A single adult registered Quarter Horse was called as heterozygous for this variant, based on the variant being present in 2/12 reads based on manual inspection. Follow up genotyping determined that this horse was homozygous for the reference allele, illustrating the importance of confirming genotypes when only a few reads support the unexpected call. The dwarf 2 (D2) variant^54^ was identified in three Quarter Horses and a Warmblood, which is unexpected as the variant was reported as being associated with a dwarfism phenotype in Miniature Horses. The variant was present in 2/3, 6/9, 18/24 and 4/11 reads. One of the Quarter Horses was registered, the other two were owner reported. The Warmblood (a Dutch Warmblood) is a publicly available sequence (ERR1527967). It is unknown if this variant is impacting the size of these individuals.

Gaitedness is a highly breed specific trait and the only breeds where the gait variant^55^ was homozygous only in known gaited breeds: French Trotters, Icelandic horses, Native Mongolian Chakouyi Horses, Standardbreds, and Tennessee Walking Horses^55,56^. We found a lower variant frequency in Morgan horses (4.5%) than previously reported (14%)^57^. Gait variant frequency was higher in Quarter Horses (5.2%) than previously reported (2.4%)^57^ (Fig. 4a, Supplementary Table 6).

The breed specific frequencies of the coat color variants varied from 0.5 to 100% (Fig. 4b, Supplementary Table 6). While OMIA describes the coat color variants as non-disease causing, some of the dominant white and splashed-white variants investigated are thought to be embryonic lethal and/or cause deafness, therefore these were included in the disease-causing variant category. A variant downstream from *MITF* g.20,147,039C > T (EquCab2.0)*,* was originally reported as associated with reduced forelimb white markings in Menorca Purebred horses and increased white facial markings in Spanish Purebred horses (coat_white_splash, Fig. 4b)^58^. In the 605 horses, we identified a C > A variant at the remapped location in EquCab3.0 (g.21,608,936) and when searching Ensembl, only the C > A variant is present. This variant is the major allele or present in greater than 50% of horses in all 12 target breeds and the other breed group in these 605 horses (MAF 50–100%, Supplementary Table 6). Interestingly, the C > A variant was homozygous in all 17 Icelandic horses, a breed in which white markings are accepted. It is unclear if this intronic variant causes white face and leg markings, or if it is only tagging another variant in the *MITF* transcription factor that is known to be a major regulator of pigmentation.

Several other coat color variants had distributions that are not necessarily consistent with the phenotypes reported in the initial publications. The brindle 1 (BR1) variant in *MBTPS2* (g.17,286,855T > C, coordinates from OMIA and manual remapping using the NCBI remapper tool)^59^ was reported in Quarter Horses with irregular vertical stripes that were seen in their coat over the neck, back, hindquarters, and upper legs. In this cohort, we identified a single British Warmblood horse that was heterozygous for the variant.

The coat color, cream dilution sunshine variant (g.31,705,726G > A, EquCab3, coordinates from OMIA and manual remapping using the NCBI remapper tool) in *SLC45A2*, was reported to produce a phenotype similar to the pearl dilution g.31,709,690G > A variant^60^. No horses were homozygous for the sunshine variant in the ~ 130 horses genotyped in the original study. We identified 146 heterozygotes and 390 homozygotes for the sunshine variant in the 605 horses. The A (non-reference) allele was the major allele in all 12 breed groups and the other breed group, with a breed frequency > 0.86 in Arabians, Belgians, Morgans, Thoroughbreds, and Quarter Horses suggesting more work is needed to determine if this is causal for reduction in pigmentation. The g.79,548,220C > T (EquCab3.0, coordinates from OMIA and manual remapping using the NCBI remapper tool) variant in *KIT,* named W20 has been reported in multiple breeds and is thought to impact the amount of white patterning was originally reported to increase the size of facial white markings^61,62^. This variant was the major allele in Clydesdale horses (frequency: 0.95) and was common (frequency $\ge$ 0.10) in Warmbloods, Belgians, Shetlands, Standardbreds, Quarter Horses, Welsh ponies, Thoroughbreds, Franches Montagnes horses and the ‘other’ breed group. Although we don’t have color phenotype data on these horses, due to the common nature of this variant across multiple breeds, further investigation of the phenotype caused by this variant may be warranted. The g.79,538,738C > T variant in *KIT* (W31) was reported as being associated with white spotting in ‘stock type’ horses^63^. We found the variant in Quarter Horses, Standardbreds, Icelandic horses, Belgian horses, one Coldblood, one Tennessee Walking Horse, Mongolian horses, and one Yakutian horse. The g.79,566,881T > C variant reported as W34 in *KIT* as being associated with increased white patterning in a Paint/Quarter Horse family was identified in Arabians (AF = 0.05), Morgans (AF = 0.05), Warmbloods (AF = 0.10), and Standardbreds (AF = 0.02) in this study^64^. Although we don’t have color phenotype data on these horses, given the wide distribution and/or frequency of W20, W31, and W32 across multiple breeds, further investigation of the phenotypes caused by these variants is warranted.

The frequency of the other coat variants and breed distribution were largely similar to previous reports^62,65^. Interestingly, the frequency of the agouti variant (g.26,067,462CAGCAGAAAAGA > C, EquCab3.0) in *ASIP* in Belgian horses was 0.58, with 7 horses homozygous for the deletion. All but one of these horses was homozygous for the chestnut variant in *MC1R* (g.36,979,560C > T) and are therefore likely chestnut. A *KIT* variant g.79,542,439A > G reported to be associated with the roan phenotype in Noriker horses was identified in three Welsh ponies, one Connemara, and four Yakut horses^66^. No homozygotes were present for this variant. At least one horse was homozygous for several of the other roan associated variants in *KIT* (roan 3: g.79,545,073C > G, roan 4: g.79,544,372 T > A, roan 6: g.79,540,110 T > C, roan 7: g.79,540,020G > A, roan 8: g.79,539,989 T > C, and roan 9: g.79,538,738C > T)^67^ suggesting that if these are causal, that they are not lethal when homozygous as previously suspected.

The breed specific frequency of the disease-associated variants ranged from 0.7% to 100% (Fig. 4c, Supplementary Table 6). Many of these diseases are complex traits and it is likely that 100s to 1000s of variants contribute to the development of the phenotype and the variants are likely of small effect size. However, it is unlikely that variants contributing to breed-specific diseases are the major allele in multiple breeds. The bone fracture 1–3 risk variants in *MSTN* identified in Thoroughbreds^68^ are present in this cohort in all breed groups and are the major allele in Thoroughbreds (frequency: 51.3%, 55.3%, and 54.6% for variant 1, 2, and 3 respectively). This suggests that if these variants are contributing to bone fracture, they likely have small effect sizes.

Congenital Liver Fibrosis (CLF) is a fatal hepatic disease originally reported in Franches Montagnes horses^69^, and more recently reported in Spanish horses^70^ as a Mendelian disease. However, the five reported variants (Fig. 4c, Supplementary Table 6) are all present in over nine different breeds and other breeds with a frequency in some breeds > 89%. 369/605 horses contained at least one out of five of the reported variants and five horses (one Quarter Horse and four Warmbloods) carried all five variants. Additionally, there are multiple breeds other than Franches Montagnes and Spanish horses that have homozygotes for at least one of the CLF variants. Thus, these associated variants are unlikely to cause CLF. Juvenile Idiopathic Epilepsy is a disease of Arabian horses, however, the reported variant^71^ is identified in both the heterozygous and homozygous states in all 12 breeds as well as the other horse breeds in this population. This variant is the major allele in Standardbreds and Arabians. Similar to the CLF variants, it is unlikely that this variant is contributing to juvenile idiopathic epilepsy following a simple Mendelian inheritance pattern. This is consistent with recent reports that demonstrated a lack of association between this variant and juvenile idiopathic epilepsy^72,73^.

Several variants are currently being commercially marketed for the diagnosis of polysaccharide storage myopathy type 2. All six of these variants (K1 in *COL6A3*, P2 in *MYOT*, P3 1 and 2 in *FLNC*, P4 in *MYOZ3*, and P8 in *PYROXD1*) were present in multiple breeds. Although the horses in this cohort were not phenotyped for muscle disease, the presence of these variants in multiple breeds with frequencies in several breeds > 0.05 supports other studies that suggest further investigation into the functional consequence of these variants as they are unlikely to be contributing to polysaccharide storage myopathy type 2 in a simple Mendelian inheritance pattern^74,75^.

Misclassified variants have a major impact on the field of medical genetics, as false positive variants can lead to mistrust of genetic testing from both clients and clinicians^76^. Additionally, in domestic animal species, an animal that tests positive for a disease-reported variant may not be bred due to the risk of passing on the reported variant or may be euthanized due to lack of effective treatment options. With the higher predicted genetic burden in horses than in humans, horses are at even higher risk of variant misclassification due to the relatively high number of predicted genetic burden variants found in otherwise healthy horses. Incomplete validation of variants can easily lead to compelling and plausible stories about their putative mechanisms^77^. Given that the false discovery rate of a variant identified in a human patient and absent from 50 unrelated controls is still 15%^78^, the likely false discovery rate in horses is even higher due to the increased predicted genetic burden in horses compared to humans. Therefore, extensive consideration and validation of putative disease-causing variants, including the collection of genetic, informatic, and experimental data^79,80^, is warranted before a genetic test is developed and marketed.

There are now numerous examples of horse breeds with high incidences of deleterious Mendelian traits (e.g., Fell pony syndrome, junctional epidermolysis bullosa, hyperkalemic periodic paralysis, glycogen branching enzyme deficiency, polysaccharide storage myopathy, hereditary equine regional dermal asthenia, malignant hyperthermia, severe combined immune deficiency, lavender foal syndrome). When accompanied by a comprehensive catalog of common and/or neutral variation from normal healthy individuals within a population, whole genome sequence from one to several patients with a simple/monogenic disease can often identify the disease-causing mutations, which is an attractive route to finding the genetic mutations for rare likely mendelian disease in the horse^29,30, 78–80^. In addition to the benefits of discovering disease-causing variants to horses, owners and veterinarians, the push to recognize naturally occurring models of human diseases to accelerate translational medicine in humans has made the identification of disease-causing variants in domestic and large animal species a high research priority. The importance of accurately identifying the true causative variants should not be underestimated. With the publication of several methods to determine if a gene is or is not tolerant to a damaging variant^79,80^, disease-causing variant identification has been further facilitated. As we work towards a one medicine approach, knowledge of genes that are tolerant or intolerant to LOF and other damaging variants across humans and domestic animal species has the power to improve our ability to correctly identify disease-causing variants.

Interpretation of variants on a population-wide scale is largely based on computational predictions. In the human literature this has been shown to give a good base for further investigation into the predicted genetic burden and LOF variants in a population. However, false positives can and do arise, as shown through experimental validation of the initial phase of the human 1000 genomes project, which determined that 56.5% of variants predicted to be LOF variants were false positives^34^. We utilized a modified version of the well-established GATK best practices^42^, including variant quality score recalibration (VQSR) in an attempt to minimize our false discovery rate.

A limitation to the breed specific analyses is the small within-breed sample size. We selected breeds with seventeen or more horses available to estimate breed specific predicted genetic burden, frequency of LOF variants, and variant frequencies. As more WGS becomes available, it will be important to continue to update the breed specific analyses with an improved estimate of breed values. Due to sample size constraints, we could not estimate variant frequencies less than 3% in the Icelandic horse (n = 17). In contrast, in the Quarter Horse (n = 104) we could estimate variant frequencies as low as 0.5%. It is important to note that of the disease-causing variants that we identified many of the variants were present in the expected breeds and at similar variant frequencies to previously published reports.

To conclude, we show that the predicted genetic burden and the frequency of LOF variants in horses is higher than in humans and demonstrate the power of large-scale genome sequencing for prioritizing disease-causing variants in domestic animal species. The resulting catalog of genetic variation can now be used for prioritizing variants for suspected genetic traits in the horse. Additionally, genes containing multiple predicted genetic burden and LOF variants should be examined with caution when prioritizing disease-causing variants in horses, as they may be tolerant to damaging variants or be due to errors in variant calling, annotation, or genotype estimation reference genome. Given the horse’s potential as an animal model for athletic-related disease, this is one of the first steps towards improving our understanding of similarities and differences between the genetic background of horses and humans, and further developing the horse’s potential as a model for athletic-related disease.

## Online methods

### Identification of equine genetic variation

Paired-end whole genome sequencing (WGS) was performed on 607 horses of 48 different breeds using Illumina technology. Two horses (one Standardbred and one Morgan) were excluded due to excess heterozygosity suggesting a sample issue. Therefore 605 horses of 48 different breeds were used for this analysis. As described by Durward-Akhurst et al.^38^ the aim of this project was to collect a minimum of 15 individuals per breed for 10 target breeds (Arabian, Belgian, Clydesdale, Icelandic, Morgan, Quarter Horse, Shetland, Standardbred, Thoroughbred, and Welsh Pony) that represent major groups of worldwide equine genetic diversity^10^. Ultimately, we collected 17 or more individuals from 12 breeds (Arabian, Belgian, Clydesdale, Franches-Montagnes, Icelandic, Morgan, Quarter Horse, Shetland, Standardbred, Thoroughbred, Warmblood, and Welsh Pony). Mapping and variant calling was performed using a modified version of the Genome Analysis Toolkit best practices that uses a containerized snakemake pipeline to map (BWA), variant call (GATK-haplotype caller with joint genotyping), and filter (variant quality score redistribution) the WGS^42^.

### Estimation of the predicted genetic burden

Descriptive statistics for the identified variants across the 605 horses and for each individual were created using BCFtools^81^. Variant annotation was performed using Ensembl-VEP^82^ and SnpEff^83^ with custom dictionaries based on the UCSC Golden Path version of EquCab 3.0., which includes the Y chromosome. High, moderate, and low impact variants were extracted using Ensembl-VEP filter^82^ and SnpSift^84^. Custom python scripts (https://github.com/durwa004/genetic_burden_pipeline) and BCFtools^81^ were used to manipulate output files. For both variant callers, the first impact, which is also the most deleterious was selected for downstream analysis. The predicted genetic burden was determined by extracting variants identified as high impact by both annotation programs, or high impact by one annotation program and moderate impact by the other annotation program. The predicted genetic burden includes LOF variants which were identified based on a previous definition of computationally predicted LOF variants. In brief, LOF variants were variants predicted to lead to a frameshift, splice site alteration, start or stop lost, and stop gained. Gene pathway clustering enrichment using Bonferroni correction for the predicted genetic burden and LOF variants was determined using DAVID v6.8^43^.

### Identification of published genetic variants

Variants reported in the Online Mendelian Inheritance in Animals (https://omia.org/home/) were investigated to determine the overall frequency and breed specific distribution in these 605 horses (Table 5, Supplementary Table 6). Only single nucleotide polymorphisms and indels $\le$ 20 base pairs in length were investigated. Variants and locations were identified during a search of the OMIA database in January 2023. Variants reported in EquCab2.0 were remapped using the NCBI remapping tool if genomic locations were reported or remapped manually if the coding variant was reported. Likely genetic disorders were pulled from the “All traits: disease and non-disease” heading. The disease-causing variants explored in this study were selected from the “Mendelian diseases: with at least one known likely causal variant” category. The non-coat color trait causing variants were selected from the “All Mendelian traits: disease and non-disease with at least one known likely causal variant” category after excluding for disease and coat color variants. The coat color variants were selected from the “All Mendelian traits: disease and non-disease” category after excluding for disease and non-coat color traits. Coat color causing and associated variants with pleiotropic effects linked to disease were included as disease variants. The disease-associated variants were selected from the “Mendelian diseases” category after excluding variants that were listed in the “with at least one known likely causal variant” category. The non-coat color trait associated variants were selected from the “All Mendelian traits: disease and non-disease” category after excluding diseases present in the “with at least one known likely causal variant” category.

Known Mendelian disease-causing variants with unexpected genotypes in this cohort, for example, a known lethal recessive trait with homozygotes in the cohort were manually checked using the Integrative Genome Viewer (IGV)^85^ and where possible the horse’s age and breed verified through our internal database or an online search of the horse’s registered name. Follow up genotyping was performed at the UC Davis Veterinary Genetics Laboratory for horses with unexpected genotypes for the Mendelian diseases that had DNA available: one of the GBED homozygotes, three of the MYHM horses (two Belgians and a Welsh Pony) and the Quarter Horse that was heterozygous for the CA variant.

### Statistics and reproducibility

Linear regression was used to determine if the variant numbers identified were associated with the depth of coverage and if there were breed differences. For the breed analyses, estimated marginal means (EMMEANs) were used due to the relationship between the number of variants identified and the depth of coverage^86^. EMMEANs allow for investigation of associations across different levels of a categorical predictor (breed), while accounting for potential confounding variables (depth of coverage). T-tests were used to compare variant types and frequencies between coding and non-coding variants. Confidence intervals (95%) were calculated for each breed. All statistical analyses were performed using R^87^ (https://github.com/durwa004/genetic_burden_pipeline/R_analysis/GB_paper.R). Significance was set at *p* < 0.05.

### Supplementary Information

Supplementary Tables.

## Data Availability

The variant-calling format file for 504 of the 605 horses in this catalog of genetic variation has been submitted to the European Variant Archive (*project ID: PRJEB47918)*. The mapping, variant calling, and filtering pipeline (https://github.com/jonahcullen/WAGS) and predicted genetic burden analysis code (https://github.com/durwa004/genetic_burden_pipeline) are available on GitHub.
