# Supplementary material for: Predicted genetic burden and frequency of phenotype-associated variants in the horse
Source: Sci Rep. 2024 Apr 10;14:8396. doi: 10.1038/s41598-024-57872-8 (PMC11006912; doi:10.1038/s41598-024-57872-8)
Supplement: Supplementary file 1 — Supplementary Tables. [file 41598_2024_57872_MOESM1_ESM.pdf]

Supplementary table 1. Breed information for the 605 horses

Breed	Number	Average DOC
Aegidienberger Paso x Icelandic	2	2
Arabian	[1] 38	17.6
Belgian	[1] 20	8.1
British Warmblood	2	14.9
Clydesdale	[1] 19	7.3
Coldblood	3	11.7
Connemara	4	5.2
Curly Trotter	2	12.2
Draft x	1	7.7
Dulmener	1	13.3
Franchese Montagne	[1] 30	13
French Trotter	10	8.9
Friesian	2	8.9
German WB	9	17.9
Haflinger	8	17.7
Hanoverian	7	14.4
Holsteiner	3	13.9
Icelandic	[1] 17	9.7
Italian trotter	1	30.6
Jeju pony	[1] 21	2.9
KWPN	1	10.3
Lipizzaner	4	17.8
Mangalarga Marchador Horse	1	12.8
Miniature Horse	4	8.6
Missouri Fox Trotter x	1	31.8
Mongolian	10	7.3
Morgan	[1] 21	8.5
Native Mongolian Chakouyi Horse	1	12.1
Norwegian Fjord	1	12.5
Oldenberg	2	15.7
POA	1	24.1
Paint	1	20.4

[1] Breeds with 17 or more horses.

**Supplementary table 1.** Breed information for the 605 horses		
Percheron	3	15.2
Quarter Horse	[1] 104	12.2
Quarter Horse x	1	15.7
Saddle Trotter	2	15.3
Saxon-Thuringian Heavy Warmblood	1	13.4
Shetland	[1] 55	5.4
Sorraia	3	11.6
Sports Horse	2	26
Standardbred	[1] 58	10.6
Swiss Warmblood	3	10.4
Tennessee Walking Horse	5	16.7
Thoroughbred	[1] 75	10.1
Thoroughbred x	1	8
Trakehner	2	14.9
UK Warmblood	1	16.2
Unknown	8	12.2
WP	[1] 20	6.8
Warmblood	2	20.9
Westphalian	2	16.2
Yakut	9	17.4

[1] Breeds with 17 or more horses.

Supplementary table 2. Ensembl gene IDs containing >5 genetic burden variants

Ensembl gene ID	Number
ENSECAG00000000048	6
ENSECAG00000000239	6
ENSECAG00000000384	6
ENSECAG00000000397	6
ENSECAG00000000435	6
ENSECAG00000000464	6
ENSECAG00000000529	6
ENSECAG00000000548	6
ENSECAG00000000701	6
ENSECAG00000000753	6
ENSECAG00000000755	6
ENSECAG00000000934	6
ENSECAG00000000972	6
ENSECAG00000001604	6
ENSECAG00000001845	6
ENSECAG00000002115	6
ENSECAG00000002541	6
ENSECAG00000003485	6
ENSECAG00000003619	6
ENSECAG00000003862	6
ENSECAG00000004053	6
ENSECAG00000004234	6
ENSECAG00000004523	6
ENSECAG00000004906	6
ENSECAG00000004912	6
ENSECAG00000004957	6
ENSECAG00000005271	6
ENSECAG00000005517	6
ENSECAG00000005580	6
ENSECAG00000005731	6
ENSECAG00000005805	6
ENSECAG00000005906	6

Supplementary table 2. Ensembl gene IDs containing >5 genetic burden variants

ENSECAG00000005990	6
ENSECAG00000006306	6
ENSECAG00000006745	6
ENSECAG00000006787	6
ENSECAG00000006906	6
ENSECAG00000006913	6
ENSECAG00000007169	6
ENSECAG00000007322	6
ENSECAG00000007374	6
ENSECAG00000007422	6
ENSECAG00000007483	6
ENSECAG00000007493	6
ENSECAG00000007706	6
ENSECAG00000007743	6
ENSECAG00000007750	6
ENSECAG00000007768	6
ENSECAG00000008016	6
ENSECAG00000008107	6
ENSECAG00000008199	6
ENSECAG00000008264	6
ENSECAG00000008283	6
ENSECAG00000008405	6
ENSECAG00000008470	6
ENSECAG00000008511	6
ENSECAG00000008522	6
ENSECAG00000008571	6
ENSECAG00000008600	6
ENSECAG00000008705	6
ENSECAG00000008773	6
ENSECAG00000008893	6
ENSECAG00000008987	6
ENSECAG00000009111	6
ENSECAG00000009128	6

**Supplementary table 2.** Ensembl gene IDs containing >5 genetic burden variants	
ENSECAG00000009189	6
ENSECAG00000009218	6
ENSECAG00000009378	6
ENSECAG00000009651	6
ENSECAG00000009658	6
ENSECAG00000009711	6
ENSECAG00000009751	6
ENSECAG00000009828	6
ENSECAG00000010062	6
ENSECAG00000010081	6
ENSECAG00000010227	6
ENSECAG00000010242	6
ENSECAG00000010268	6
ENSECAG00000010353	6
ENSECAG00000010354	6
ENSECAG00000010431	6
ENSECAG00000010736	6
ENSECAG00000010836	6
ENSECAG00000010862	6
ENSECAG00000010934	6
ENSECAG00000010984	6
ENSECAG00000011129	6
ENSECAG00000011181	6
ENSECAG00000011466	6
ENSECAG00000011569	6
ENSECAG00000011582	6
ENSECAG00000011686	6
ENSECAG00000011885	6
ENSECAG00000011933	6
ENSECAG00000011966	6
ENSECAG00000011991	6
ENSECAG00000012020	6
ENSECAG00000012089	6

Supplementary table 2. Ensembl gene IDs containing >5 genetic burden variants

ENSECAG00000012099	6
ENSECAG00000012186	6
ENSECAG00000012574	6
ENSECAG00000012586	6
ENSECAG00000012597	6
ENSECAG00000012668	6
ENSECAG00000012941	6
ENSECAG00000012948	6
ENSECAG00000013274	6
ENSECAG00000013285	6
ENSECAG00000013375	6
ENSECAG00000013377	6
ENSECAG00000013443	6
ENSECAG00000013475	6
ENSECAG00000013655	6
ENSECAG00000013923	6
ENSECAG00000014108	6
ENSECAG00000014112	6
ENSECAG00000014304	6
ENSECAG00000014428	6
ENSECAG00000014524	6
ENSECAG00000014533	6
ENSECAG00000014617	6
ENSECAG00000014662	6
ENSECAG00000014743	6
ENSECAG00000014766	6
ENSECAG00000014779	6
ENSECAG00000014808	6
ENSECAG00000014890	6
ENSECAG00000015044	6
ENSECAG00000015087	6
ENSECAG00000015220	6
ENSECAG00000015253	6

Supplementary table 2. Ensembl gene IDs containing >5 genetic burden variants

ENSECAG00000015415	6
ENSECAG00000015633	6
ENSECAG00000015889	6
ENSECAG00000015910	6
ENSECAG00000015947	6
ENSECAG00000015984	6
ENSECAG00000015992	6
ENSECAG00000016102	6
ENSECAG00000016197	6
ENSECAG00000016440	6
ENSECAG00000016702	6
ENSECAG00000016724	6
ENSECAG00000016786	6
ENSECAG00000016931	6
ENSECAG00000017043	6
ENSECAG00000017074	6
ENSECAG00000017298	6
ENSECAG00000017547	6
ENSECAG00000017662	6
ENSECAG00000017894	6
ENSECAG00000018233	6
ENSECAG00000018243	6
ENSECAG00000018459	6
ENSECAG00000018460	6
ENSECAG00000018476	6
ENSECAG00000018529	6
ENSECAG00000018681	6
ENSECAG00000018702	6
ENSECAG00000019038	6
ENSECAG00000019044	6
ENSECAG00000019541	6
ENSECAG00000019559	6
ENSECAG00000019669	6

Supplementary table 2. Ensembl gene IDs containing >5 genetic burden variants

ENSECAG00000019713	6
ENSECAG00000019793	6
ENSECAG00000019950	6
ENSECAG00000020030	6
ENSECAG00000020260	6
ENSECAG00000020393	6
ENSECAG00000020463	6
ENSECAG00000020465	6
ENSECAG00000020474	6
ENSECAG00000020637	6
ENSECAG00000020716	6
ENSECAG00000020764	6
ENSECAG00000020792	6
ENSECAG00000020822	6
ENSECAG00000020872	6
ENSECAG00000021133	6
ENSECAG00000021182	6
ENSECAG00000021207	6
ENSECAG00000021331	6
ENSECAG00000021388	6
ENSECAG00000021543	6
ENSECAG00000021595	6
ENSECAG00000021653	6
ENSECAG00000021759	6
ENSECAG00000021800	6
ENSECAG00000021898	6
ENSECAG00000021916	6
ENSECAG00000022493	6
ENSECAG00000022540	6
ENSECAG00000022690	6
ENSECAG00000022703	6
ENSECAG00000022823	6
ENSECAG00000022922	6

**Supplementary table 2.** Ensembl gene IDs containing >5 genetic burden variants	
ENSECAG00000022923	6
ENSECAG00000022942	6
ENSECAG00000023016	6
ENSECAG00000023019	6
ENSECAG00000023235	6
ENSECAG00000023311	6
ENSECAG00000023814	6
ENSECAG00000023868	6
ENSECAG00000024076	6
ENSECAG00000024186	6
ENSECAG00000024348	6
ENSECAG00000024371	6
ENSECAG00000024385	6
ENSECAG00000024438	6
ENSECAG00000024761	6
ENSECAG00000024769	6
ENSECAG00000024979	6
ENSECAG00000024983	6
ENSECAG00000025029	6
ENSECAG00000025159	6
ENSECAG00000025168	6
ENSECAG00000028885	6
ENSECAG00000030750	6
ENSECAG00000030894	6
ENSECAG00000031204	6
ENSECAG00000033682	6
ENSECAG00000034046	6
ENSECAG00000034143	6
ENSECAG00000034911	6
ENSECAG00000035038	6
ENSECAG00000035205	6
ENSECAG00000035675	6
ENSECAG00000036129	6

**Supplementary table 2.** Ensembl gene IDs containing >5 genetic burden variants	
ENSECAG00000036583	6
ENSECAG00000038846	6
ENSECAG00000039434	6
ENSECAG00000040020	6
ENSECAG00000040529	6
ENSECAG00000040540	6
ENSECAG00000040891	6
ENSECAG00000041924	6
ENSECAG00000042150	6
ENSECAG00000042385	6
ENSECAG00000043020	6
ENSECAG00000043192	6
ENSECAG00000000066	7
ENSECAG00000000153	7
ENSECAG00000000207	7
ENSECAG00000000236	7
ENSECAG00000000242	7
ENSECAG00000000261	7
ENSECAG00000000296	7
ENSECAG00000000402	7
ENSECAG00000000642	7
ENSECAG00000000700	7
ENSECAG00000000823	7
ENSECAG00000000851	7
ENSECAG00000000873	7
ENSECAG00000000974	7
ENSECAG00000001358	7
ENSECAG00000001649	7
ENSECAG00000001653	7
ENSECAG00000002675	7
ENSECAG00000002831	7
ENSECAG00000002919	7
ENSECAG00000003634	7

Supplementary table 2. Ensembl gene IDs containing >5 genetic burden variants

ENSECAG00000003790	7
ENSECAG00000004913	7
ENSECAG00000005619	7
ENSECAG00000005925	7
ENSECAG00000006467	7
ENSECAG00000006573	7
ENSECAG00000006795	7
ENSECAG00000006949	7
ENSECAG00000007134	7
ENSECAG00000007150	7
ENSECAG00000007152	7
ENSECAG00000007228	7
ENSECAG00000007248	7
ENSECAG00000007464	7
ENSECAG00000007481	7
ENSECAG00000007644	7
ENSECAG00000007914	7
ENSECAG00000008008	7
ENSECAG00000008051	7
ENSECAG00000008057	7
ENSECAG00000008096	7
ENSECAG00000008102	7
ENSECAG00000008141	7
ENSECAG00000008421	7
ENSECAG00000008485	7
ENSECAG00000008499	7
ENSECAG00000008984	7
ENSECAG00000008993	7
ENSECAG00000009171	7
ENSECAG00000009302	7
ENSECAG00000009513	7
ENSECAG00000009628	7
ENSECAG00000009688	7

Supplementary table 2. Ensembl gene IDs containing >5 genetic burden variants

ENSECAG00000009904	7
ENSECAG00000010050	7
ENSECAG00000010201	7
ENSECAG00000010285	7
ENSECAG00000010540	7
ENSECAG00000010977	7
ENSECAG00000010986	7
ENSECAG00000011422	7
ENSECAG00000011451	7
ENSECAG00000011452	7
ENSECAG00000011751	7
ENSECAG00000011882	7
ENSECAG00000011917	7
ENSECAG00000011923	7
ENSECAG00000012229	7
ENSECAG00000012353	7
ENSECAG00000012389	7
ENSECAG00000012753	7
ENSECAG00000012816	7
ENSECAG00000012822	7
ENSECAG00000013080	7
ENSECAG00000013124	7
ENSECAG00000013174	7
ENSECAG00000013180	7
ENSECAG00000013193	7
ENSECAG00000013409	7
ENSECAG00000013424	7
ENSECAG00000013578	7
ENSECAG00000013718	7
ENSECAG00000013853	7
ENSECAG00000014119	7
ENSECAG00000014218	7
ENSECAG00000014377	7

**Supplementary table 2.** Ensembl gene IDs containing >5 genetic burden variants	
ENSECAG00000014491	7
ENSECAG00000014531	7
ENSECAG00000014620	7
ENSECAG00000014789	7
ENSECAG00000015133	7
ENSECAG00000015214	7
ENSECAG00000015258	7
ENSECAG00000015336	7
ENSECAG00000015543	7
ENSECAG00000015580	7
ENSECAG00000015740	7
ENSECAG00000015843	7
ENSECAG00000015999	7
ENSECAG00000016082	7
ENSECAG00000016298	7
ENSECAG00000016340	7
ENSECAG00000016401	7
ENSECAG00000016432	7
ENSECAG00000016458	7
ENSECAG00000016491	7
ENSECAG00000017145	7
ENSECAG00000017221	7
ENSECAG00000017344	7
ENSECAG00000017376	7
ENSECAG00000017448	7
ENSECAG00000017776	7
ENSECAG00000017840	7
ENSECAG00000017887	7
ENSECAG00000018035	7
ENSECAG00000018428	7
ENSECAG00000019111	7
ENSECAG00000019114	7
ENSECAG00000019192	7

Supplementary table 2. Ensembl gene IDs containing >5 genetic burden variants

ENSECAG00000019297	7
ENSECAG00000019329	7
ENSECAG00000019381	7
ENSECAG00000019497	7
ENSECAG00000019545	7
ENSECAG00000019736	7
ENSECAG00000020175	7
ENSECAG00000020298	7
ENSECAG00000020447	7
ENSECAG00000020547	7
ENSECAG00000020582	7
ENSECAG00000020633	7
ENSECAG00000020638	7
ENSECAG00000020645	7
ENSECAG00000020881	7
ENSECAG00000021031	7
ENSECAG00000021085	7
ENSECAG00000021275	7
ENSECAG00000021443	7
ENSECAG00000021686	7
ENSECAG00000021708	7
ENSECAG00000021818	7
ENSECAG00000021840	7
ENSECAG00000022254	7
ENSECAG00000022378	7
ENSECAG00000022582	7
ENSECAG00000023061	7
ENSECAG00000023086	7
ENSECAG00000023274	7
ENSECAG00000023594	7
ENSECAG00000023800	7
ENSECAG00000023849	7
ENSECAG00000023851	7

**Supplementary table 2.** Ensembl gene IDs containing >5 genetic burden variants	
ENSECAG00000023942	7
ENSECAG00000024127	7
ENSECAG00000024196	7
ENSECAG00000024832	7
ENSECAG00000025036	7
ENSECAG00000028686	7
ENSECAG00000028845	7
ENSECAG00000030516	7
ENSECAG00000030809	7
ENSECAG00000032416	7
ENSECAG00000032898	7
ENSECAG00000033572	7
ENSECAG00000033821	7
ENSECAG00000033922	7
ENSECAG00000035221	7
ENSECAG00000035500	7
ENSECAG00000037921	7
ENSECAG00000038039	7
ENSECAG00000040077	7
ENSECAG00000041422	7
ENSECAG00000042405	7
ENSECAG00000000253	8
ENSECAG00000000644	8
ENSECAG00000001119	8
ENSECAG00000001155	8
ENSECAG00000002578	8
ENSECAG00000003259	8
ENSECAG00000004436	8
ENSECAG00000004819	8
ENSECAG00000004824	8
ENSECAG00000005417	8
ENSECAG00000005522	8
ENSECAG00000005547	8

**Supplementary table 2.** Ensembl gene IDs containing >5 genetic burden variants	
ENSECAG00000005949	8
ENSECAG00000006042	8
ENSECAG00000006457	8
ENSECAG00000007556	8
ENSECAG00000008164	8
ENSECAG00000008535	8
ENSECAG00000008711	8
ENSECAG00000008911	8
ENSECAG00000009022	8
ENSECAG00000009102	8
ENSECAG00000009847	8
ENSECAG00000009856	8
ENSECAG00000010644	8
ENSECAG00000010729	8
ENSECAG00000010812	8
ENSECAG00000011004	8
ENSECAG00000011055	8
ENSECAG00000011080	8
ENSECAG00000011497	8
ENSECAG00000011673	8
ENSECAG00000012331	8
ENSECAG00000012337	8
ENSECAG00000012365	8
ENSECAG00000012420	8
ENSECAG00000013053	8
ENSECAG00000013581	8
ENSECAG00000013613	8
ENSECAG00000013701	8
ENSECAG00000013759	8
ENSECAG00000013998	8
ENSECAG00000014251	8
ENSECAG00000014809	8
ENSECAG00000014914	8

Supplementary table 2. Ensembl gene IDs containing >5 genetic burden variants

ENSECAG00000014984	8
ENSECAG00000015137	8
ENSECAG00000015456	8
ENSECAG00000015655	8
ENSECAG00000015730	8
ENSECAG00000016010	8
ENSECAG00000016258	8
ENSECAG00000016641	8
ENSECAG00000017253	8
ENSECAG00000017908	8
ENSECAG00000018381	8
ENSECAG00000018485	8
ENSECAG00000018611	8
ENSECAG00000018775	8
ENSECAG00000018796	8
ENSECAG00000018906	8
ENSECAG00000019326	8
ENSECAG00000019802	8
ENSECAG00000019820	8
ENSECAG00000019916	8
ENSECAG00000020679	8
ENSECAG00000020699	8
ENSECAG00000020833	8
ENSECAG00000021186	8
ENSECAG00000021252	8
ENSECAG00000021471	8
ENSECAG00000021664	8
ENSECAG00000021813	8
ENSECAG00000021997	8
ENSECAG00000022413	8
ENSECAG00000022712	8
ENSECAG00000022875	8
ENSECAG00000022984	8

Supplementary table 2. Ensembl gene IDs containing >5 genetic burden variants

ENSECAG00000023034	8
ENSECAG00000023322	8
ENSECAG00000023331	8
ENSECAG00000023534	8
ENSECAG00000023656	8
ENSECAG00000023879	8
ENSECAG00000024082	8
ENSECAG00000024343	8
ENSECAG00000024558	8
ENSECAG00000024654	8
ENSECAG00000024956	8
ENSECAG00000025126	8
ENSECAG00000027819	8
ENSECAG00000028168	8
ENSECAG00000028359	8
ENSECAG00000029311	8
ENSECAG00000029681	8
ENSECAG00000031005	8
ENSECAG00000033152	8
ENSECAG00000033388	8
ENSECAG00000035267	8
ENSECAG00000038761	8
ENSECAG00000040231	8
ENSECAG00000040393	8
ENSECAG00000043734	8
ENSECAG00000000182	9
ENSECAG00000000287	9
ENSECAG00000000393	9
ENSECAG00000000505	9
ENSECAG00000000782	9
ENSECAG00000000809	9
ENSECAG00000002267	9
ENSECAG00000002892	9

**Supplementary table 2.** Ensembl gene IDs containing >5 genetic burden variants	
ENSECAG00000003079	9
ENSECAG00000005126	9
ENSECAG00000005608	9
ENSECAG00000006526	9
ENSECAG00000006885	9
ENSECAG00000007315	9
ENSECAG00000007513	9
ENSECAG00000007945	9
ENSECAG00000008134	9
ENSECAG00000008833	9
ENSECAG00000008941	9
ENSECAG00000010196	9
ENSECAG00000010373	9
ENSECAG00000010685	9
ENSECAG00000010990	9
ENSECAG00000011457	9
ENSECAG00000011591	9
ENSECAG00000011612	9
ENSECAG00000011694	9
ENSECAG00000011718	9
ENSECAG00000011995	9
ENSECAG00000012677	9
ENSECAG00000012933	9
ENSECAG00000013366	9
ENSECAG00000013931	9
ENSECAG00000014203	9
ENSECAG00000014435	9
ENSECAG00000014967	9
ENSECAG00000015338	9
ENSECAG00000015597	9
ENSECAG00000016217	9
ENSECAG00000016346	9
ENSECAG00000016447	9

**Supplementary table 2.** Ensembl gene IDs containing >5 genetic burden variants	
ENSECAG00000016782	9
ENSECAG00000017677	9
ENSECAG00000017741	9
ENSECAG00000019156	9
ENSECAG00000019590	9
ENSECAG00000020433	9
ENSECAG00000021057	9
ENSECAG00000021610	9
ENSECAG00000021698	9
ENSECAG00000021880	9
ENSECAG00000022093	9
ENSECAG00000022375	9
ENSECAG00000022544	9
ENSECAG00000022635	9
ENSECAG00000023455	9
ENSECAG00000024091	9
ENSECAG00000024461	9
ENSECAG00000024828	9
ENSECAG00000028328	9
ENSECAG00000029340	9
ENSECAG00000029414	9
ENSECAG00000029882	9
ENSECAG00000030856	9
ENSECAG00000032492	9
ENSECAG00000033354	9
ENSECAG00000037675	9
ENSECAG00000043572	9
ENSECAG00000000162	10
ENSECAG00000000291	10
ENSECAG00000003669	10
ENSECAG00000003761	10
ENSECAG00000005383	10
ENSECAG00000006557	10

**Supplementary table 2.** Ensembl gene IDs containing >5 genetic burden variants	
ENSECAG00000007763	10
ENSECAG00000008135	10
ENSECAG00000008291	10
ENSECAG00000008458	10
ENSECAG00000009142	10
ENSECAG00000009583	10
ENSECAG00000011132	10
ENSECAG00000011229	10
ENSECAG00000013039	10
ENSECAG00000013309	10
ENSECAG00000013502	10
ENSECAG00000015040	10
ENSECAG00000015531	10
ENSECAG00000015566	10
ENSECAG00000015755	10
ENSECAG00000015929	10
ENSECAG00000015940	10
ENSECAG00000016150	10
ENSECAG00000016151	10
ENSECAG00000016707	10
ENSECAG00000017064	10
ENSECAG00000017353	10
ENSECAG00000019077	10
ENSECAG00000019125	10
ENSECAG00000019154	10
ENSECAG00000019775	10
ENSECAG00000019776	10
ENSECAG00000020091	10
ENSECAG00000020181	10
ENSECAG00000021171	10
ENSECAG00000021295	10
ENSECAG00000021325	10
ENSECAG00000021657	10

**Supplementary table 2.** Ensembl gene IDs containing >5 genetic burden variants	
ENSECAG00000021742	10
ENSECAG00000022120	10
ENSECAG00000022272	10
ENSECAG00000022440	10
ENSECAG00000022453	10
ENSECAG00000022941	10
ENSECAG00000024920	10
ENSECAG00000025136	10
ENSECAG00000028421	10
ENSECAG00000030297	10
ENSECAG00000030546	10
ENSECAG00000030648	10
ENSECAG00000032353	10
ENSECAG00000033355	10
ENSECAG00000038976	10
ENSECAG00000001621	11
ENSECAG00000006544	11
ENSECAG00000007301	11
ENSECAG00000007955	11
ENSECAG00000009114	11
ENSECAG00000009635	11
ENSECAG00000009721	11
ENSECAG00000010792	11
ENSECAG00000011768	11
ENSECAG00000012335	11
ENSECAG00000012829	11
ENSECAG00000013260	11
ENSECAG00000013722	11
ENSECAG00000014452	11
ENSECAG00000014560	11
ENSECAG00000015139	11
ENSECAG00000015175	11
ENSECAG00000015514	11

Supplementary table 2. Ensembl gene IDs containing >5 genetic burden variants

ENSECAG00000015935	11
ENSECAG00000015938	11
ENSECAG00000016371	11
ENSECAG00000016883	11
ENSECAG00000017039	11
ENSECAG00000017711	11
ENSECAG00000018948	11
ENSECAG00000019162	11
ENSECAG00000020003	11
ENSECAG00000020506	11
ENSECAG00000021147	11
ENSECAG00000021225	11
ENSECAG00000021300	11
ENSECAG00000021385	11
ENSECAG00000021561	11
ENSECAG00000022546	11
ENSECAG00000022671	11
ENSECAG00000022955	11
ENSECAG00000023009	11
ENSECAG00000024518	11
ENSECAG00000028160	11
ENSECAG00000033498	11
ENSECAG00000035495	11
ENSECAG00000035604	11
ENSECAG00000037493	11
ENSECAG00000040710	11
ENSECAG00000000465	12
ENSECAG00000001129	12
ENSECAG00000001716	12
ENSECAG00000007532	12
ENSECAG00000010919	12
ENSECAG00000012232	12
ENSECAG00000014767	12

Supplementary table 2. Ensembl gene IDs containing >5 genetic burden variants

ENSECAG00000016554	12
ENSECAG00000019207	12
ENSECAG00000019675	12
ENSECAG00000020984	12
ENSECAG00000022262	12
ENSECAG00000022405	12
ENSECAG00000022500	12
ENSECAG00000030081	12
ENSECAG00000031280	12
ENSECAG00000036205	12
ENSECAG00000036352	12
ENSECAG00000038758	12
ENSECAG00000000408	13
ENSECAG00000000638	13
ENSECAG00000006899	13
ENSECAG00000008848	13
ENSECAG00000009396	13
ENSECAG00000010610	13
ENSECAG00000013103	13
ENSECAG00000016800	13
ENSECAG00000016912	13
ENSECAG00000016982	13
ENSECAG00000018555	13
ENSECAG00000019242	13
ENSECAG00000020168	13
ENSECAG00000020885	13
ENSECAG00000023231	13
ENSECAG00000024243	13
ENSECAG00000024623	13
ENSECAG00000033604	13
ENSECAG00000034383	13
ENSECAG00000000281	14
ENSECAG00000008642	14

Supplementary table 2. Ensembl gene IDs containing >5 genetic burden variants

ENSECAG00000014700	14
ENSECAG00000016283	14
ENSECAG00000017563	14
ENSECAG00000024149	14
ENSECAG00000030169	14
ENSECAG00000038257	14
ENSECAG00000041707	14
ENSECAG00000002944	15
ENSECAG00000005238	15
ENSECAG00000007442	15
ENSECAG00000014002	15
ENSECAG00000015086	15
ENSECAG00000022005	15
ENSECAG00000023501	15
ENSECAG00000024267	15
ENSECAG00000008046	16
ENSECAG00000015321	16
ENSECAG00000016212	16
ENSECAG00000018093	16
ENSECAG00000021318	16
ENSECAG00000021868	16
ENSECAG00000033575	16
ENSECAG00000007976	17
ENSECAG00000009625	17
ENSECAG00000016720	17
ENSECAG00000019932	17
ENSECAG00000038754	17
ENSECAG00000043705	17
ENSECAG00000013110	18
ENSECAG00000020358	18
ENSECAG00000022072	19
ENSECAG00000034395	19
ENSECAG00000035510	19

Supplementary table 2. Ensembl gene IDs containing >5 genetic burden variants

ENSECAG00000004409	21
ENSECAG00000017143	21
ENSECAG00000021014	21
ENSECAG00000017796	22
ENSECAG00000022820	22
ENSECAG00000030129	22
ENSECAG00000016464	23
ENSECAG00000034339	24
ENSECAG00000010774	25
ENSECAG00000024259	25
ENSECAG00000022377	27
ENSECAG00000009550	30
ENSECAG00000016123	30
ENSECAG00000042020	31
ENSECAG00000007192	41
ENSECAG00000011804	84

Supplementary table 3. Ensembl gene IDs containing a genetic burden variants at an allele frequency >5%

Ensembl gene ID	Allele frequency
ENSECAG00000012918	0.05
ENSECAG00000009630	0.05
ENSECAG00000009046	0.05
ENSECAG00000011985	0.05
ENSECAG00000020871	0.05
ENSECAG00000005586	0.05
ENSECAG00000022087	0.05
ENSECAG00000022636	0.05
ENSECAG00000027758	0.05
ENSECAG00000037403	0.05
ENSECAG00000022721	0.05
ENSECAG00000031419	0.05
ENSECAG00000022135	0.05
ENSECAG00000014805	0.05
ENSECAG00000011833	0.05
ENSECAG00000023519	0.05
ENSECAG00000028026	0.05
ENSECAG00000042035	0.05
ENSECAG00000009218	0.05
ENSECAG00000018678	0.05
ENSECAG00000035927	0.05
ENSECAG00000015446	0.05
ENSECAG00000032835	0.05
ENSECAG00000031551	0.05
ENSECAG00000018714	0.05
ENSECAG00000022745	0.05
ENSECAG00000024119	0.05
ENSECAG00000034143	0.05
ENSECAG00000034390	0.05
ENSECAG00000013898	0.05
ENSECAG00000030540	0.05
ENSECAG00000020450	0.05

**Supplementary table 3.** Ensembl gene IDs containing a genetic burden variants at an allele frequency >5%	
ENSECAG00000009488	0.05
ENSECAG00000020030	0.05
ENSECAG00000000432	0.05
ENSECAG00000006557	0.05
ENSECAG00000022938	0.05
ENSECAG00000006542	0.05
ENSECAG00000029920	0.05
ENSECAG00000034051	0.05
ENSECAG00000039161	0.05
ENSECAG00000005335	0.05
ENSECAG00000039171	0.05
ENSECAG00000015137	0.05
ENSECAG00000003235	0.05
ENSECAG00000022072	0.05
ENSECAG00000030990	0.05
ENSECAG00000010252	0.05
ENSECAG00000000925	0.06
ENSECAG00000015222	0.06
ENSECAG00000029504	0.06
ENSECAG00000015120	0.06
ENSECAG00000017187	0.06
ENSECAG00000020055	0.06
ENSECAG00000017258	0.06
ENSECAG00000003217	0.06
ENSECAG00000013049	0.06
ENSECAG00000021795	0.06
ENSECAG00000028442	0.06
ENSECAG00000002675	0.06
ENSECAG00000024225	0.06
ENSECAG00000040757	0.06
ENSECAG00000032809	0.06
ENSECAG00000035457	0.06
ENSECAG00000012816	0.06
ENSECAG00000015099	0.06

Supplementary table 3. Ensembl gene IDs containing a genetic burden variants at an allele frequency >5%

ENSECAG00000024314	0.06
ENSECAG00000031729	0.06
ENSECAG00000008745	0.06
ENSECAG00000003545	0.06
ENSECAG00000011193	0.06
ENSECAG00000016718	0.06
ENSECAG00000020618	0.06
ENSECAG00000030229	0.06
ENSECAG00000043067	0.06
ENSECAG00000030297	0.06
ENSECAG00000021007	0.06
ENSECAG00000039739	0.06
ENSECAG00000010792	0.06
ENSECAG00000016646	0.06
ENSECAG00000014526	0.06
ENSECAG00000020998	0.06
ENSECAG00000038758	0.06
ENSECAG00000018875	0.06
ENSECAG00000027819	0.06
ENSECAG00000021239	0.06
ENSECAG00000006773	0.06
ENSECAG00000016991	0.06
ENSECAG00000008397	0.06
ENSECAG00000013150	0.06
ENSECAG00000019441	0.06
ENSECAG00000021302	0.06
ENSECAG00000034677	0.06
ENSECAG00000043632	0.06
ENSECAG00000013103	0.06
ENSECAG00000037496	0.06
ENSECAG00000008776	0.06
ENSECAG00000016506	0.06
ENSECAG00000030538	0.06
ENSECAG00000032331	0.06

**Supplementary table 3.** Ensembl gene IDs containing a genetic burden variants at an allele frequency >5%	
ENSECAG00000041485	0.06
ENSECAG00000005857	0.06
ENSECAG00000006903	0.06
ENSECAG00000009610	0.06
ENSECAG00000010551	0.06
ENSECAG00000015585	0.06
ENSECAG00000030457	0.06
ENSECAG00000038787	0.06
ENSECAG00000016601	0.06
ENSECAG00000020439	0.06
ENSECAG00000039740	0.06
ENSECAG00000012425	0.06
ENSECAG00000017983	0.06
ENSECAG00000014573	0.06
ENSECAG00000010795	0.06
ENSECAG00000017755	0.06
ENSECAG00000011365	0.07
ENSECAG00000022383	0.07
ENSECAG00000027995	0.07
ENSECAG00000030822	0.07
ENSECAG00000033670	0.07
ENSECAG00000034636	0.07
ENSECAG00000019542	0.07
ENSECAG00000002784	0.07
ENSECAG00000004449	0.07
ENSECAG00000035919	0.07
ENSECAG00000038312	0.07
ENSECAG00000038706	0.07
ENSECAG00000042777	0.07
ENSECAG00000029987	0.07
ENSECAG00000022862	0.07
ENSECAG00000032340	0.07
ENSECAG00000036496	0.07
ENSECAG00000032469	0.07

Supplementary table 3. Ensembl gene IDs containing a genetic burden variants at an allele frequency >5%

ENSECAG00000001478	0.07
ENSECAG00000005238	0.07
ENSECAG00000043336	0.07
ENSECAG00000006871	0.07
ENSECAG00000001777	0.07
ENSECAG00000010240	0.07
ENSECAG00000011912	0.07
ENSECAG00000017264	0.07
ENSECAG00000022377	0.07
ENSECAG00000033992	0.07
ENSECAG00000032409	0.07
ENSECAG00000012933	0.07
ENSECAG00000003090	0.07
ENSECAG00000009694	0.07
ENSECAG00000023061	0.07
ENSECAG00000039940	0.07
ENSECAG00000023958	0.07
ENSECAG00000023526	0.07
ENSECAG00000020945	0.07
ENSECAG00000027790	0.07
ENSECAG00000034014	0.07
ENSECAG00000035154	0.07
ENSECAG00000020354	0.07
ENSECAG00000000436	0.07
ENSECAG00000019859	0.07
ENSECAG00000002053	0.07
ENSECAG00000030415	0.07
ENSECAG00000008985	0.07
ENSECAG00000021028	0.07
ENSECAG00000015962	0.07
ENSECAG00000020588	0.07
ENSECAG00000027780	0.07
ENSECAG00000034777	0.07
ENSECAG00000020657	0.07

Supplementary table 3. Ensembl gene IDs containing a genetic burden variants at an allele frequency >5%

ENSECAG00000041748	0.07
ENSECAG00000016343	0.07
ENSECAG00000038888	0.07
ENSECAG00000008576	0.07
ENSECAG00000012377	0.07
ENSECAG00000018581	0.07
ENSECAG00000022689	0.07
ENSECAG00000021013	0.07
ENSECAG00000016786	0.07
ENSECAG00000005110	0.07
ENSECAG00000030687	0.07
ENSECAG00000008005	0.08
ENSECAG00000017267	0.08
ENSECAG00000021214	0.08
ENSECAG00000020972	0.08
ENSECAG00000010614	0.08
ENSECAG00000017672	0.08
ENSECAG00000023839	0.08
ENSECAG00000034878	0.08
ENSECAG00000037292	0.08
ENSECAG00000035044	0.08
ENSECAG00000036205	0.08
ENSECAG00000009269	0.08
ENSECAG00000017892	0.08
ENSECAG00000028104	0.08
ENSECAG00000035957	0.08
ENSECAG00000007272	0.08
ENSECAG00000014379	0.08
ENSECAG00000031928	0.08
ENSECAG00000015377	0.08
ENSECAG00000008095	0.08
ENSECAG00000021853	0.08
ENSECAG00000034026	0.08
ENSECAG00000021777	0.08

Supplementary table 3. Ensembl gene IDs containing a genetic burden variants at an allele frequency >5%

ENSECAG00000014654	0.08
ENSECAG00000018552	0.08
ENSECAG00000012369	0.08
ENSECAG00000006500	0.08
ENSECAG00000014074	0.08
ENSECAG00000023855	0.08
ENSECAG00000030946	0.08
ENSECAG00000034255	0.08
ENSECAG00000006197	0.08
ENSECAG00000033995	0.08
ENSECAG00000011787	0.08
ENSECAG00000037620	0.08
ENSECAG00000033144	0.08
ENSECAG00000011774	0.08
ENSECAG00000023093	0.08
ENSECAG00000026879	0.08
ENSECAG00000037493	0.08
ENSECAG00000024186	0.08
ENSECAG00000040529	0.08
ENSECAG00000036028	0.08
ENSECAG00000040797	0.08
ENSECAG00000041335	0.08
ENSECAG00000011558	0.08
ENSECAG00000030516	0.08
ENSECAG00000006451	0.08
ENSECAG00000010474	0.09
ENSECAG00000029256	0.09
ENSECAG00000003761	0.09
ENSECAG00000038754	0.09
ENSECAG00000024997	0.09
ENSECAG00000032353	0.09
ENSECAG00000014944	0.09
ENSECAG00000015029	0.09
ENSECAG00000015105	0.09

Supplementary table 3. Ensembl gene IDs containing a genetic burden variants at an allele frequency >5%

ENSECAG00000019092	0.09
ENSECAG00000023183	0.09
ENSECAG00000034453	0.09
ENSECAG00000036606	0.09
ENSECAG00000019709	0.09
ENSECAG00000007717	0.09
ENSECAG00000010712	0.09
ENSECAG00000024380	0.09
ENSECAG00000017611	0.09
ENSECAG00000004053	0.09
ENSECAG00000009226	0.09
ENSECAG00000038173	0.09
ENSECAG00000008751	0.09
ENSECAG00000020703	0.09
ENSECAG00000000823	0.09
ENSECAG00000035098	0.09
ENSECAG00000017121	0.09
ENSECAG00000033305	0.09
ENSECAG00000013643	0.09
ENSECAG00000015401	0.09
ENSECAG00000009695	0.09
ENSECAG00000021458	0.09
ENSECAG00000029338	0.09
ENSECAG00000029465	0.09
ENSECAG00000031523	0.09
ENSECAG00000032667	0.09
ENSECAG00000033556	0.09
ENSECAG00000034681	0.09
ENSECAG00000043128	0.09
ENSECAG00000043764	0.09
ENSECAG00000022817	0.09
ENSECAG00000017717	0.09
ENSECAG00000017222	0.09
ENSECAG00000003531	0.09

Supplementary table 3. Ensembl gene IDs containing a genetic burden variants at an allele frequency >5%

ENSECAG00000002746	0.09
ENSECAG00000036019	0.09
ENSECAG00000035001	0.09
ENSECAG00000021764	0.09
ENSECAG00000023276	0.09
ENSECAG00000042660	0.09
ENSECAG00000019802	0.09
ENSECAG00000022212	0.09
ENSECAG00000019895	0.10
ENSECAG00000028053	0.10
ENSECAG00000005852	0.10
ENSECAG00000034109	0.10
ENSECAG00000001316	0.10
ENSECAG00000008965	0.10
ENSECAG00000018571	0.10
ENSECAG00000019429	0.10
ENSECAG00000039088	0.10
ENSECAG00000039452	0.10
ENSECAG00000023922	0.10
ENSECAG00000014533	0.10
ENSECAG00000006540	0.10
ENSECAG00000035205	0.10
ENSECAG00000009625	0.10
ENSECAG00000015764	0.10
ENSECAG00000012766	0.10
ENSECAG00000017247	0.10
ENSECAG00000035614	0.10
ENSECAG00000039293	0.10
ENSECAG00000031768	0.10
ENSECAG00000003259	0.10
ENSECAG00000020898	0.10
ENSECAG00000021893	0.10
ENSECAG00000043099	0.10
ENSECAG00000006467	0.10

Supplementary table 3. Ensembl gene IDs containing a genetic burden variants at an allele frequency >5%

ENSECAG00000043572	0.10
ENSECAG00000022448	0.10
ENSECAG00000035055	0.10
ENSECAG00000020556	0.10
ENSECAG00000001077	0.10
ENSECAG00000011301	0.10
ENSECAG00000016016	0.10
ENSECAG00000023699	0.10
ENSECAG00000024117	0.10
ENSECAG00000037073	0.10
ENSECAG00000041788	0.10
ENSECAG00000023978	0.10
ENSECAG00000015188	0.10
ENSECAG00000035536	0.10
ENSECAG00000016107	0.11
ENSECAG00000008283	0.11
ENSECAG00000015731	0.11
ENSECAG00000020493	0.11
ENSECAG00000024315	0.11
ENSECAG00000017546	0.11
ENSECAG00000033766	0.11
ENSECAG00000037876	0.11
ENSECAG00000039434	0.11
ENSECAG00000007303	0.11
ENSECAG00000018762	0.11
ENSECAG00000022403	0.11
ENSECAG00000034752	0.11
ENSECAG00000040570	0.11
ENSECAG00000024828	0.11
ENSECAG00000037404	0.11
ENSECAG00000031201	0.11
ENSECAG00000019495	0.11
ENSECAG00000031615	0.11
ENSECAG00000002749	0.11

Supplementary table 3. Ensembl gene IDs containing a genetic burden variants at an allele frequency >5%

ENSECAG00000013676	0.11
ENSECAG00000012283	0.11
ENSECAG00000036707	0.11
ENSECAG00000026864	0.11
ENSECAG00000028131	0.11
ENSECAG00000001114	0.11
ENSECAG00000002830	0.11
ENSECAG00000032801	0.11
ENSECAG00000035495	0.11
ENSECAG00000003681	0.11
ENSECAG00000019297	0.11
ENSECAG00000018842	0.11
ENSECAG00000029066	0.11
ENSECAG00000022264	0.11
ENSECAG00000023327	0.11
ENSECAG00000009150	0.12
ENSECAG00000040808	0.12
ENSECAG00000001770	0.12
ENSECAG00000012693	0.12
ENSECAG00000009084	0.12
ENSECAG00000012942	0.12
ENSECAG00000019292	0.12
ENSECAG00000024165	0.12
ENSECAG00000007462	0.12
ENSECAG00000012909	0.12
ENSECAG00000019036	0.12
ENSECAG00000029997	0.12
ENSECAG00000008109	0.12
ENSECAG00000023128	0.12
ENSECAG00000018441	0.12
ENSECAG00000020345	0.12
ENSECAG00000024134	0.12
ENSECAG00000022982	0.12
ENSECAG00000026814	0.12

Supplementary table 3. Ensembl gene IDs containing a genetic burden variants at an allele frequency >5%

ENSECAG00000015340	0.12
ENSECAG00000022114	0.12
ENSECAG00000028383	0.12
ENSECAG00000029752	0.12
ENSECAG00000034627	0.12
ENSECAG00000014253	0.12
ENSECAG00000009496	0.12
ENSECAG00000007124	0.12
ENSECAG00000033607	0.12
ENSECAG00000006098	0.13
ENSECAG00000007033	0.13
ENSECAG00000013762	0.13
ENSECAG00000039302	0.13
ENSECAG00000033593	0.13
ENSECAG00000035033	0.13
ENSECAG00000032598	0.13
ENSECAG00000013545	0.13
ENSECAG00000038252	0.13
ENSECAG00000018319	0.13
ENSECAG00000036863	0.13
ENSECAG00000013872	0.13
ENSECAG00000022664	0.13
ENSECAG00000024473	0.13
ENSECAG00000039481	0.13
ENSECAG00000006414	0.13
ENSECAG00000011927	0.13
ENSECAG00000018908	0.13
ENSECAG00000012974	0.13
ENSECAG00000012864	0.13
ENSECAG00000025135	0.13
ENSECAG00000015952	0.13
ENSECAG00000017448	0.13
ENSECAG00000029898	0.14
ENSECAG00000004406	0.14

Supplementary table 3. Ensembl gene IDs containing a genetic burden variants at an allele frequency >5%

ENSECAG00000043100	0.14
ENSECAG00000036345	0.14
ENSECAG00000013812	0.14
ENSECAG00000002719	0.14
ENSECAG00000007188	0.14
ENSECAG00000008184	0.14
ENSECAG00000005353	0.14
ENSECAG00000019144	0.14
ENSECAG00000023807	0.14
ENSECAG00000039617	0.14
ENSECAG00000038375	0.14
ENSECAG00000038457	0.14
ENSECAG00000023180	0.14
ENSECAG00000031100	0.14
ENSECAG00000019827	0.14
ENSECAG00000018863	0.14
ENSECAG00000017803	0.14
ENSECAG00000002143	0.14
ENSECAG00000012413	0.14
ENSECAG00000023412	0.14
ENSECAG00000001895	0.14
ENSECAG00000009216	0.14
ENSECAG00000018964	0.14
ENSECAG00000015109	0.15
ENSECAG00000021712	0.15
ENSECAG00000009528	0.15
ENSECAG00000033689	0.15
ENSECAG00000041277	0.15
ENSECAG00000017555	0.15
ENSECAG00000015344	0.15
ENSECAG00000007957	0.15
ENSECAG00000023078	0.15
ENSECAG00000020955	0.15
ENSECAG00000035072	0.15

Supplementary table 3. Ensembl gene IDs containing a genetic burden variants at an allele frequency >5%

ENSECAG00000000702	0.15
ENSECAG00000005803	0.15
ENSECAG00000012514	0.15
ENSECAG00000021588	0.15
ENSECAG00000006864	0.15
ENSECAG00000016614	0.15
ENSECAG00000034914	0.15
ENSECAG00000016201	0.15
ENSECAG00000022808	0.16
ENSECAG00000007204	0.16
ENSECAG00000015680	0.16
ENSECAG00000008177	0.16
ENSECAG00000010552	0.16
ENSECAG00000042684	0.16
ENSECAG00000036786	0.16
ENSECAG00000023247	0.16
ENSECAG00000021152	0.16
ENSECAG00000019449	0.16
ENSECAG00000028236	0.16
ENSECAG00000019355	0.16
ENSECAG00000013725	0.17
ENSECAG00000025173	0.17
ENSECAG00000028617	0.17
ENSECAG00000021402	0.17
ENSECAG00000037237	0.17
ENSECAG00000042547	0.17
ENSECAG00000002715	0.17
ENSECAG00000039540	0.17
ENSECAG00000016536	0.17
ENSECAG00000005865	0.17
ENSECAG00000000820	0.17
ENSECAG00000004795	0.17
ENSECAG00000003159	0.17
ENSECAG00000009131	0.17

Supplementary table 3. Ensembl gene IDs containing a genetic burden variants at an allele frequency >5%

ENSECAG00000008747	0.17
ENSECAG00000012878	0.17
ENSECAG00000036081	0.17
ENSECAG00000042722	0.17
ENSECAG00000011401	0.17
ENSECAG00000000790	0.17
ENSECAG00000030390	0.17
ENSECAG00000011608	0.18
ENSECAG00000043617	0.18
ENSECAG00000021776	0.18
ENSECAG00000017791	0.18
ENSECAG00000000099	0.18
ENSECAG00000014969	0.18
ENSECAG00000027833	0.18
ENSECAG00000029257	0.18
ENSECAG00000020559	0.18
ENSECAG00000041237	0.18
ENSECAG00000019664	0.19
ENSECAG00000035432	0.19
ENSECAG00000028592	0.19
ENSECAG00000015532	0.19
ENSECAG00000015766	0.19
ENSECAG00000015642	0.19
ENSECAG00000002989	0.19
ENSECAG00000005515	0.19
ENSECAG00000017689	0.19
ENSECAG00000021630	0.19
ENSECAG00000022251	0.19
ENSECAG00000013212	0.19
ENSECAG00000021118	0.19
ENSECAG00000036907	0.19
ENSECAG00000009412	0.19
ENSECAG00000022152	0.19
ENSECAG00000033265	0.19

Supplementary table 3. Ensembl gene IDs containing a genetic burden variants at an allele frequency >5%

ENSECAG00000003398	0.19
ENSECAG00000041459	0.19
ENSECAG00000009647	0.19
ENSECAG00000000882	0.20
ENSECAG00000023266	0.20
ENSECAG00000033981	0.20
ENSECAG00000028047	0.20
ENSECAG00000020341	0.20
ENSECAG00000028505	0.20
ENSECAG00000023783	0.20
ENSECAG00000012924	0.20
ENSECAG00000023416	0.20
ENSECAG00000024874	0.20
ENSECAG00000015232	0.20
ENSECAG00000011222	0.21
ENSECAG00000036793	0.21
ENSECAG00000014841	0.21
ENSECAG00000026818	0.21
ENSECAG00000008246	0.21
ENSECAG00000030155	0.21
ENSECAG00000043084	0.21
ENSECAG00000016943	0.21
ENSECAG00000043756	0.21
ENSECAG00000039616	0.21
ENSECAG00000014426	0.22
ENSECAG00000021493	0.22
ENSECAG00000015517	0.22
ENSECAG00000009756	0.22
ENSECAG00000007860	0.22
ENSECAG00000012383	0.22
ENSECAG00000000622	0.22
ENSECAG00000022846	0.23
ENSECAG00000024608	0.23
ENSECAG00000018570	0.23

Supplementary table 3. Ensembl gene IDs containing a genetic burden variants at an allele frequency >5%

ENSECAG00000040400	0.23
ENSECAG00000031380	0.23
ENSECAG00000035085	0.23
ENSECAG00000014463	0.23
ENSECAG00000009167	0.23
ENSECAG00000004510	0.24
ENSECAG00000037320	0.24
ENSECAG00000016230	0.24
ENSECAG00000025150	0.24
ENSECAG00000040269	0.24
ENSECAG00000000778	0.24
ENSECAG00000014656	0.24
ENSECAG00000032595	0.24
ENSECAG00000015097	0.24
ENSECAG00000010649	0.24
ENSECAG00000037110	0.24
ENSECAG00000003512	0.25
ENSECAG00000022451	0.25
ENSECAG00000037747	0.25
ENSECAG00000036003	0.25
ENSECAG00000024747	0.25
ENSECAG00000028315	0.25
ENSECAG00000014289	0.25
ENSECAG00000012521	0.25
ENSECAG00000001439	0.26
ENSECAG00000012785	0.26
ENSECAG00000021472	0.26
ENSECAG00000022634	0.26
ENSECAG00000038155	0.26
ENSECAG00000002814	0.26
ENSECAG00000010934	0.26
ENSECAG00000010617	0.26
ENSECAG00000016894	0.26
ENSECAG00000022556	0.26

Supplementary table 3. Ensembl gene IDs containing a genetic burden variants at an allele frequency >5%

ENSECAG00000033720	0.26
ENSECAG00000017225	0.27
ENSECAG00000004270	0.27
ENSECAG00000034010	0.27
ENSECAG00000008022	0.27
ENSECAG00000039917	0.27
ENSECAG00000003631	0.27
ENSECAG00000001802	0.27
ENSECAG00000028500	0.27
ENSECAG00000031267	0.27
ENSECAG00000016772	0.27
ENSECAG00000017092	0.28
ENSECAG00000030870	0.28
ENSECAG00000024600	0.28
ENSECAG00000028627	0.28
ENSECAG00000017630	0.28
ENSECAG00000020173	0.28
ENSECAG00000020239	0.28
ENSECAG00000024568	0.28
ENSECAG00000009771	0.29
ENSECAG00000015156	0.29
ENSECAG00000028166	0.29
ENSECAG00000039252	0.29
ENSECAG00000018635	0.29
ENSECAG00000034890	0.29
ENSECAG00000011090	0.29
ENSECAG00000024835	0.29
ENSECAG00000019039	0.29
ENSECAG00000016613	0.29
ENSECAG00000005538	0.30
ENSECAG00000014839	0.30
ENSECAG00000032378	0.30
ENSECAG00000017037	0.30
ENSECAG00000017826	0.30

Supplementary table 3. Ensembl gene IDs containing a genetic burden variants at an allele frequency >5%

ENSECAG00000039724	0.30
ENSECAG00000032656	0.31
ENSECAG00000007612	0.31
ENSECAG00000010811	0.31
ENSECAG00000014327	0.31
ENSECAG00000022085	0.31
ENSECAG00000014711	0.31
ENSECAG00000028445	0.32
ENSECAG00000010643	0.32
ENSECAG00000015003	0.32
ENSECAG00000036594	0.32
ENSECAG00000004241	0.33
ENSECAG00000030484	0.33
ENSECAG00000036375	0.33
ENSECAG00000007603	0.33
ENSECAG00000007192	0.33
ENSECAG00000035012	0.33
ENSECAG00000038600	0.33
ENSECAG00000005924	0.34
ENSECAG00000010236	0.34
ENSECAG00000006195	0.34
ENSECAG00000037215	0.34
ENSECAG00000011190	0.35
ENSECAG00000003430	0.35
ENSECAG00000017087	0.35
ENSECAG00000014440	0.36
ENSECAG00000016814	0.36
ENSECAG00000022297	0.36
ENSECAG00000031548	0.36
ENSECAG00000017907	0.37
ENSECAG00000014585	0.37
ENSECAG00000025154	0.37
ENSECAG00000021196	0.38
ENSECAG00000005649	0.39

Supplementary table 3. Ensembl gene IDs containing a genetic burden variants at an allele frequency >5%

ENSECAG00000011079	0.40
ENSECAG00000035776	0.40
ENSECAG00000013708	0.40
ENSECAG00000008850	0.41
ENSECAG00000025038	0.41
ENSECAG00000029891	0.41
ENSECAG00000039092	0.41
ENSECAG00000013903	0.41
ENSECAG00000016521	0.42
ENSECAG00000033857	0.42
ENSECAG00000034469	0.42
ENSECAG00000015329	0.43
ENSECAG00000015945	0.43
ENSECAG00000033923	0.43
ENSECAG00000021316	0.43
ENSECAG00000016698	0.44
ENSECAG00000037799	0.44
ENSECAG00000019625	0.44
ENSECAG00000021344	0.45
ENSECAG00000017519	0.46
ENSECAG00000043476	0.46
ENSECAG00000035208	0.47
ENSECAG00000016989	0.47
ENSECAG00000010440	0.47
ENSECAG00000021649	0.49
ENSECAG00000020682	0.50
ENSECAG00000022609	0.51
ENSECAG00000039152	0.52
ENSECAG00000024146	0.53
ENSECAG00000043325	0.53
ENSECAG00000030750	0.54
ENSECAG00000032770	0.54
ENSECAG00000001795	0.54
ENSECAG00000031568	0.54

Supplementary table 3. Ensembl gene IDs containing a genetic burden variants at an allele frequency >5%

ENSECAG00000036676	0.54
ENSECAG00000036856	0.56
ENSECAG00000042911	0.56
ENSECAG00000010626	0.57
ENSECAG00000011094	0.57
ENSECAG00000022993	0.59
ENSECAG00000014784	0.62
ENSECAG00000016969	0.62
ENSECAG00000035877	0.62
ENSECAG00000034443	0.65
ENSECAG00000010271	0.66
ENSECAG00000035289	0.66
ENSECAG00000036718	0.67
ENSECAG00000038480	0.67
ENSECAG00000032890	0.68
ENSECAG00000036901	0.68
ENSECAG00000041059	0.69
ENSECAG00000018661	0.69
ENSECAG00000036544	0.69
ENSECAG00000028106	0.71
ENSECAG00000031545	0.72
ENSECAG00000007936	0.73
ENSECAG00000007511	0.73
ENSECAG00000010758	0.73
ENSECAG00000014006	0.74
ENSECAG00000035736	0.74
ENSECAG00000012110	0.74
ENSECAG00000022796	0.74
ENSECAG00000012463	0.76
ENSECAG00000016526	0.77
ENSECAG00000007667	0.78
ENSECAG00000024895	0.78
ENSECAG00000014769	0.78
ENSECAG00000013126	0.78

Supplementary table 3. Ensembl gene IDs containing a genetic burden variants at an allele frequency >5%

ENSECAG00000034616	0.79
ENSECAG00000002259	0.81
ENSECAG00000032170	0.81
ENSECAG00000013289	0.82
ENSECAG00000000927	0.83
ENSECAG00000004321	0.85
ENSECAG00000015417	0.85

Supplementary table 4. Ensembl gene IDs containing >5 LOF variants

Ensembl gene ID	Number
ENSECAG00000000066	6
ENSECAG00000000162	8
ENSECAG00000000253	7
ENSECAG00000000281	11
ENSECAG00000000287	8
ENSECAG00000000291	7
ENSECAG00000000296	6
ENSECAG00000000393	7
ENSECAG00000000408	10
ENSECAG00000000505	6
ENSECAG00000000638	9
ENSECAG00000000700	6
ENSECAG00000000809	8
ENSECAG00000000873	6
ENSECAG00000000972	6
ENSECAG00000001119	8
ENSECAG00000001129	8
ENSECAG00000001358	6
ENSECAG00000001653	7
ENSECAG00000001716	9
ENSECAG00000002267	6
ENSECAG00000002578	7
ENSECAG00000002675	6
ENSECAG00000002892	9
ENSECAG00000002944	13
ENSECAG00000003079	6
ENSECAG00000003619	6
ENSECAG00000003761	8
ENSECAG00000004053	6
ENSECAG00000004409	10
ENSECAG00000004523	6
ENSECAG00000004819	6

Supplementary table 4. Ensembl gene IDs containing >5 LOF variants

ENSECAG00000004906	6
ENSECAG00000004913	6
ENSECAG00000005238	14
ENSECAG00000005383	7
ENSECAG00000005417	6
ENSECAG00000005522	7
ENSECAG00000005547	7
ENSECAG00000005580	6
ENSECAG00000005608	8
ENSECAG00000005925	6
ENSECAG00000005949	7
ENSECAG00000006042	7
ENSECAG00000006457	6
ENSECAG00000006467	6
ENSECAG00000006526	8
ENSECAG00000006544	10
ENSECAG00000006557	8
ENSECAG00000006573	6
ENSECAG00000006745	6
ENSECAG00000006787	6
ENSECAG00000006885	7
ENSECAG00000006899	9
ENSECAG00000007134	6
ENSECAG00000007150	7
ENSECAG00000007192	31
ENSECAG00000007248	6
ENSECAG00000007301	7
ENSECAG00000007374	6
ENSECAG00000007442	9
ENSECAG00000007464	6
ENSECAG00000007481	6
ENSECAG00000007513	9
ENSECAG00000007644	6
ENSECAG00000007706	6

Supplementary table 4. Ensembl gene IDs containing >5 LOF variants

ENSECAG00000007763	7
ENSECAG00000007768	6
ENSECAG00000007955	9
ENSECAG00000007976	12
ENSECAG00000008008	6
ENSECAG00000008046	12
ENSECAG00000008051	7
ENSECAG00000008107	6
ENSECAG00000008134	6
ENSECAG00000008291	6
ENSECAG00000008458	6
ENSECAG00000008470	6
ENSECAG00000008485	6
ENSECAG00000008511	6
ENSECAG00000008535	6
ENSECAG00000008642	13
ENSECAG00000008833	6
ENSECAG00000008848	12
ENSECAG00000008893	6
ENSECAG00000008911	6
ENSECAG00000008941	6
ENSECAG00000009102	7
ENSECAG00000009111	6
ENSECAG00000009114	11
ENSECAG00000009142	9
ENSECAG00000009171	6
ENSECAG00000009218	6
ENSECAG00000009302	6
ENSECAG00000009396	12
ENSECAG00000009550	27
ENSECAG00000009583	9
ENSECAG00000009625	16
ENSECAG00000009635	8
ENSECAG00000009721	8

Supplementary table 4. Ensembl gene IDs containing >5 LOF variants

ENSECAG00000009751	6
ENSECAG00000009828	6
ENSECAG00000009847	6
ENSECAG00000009856	7
ENSECAG00000009904	6
ENSECAG00000010050	6
ENSECAG00000010196	6
ENSECAG00000010201	6
ENSECAG00000010285	7
ENSECAG00000010353	6
ENSECAG00000010610	7
ENSECAG00000010685	6
ENSECAG00000010774	17
ENSECAG00000010792	6
ENSECAG00000010812	6
ENSECAG00000010919	11
ENSECAG00000010977	6
ENSECAG00000010986	7
ENSECAG00000010990	7
ENSECAG00000011055	8
ENSECAG00000011080	7
ENSECAG00000011132	7
ENSECAG00000011229	7
ENSECAG00000011451	6
ENSECAG00000011457	8
ENSECAG00000011612	8
ENSECAG00000011694	8
ENSECAG00000011718	6
ENSECAG00000011768	7
ENSECAG00000011804	70
ENSECAG00000011933	6
ENSECAG00000011995	7
ENSECAG00000012099	6
ENSECAG00000012186	6

Supplementary table 4. Ensembl gene IDs containing >5 LOF variants

ENSECAG00000012232	7
ENSECAG00000012335	7
ENSECAG00000012353	6
ENSECAG00000012389	6
ENSECAG00000012574	6
ENSECAG00000012816	6
ENSECAG00000012829	8
ENSECAG00000012933	8
ENSECAG00000013039	9
ENSECAG00000013053	7
ENSECAG00000013080	7
ENSECAG00000013103	9
ENSECAG00000013110	13
ENSECAG00000013124	6
ENSECAG00000013260	7
ENSECAG00000013274	6
ENSECAG00000013309	6
ENSECAG00000013366	6
ENSECAG00000013581	6
ENSECAG00000013718	6
ENSECAG00000013722	7
ENSECAG00000013759	6
ENSECAG00000013923	6
ENSECAG00000013931	6
ENSECAG00000013998	7
ENSECAG00000014002	11
ENSECAG00000014203	9
ENSECAG00000014251	7
ENSECAG00000014304	6
ENSECAG00000014377	6
ENSECAG00000014435	8
ENSECAG00000014452	9
ENSECAG00000014531	7
ENSECAG00000014533	6

**Supplementary table 4.** Ensembl gene IDs containing >5 LOF variants	
ENSECAG00000014617	6
ENSECAG00000014620	6
ENSECAG00000014700	11
ENSECAG00000014767	9
ENSECAG00000014809	7
ENSECAG00000014914	6
ENSECAG00000014967	7
ENSECAG00000014984	7
ENSECAG00000015040	9
ENSECAG00000015044	6
ENSECAG00000015086	13
ENSECAG00000015087	6
ENSECAG00000015139	9
ENSECAG00000015220	6
ENSECAG00000015258	7
ENSECAG00000015321	9
ENSECAG00000015514	8
ENSECAG00000015580	7
ENSECAG00000015843	6
ENSECAG00000015910	6
ENSECAG00000015929	7
ENSECAG00000015935	10
ENSECAG00000015938	8
ENSECAG00000015940	10
ENSECAG00000015999	7
ENSECAG00000016010	6
ENSECAG00000016082	6
ENSECAG00000016123	16
ENSECAG00000016150	9
ENSECAG00000016151	8
ENSECAG00000016212	13
ENSECAG00000016217	8
ENSECAG00000016258	8
ENSECAG00000016283	13

Supplementary table 4. Ensembl gene IDs containing >5 LOF variants

ENSECAG00000016340	7
ENSECAG00000016346	7
ENSECAG00000016371	11
ENSECAG00000016401	6
ENSECAG00000016447	6
ENSECAG00000016464	16
ENSECAG00000016491	6
ENSECAG00000016554	9
ENSECAG00000016707	7
ENSECAG00000016720	12
ENSECAG00000016800	8
ENSECAG00000016883	8
ENSECAG00000016912	9
ENSECAG00000016982	12
ENSECAG00000017043	6
ENSECAG00000017064	6
ENSECAG00000017143	18
ENSECAG00000017145	7
ENSECAG00000017253	6
ENSECAG00000017353	9
ENSECAG00000017376	6
ENSECAG00000017448	6
ENSECAG00000017563	13
ENSECAG00000017677	7
ENSECAG00000017711	8
ENSECAG00000017741	9
ENSECAG00000017776	6
ENSECAG00000017796	15
ENSECAG00000017887	7
ENSECAG00000017908	6
ENSECAG00000018093	13
ENSECAG00000018233	6
ENSECAG00000018243	6
ENSECAG00000018381	6

**Supplementary table 4.** Ensembl gene IDs containing >5 LOF variants	
ENSECAG00000018476	6
ENSECAG00000018485	6
ENSECAG00000018529	6
ENSECAG00000018555	9
ENSECAG00000018611	6
ENSECAG00000018775	7
ENSECAG00000018948	9
ENSECAG00000019044	6
ENSECAG00000019125	8
ENSECAG00000019162	7
ENSECAG00000019207	10
ENSECAG00000019242	8
ENSECAG00000019326	7
ENSECAG00000019545	7
ENSECAG00000019590	8
ENSECAG00000019675	11
ENSECAG00000019713	6
ENSECAG00000019776	9
ENSECAG00000019916	6
ENSECAG00000019932	13
ENSECAG00000020091	7
ENSECAG00000020168	9
ENSECAG00000020175	7
ENSECAG00000020181	7
ENSECAG00000020298	6
ENSECAG00000020358	12
ENSECAG00000020433	7
ENSECAG00000020447	6
ENSECAG00000020463	6
ENSECAG00000020506	9
ENSECAG00000020645	7
ENSECAG00000020679	7
ENSECAG00000020699	8
ENSECAG00000020764	6

Supplementary table 4. Ensembl gene IDs containing >5 LOF variants

ENSECAG00000020792	6
ENSECAG00000020833	7
ENSECAG00000020885	13
ENSECAG00000020984	11
ENSECAG00000021014	18
ENSECAG00000021031	7
ENSECAG00000021057	6
ENSECAG00000021147	7
ENSECAG00000021171	6
ENSECAG00000021225	6
ENSECAG00000021252	6
ENSECAG00000021275	7
ENSECAG00000021300	11
ENSECAG00000021318	15
ENSECAG00000021325	7
ENSECAG00000021385	8
ENSECAG00000021443	7
ENSECAG00000021561	9
ENSECAG00000021657	8
ENSECAG00000021698	6
ENSECAG00000021742	10
ENSECAG00000021813	8
ENSECAG00000021818	6
ENSECAG00000021840	6
ENSECAG00000021868	14
ENSECAG00000021880	6
ENSECAG00000021898	6
ENSECAG00000021997	7
ENSECAG00000022005	12
ENSECAG00000022072	10
ENSECAG00000022093	7
ENSECAG00000022120	6
ENSECAG00000022262	6
ENSECAG00000022272	8

**Supplementary table 4.** Ensembl gene IDs containing >5 LOF variants	
ENSECAG00000022377	27
ENSECAG00000022405	11
ENSECAG00000022440	10
ENSECAG00000022453	7
ENSECAG00000022500	9
ENSECAG00000022544	9
ENSECAG00000022546	10
ENSECAG00000022635	6
ENSECAG00000022671	11
ENSECAG00000022820	18
ENSECAG00000022875	6
ENSECAG00000022941	8
ENSECAG00000022955	8
ENSECAG00000022984	7
ENSECAG00000023009	6
ENSECAG00000023016	6
ENSECAG00000023034	8
ENSECAG00000023061	7
ENSECAG00000023086	7
ENSECAG00000023231	6
ENSECAG00000023311	6
ENSECAG00000023455	8
ENSECAG00000023501	12
ENSECAG00000023594	6
ENSECAG00000023800	6
ENSECAG00000023849	6
ENSECAG00000023879	7
ENSECAG00000023942	6
ENSECAG00000024082	8
ENSECAG00000024091	6
ENSECAG00000024149	12
ENSECAG00000024243	8
ENSECAG00000024259	21
ENSECAG00000024267	6

**Supplementary table 4.** Ensembl gene IDs containing >5 LOF variants	
ENSECAG00000024343	7
ENSECAG00000024438	6
ENSECAG00000024461	6
ENSECAG00000024518	6
ENSECAG00000024558	8
ENSECAG00000024623	8
ENSECAG00000024654	7
ENSECAG00000024832	7
ENSECAG00000024956	7
ENSECAG00000024979	6
ENSECAG00000025126	6
ENSECAG00000025136	8
ENSECAG00000027819	8
ENSECAG00000028160	7
ENSECAG00000028168	6
ENSECAG00000028328	7
ENSECAG00000028421	8
ENSECAG00000028845	6
ENSECAG00000028885	6
ENSECAG00000029311	6
ENSECAG00000029340	6
ENSECAG00000029414	8
ENSECAG00000029681	6
ENSECAG00000029882	8
ENSECAG00000030081	10
ENSECAG00000030129	20
ENSECAG00000030169	9
ENSECAG00000030297	9
ENSECAG00000030546	8
ENSECAG00000030648	8
ENSECAG00000030856	6
ENSECAG00000031005	8
ENSECAG00000031280	11
ENSECAG00000032353	7

Supplementary table 4. Ensembl gene IDs containing >5 LOF variants

ENSECAG00000032492	7
ENSECAG00000033152	8
ENSECAG00000033354	6
ENSECAG00000033355	8
ENSECAG00000033575	12
ENSECAG00000033604	9
ENSECAG00000033922	7
ENSECAG00000034339	20
ENSECAG00000034383	11
ENSECAG00000034395	17
ENSECAG00000034911	6
ENSECAG00000035267	8
ENSECAG00000035495	9
ENSECAG00000035510	17
ENSECAG00000035604	11
ENSECAG00000035675	6
ENSECAG00000036205	10
ENSECAG00000036352	8
ENSECAG00000037493	9
ENSECAG00000037675	7
ENSECAG00000038039	6
ENSECAG00000038257	12
ENSECAG00000038754	13
ENSECAG00000038758	8
ENSECAG00000038846	6
ENSECAG00000038976	9
ENSECAG00000040077	7
ENSECAG00000040231	7
ENSECAG00000040393	8
ENSECAG00000040529	6
ENSECAG00000040710	11
ENSECAG00000041707	13
ENSECAG00000042020	24
ENSECAG00000043572	7

Supplementary table 4. Ensembl gene IDs containing >5 LOF variants

ENSECAG00000043705	12
ENSECAG00000043734	7

Supplementary table 5. Ensembl gene IDs containing LOF variants at an allele frequency >5%

Gene ID	Number
ENSECAG00000000066	6
ENSECAG00000000162	8
ENSECAG00000000253	7
ENSECAG00000000281	11
ENSECAG00000000287	8
ENSECAG00000000291	7
ENSECAG00000000296	6
ENSECAG00000000393	7
ENSECAG00000000408	10
ENSECAG00000000505	6
ENSECAG00000000638	9
ENSECAG00000000700	6
ENSECAG00000000809	8
ENSECAG00000000873	6
ENSECAG00000000972	6
ENSECAG00000001119	8
ENSECAG00000001129	8
ENSECAG00000001358	6
ENSECAG00000001653	7
ENSECAG00000001716	9
ENSECAG00000002267	6
ENSECAG00000002578	7
ENSECAG00000002675	6
ENSECAG00000002892	9
ENSECAG00000002944	13
ENSECAG00000003079	6
ENSECAG00000003619	6
ENSECAG00000003761	8
ENSECAG00000004053	6
ENSECAG00000004409	10
ENSECAG00000004523	6
ENSECAG00000004819	6

Supplementary table 5. Ensembl gene IDs containing LOF variants at an allele frequency >5%

ENSECAG00000004906	6
ENSECAG00000004913	6
ENSECAG00000005238	14
ENSECAG00000005383	7
ENSECAG00000005417	6
ENSECAG00000005522	7
ENSECAG00000005547	7
ENSECAG00000005580	6
ENSECAG00000005608	8
ENSECAG00000005925	6
ENSECAG00000005949	7
ENSECAG00000006042	7
ENSECAG00000006457	6
ENSECAG00000006467	6
ENSECAG00000006526	8
ENSECAG00000006544	10
ENSECAG00000006557	8
ENSECAG00000006573	6
ENSECAG00000006745	6
ENSECAG00000006787	6
ENSECAG00000006885	7
ENSECAG00000006899	9
ENSECAG00000007134	6
ENSECAG00000007150	7
ENSECAG00000007192	31
ENSECAG00000007248	6
ENSECAG00000007301	7
ENSECAG00000007374	6
ENSECAG00000007442	9
ENSECAG00000007464	6
ENSECAG00000007481	6
ENSECAG00000007513	9
ENSECAG00000007644	6

**Supplementary table 5.** Ensembl gene IDs containing LOF variants at an allele frequency >5%	
ENSECAG00000007706	6
ENSECAG00000007763	7
ENSECAG00000007768	6
ENSECAG00000007955	9
ENSECAG00000007976	12
ENSECAG00000008008	6
ENSECAG00000008046	12
ENSECAG00000008051	7
ENSECAG00000008107	6
ENSECAG00000008134	6
ENSECAG00000008291	6
ENSECAG00000008458	6
ENSECAG00000008470	6
ENSECAG00000008485	6
ENSECAG00000008511	6
ENSECAG00000008535	6
ENSECAG00000008642	13
ENSECAG00000008833	6
ENSECAG00000008848	12
ENSECAG00000008893	6
ENSECAG00000008911	6
ENSECAG00000008941	6
ENSECAG00000009102	7
ENSECAG00000009111	6
ENSECAG00000009114	11
ENSECAG00000009142	9
ENSECAG00000009171	6
ENSECAG00000009218	6
ENSECAG00000009302	6
ENSECAG00000009396	12
ENSECAG00000009550	27
ENSECAG00000009583	9
ENSECAG00000009625	16

Supplementary table 5. Ensembl gene IDs containing LOF variants at an allele frequency >5%

ENSECAG00000009635	8
ENSECAG00000009721	8
ENSECAG00000009751	6
ENSECAG00000009828	6
ENSECAG00000009847	6
ENSECAG00000009856	7
ENSECAG00000009904	6
ENSECAG00000010050	6
ENSECAG00000010196	6
ENSECAG00000010201	6
ENSECAG00000010285	7
ENSECAG00000010353	6
ENSECAG00000010610	7
ENSECAG00000010685	6
ENSECAG00000010774	17
ENSECAG00000010792	6
ENSECAG00000010812	6
ENSECAG00000010919	11
ENSECAG00000010977	6
ENSECAG00000010986	7
ENSECAG00000010990	7
ENSECAG00000011055	8
ENSECAG00000011080	7
ENSECAG00000011132	7
ENSECAG00000011229	7
ENSECAG00000011451	6
ENSECAG00000011457	8
ENSECAG00000011612	8
ENSECAG00000011694	8
ENSECAG00000011718	6
ENSECAG00000011768	7
ENSECAG00000011804	70
ENSECAG00000011933	6

**Supplementary table 5.** Ensembl gene IDs containing LOF variants at an allele frequency >5%	
ENSECAG00000011995	7
ENSECAG00000012099	6
ENSECAG00000012186	6
ENSECAG00000012232	7
ENSECAG00000012335	7
ENSECAG00000012353	6
ENSECAG00000012389	6
ENSECAG00000012574	6
ENSECAG00000012816	6
ENSECAG00000012829	8
ENSECAG00000012933	8
ENSECAG00000013039	9
ENSECAG00000013053	7
ENSECAG00000013080	7
ENSECAG00000013103	9
ENSECAG00000013110	13
ENSECAG00000013124	6
ENSECAG00000013260	7
ENSECAG00000013274	6
ENSECAG00000013309	6
ENSECAG00000013366	6
ENSECAG00000013581	6
ENSECAG00000013718	6
ENSECAG00000013722	7
ENSECAG00000013759	6
ENSECAG00000013923	6
ENSECAG00000013931	6
ENSECAG00000013998	7
ENSECAG00000014002	11
ENSECAG00000014203	9
ENSECAG00000014251	7
ENSECAG00000014304	6
ENSECAG00000014377	6

Supplementary table 5. Ensembl gene IDs containing LOF variants at an allele frequency >5%

ENSECAG00000014435	8
ENSECAG00000014452	9
ENSECAG00000014531	7
ENSECAG00000014533	6
ENSECAG00000014617	6
ENSECAG00000014620	6
ENSECAG00000014700	11
ENSECAG00000014767	9
ENSECAG00000014809	7
ENSECAG00000014914	6
ENSECAG00000014967	7
ENSECAG00000014984	7
ENSECAG00000015040	9
ENSECAG00000015044	6
ENSECAG00000015086	13
ENSECAG00000015087	6
ENSECAG00000015139	9
ENSECAG00000015220	6
ENSECAG00000015258	7
ENSECAG00000015321	9
ENSECAG00000015514	8
ENSECAG00000015580	7
ENSECAG00000015843	6
ENSECAG00000015910	6
ENSECAG00000015929	7
ENSECAG00000015935	10
ENSECAG00000015938	8
ENSECAG00000015940	10
ENSECAG00000015999	7
ENSECAG00000016010	6
ENSECAG00000016082	6
ENSECAG00000016123	16
ENSECAG00000016150	9

**Supplementary table 5.** Ensembl gene IDs containing LOF variants at an allele frequency >5%	
ENSECAG00000016151	8
ENSECAG00000016212	13
ENSECAG00000016217	8
ENSECAG00000016258	8
ENSECAG00000016283	13
ENSECAG00000016340	7
ENSECAG00000016346	7
ENSECAG00000016371	11
ENSECAG00000016401	6
ENSECAG00000016447	6
ENSECAG00000016464	16
ENSECAG00000016491	6
ENSECAG00000016554	9
ENSECAG00000016707	7
ENSECAG00000016720	12
ENSECAG00000016800	8
ENSECAG00000016883	8
ENSECAG00000016912	9
ENSECAG00000016982	12
ENSECAG00000017043	6
ENSECAG00000017064	6
ENSECAG00000017143	18
ENSECAG00000017145	7
ENSECAG00000017253	6
ENSECAG00000017353	9
ENSECAG00000017376	6
ENSECAG00000017448	6
ENSECAG00000017563	13
ENSECAG00000017677	7
ENSECAG00000017711	8
ENSECAG00000017741	9
ENSECAG00000017776	6
ENSECAG00000017796	15

Supplementary table 5. Ensembl gene IDs containing LOF variants at an allele frequency >5%

ENSECAG00000017887	7
ENSECAG00000017908	6
ENSECAG00000018093	13
ENSECAG00000018233	6
ENSECAG00000018243	6
ENSECAG00000018381	6
ENSECAG00000018476	6
ENSECAG00000018485	6
ENSECAG00000018529	6
ENSECAG00000018555	9
ENSECAG00000018611	6
ENSECAG00000018775	7
ENSECAG00000018948	9
ENSECAG00000019044	6
ENSECAG00000019125	8
ENSECAG00000019162	7
ENSECAG00000019207	10
ENSECAG00000019242	8
ENSECAG00000019326	7
ENSECAG00000019545	7
ENSECAG00000019590	8
ENSECAG00000019675	11
ENSECAG00000019713	6
ENSECAG00000019776	9
ENSECAG00000019916	6
ENSECAG00000019932	13
ENSECAG00000020091	7
ENSECAG00000020168	9
ENSECAG00000020175	7
ENSECAG00000020181	7
ENSECAG00000020298	6
ENSECAG00000020358	12
ENSECAG00000020433	7

Supplementary table 5. Ensembl gene IDs containing LOF variants at an allele frequency >5%

ENSECAG00000020447	6
ENSECAG00000020463	6
ENSECAG00000020506	9
ENSECAG00000020645	7
ENSECAG00000020679	7
ENSECAG00000020699	8
ENSECAG00000020764	6
ENSECAG00000020792	6
ENSECAG00000020833	7
ENSECAG00000020885	13
ENSECAG00000020984	11
ENSECAG00000021014	18
ENSECAG00000021031	7
ENSECAG00000021057	6
ENSECAG00000021147	7
ENSECAG00000021171	6
ENSECAG00000021225	6
ENSECAG00000021252	6
ENSECAG00000021275	7
ENSECAG00000021300	11
ENSECAG00000021318	15
ENSECAG00000021325	7
ENSECAG00000021385	8
ENSECAG00000021443	7
ENSECAG00000021561	9
ENSECAG00000021657	8
ENSECAG00000021698	6
ENSECAG00000021742	10
ENSECAG00000021813	8
ENSECAG00000021818	6
ENSECAG00000021840	6
ENSECAG00000021868	14
ENSECAG00000021880	6

**Supplementary table 5.** Ensembl gene IDs containing LOF variants at an allele frequency >5%	
ENSECAG00000021898	6
ENSECAG00000021997	7
ENSECAG00000022005	12
ENSECAG00000022072	10
ENSECAG00000022093	7
ENSECAG00000022120	6
ENSECAG00000022262	6
ENSECAG00000022272	8
ENSECAG00000022377	27
ENSECAG00000022405	11
ENSECAG00000022440	10
ENSECAG00000022453	7
ENSECAG00000022500	9
ENSECAG00000022544	9
ENSECAG00000022546	10
ENSECAG00000022635	6
ENSECAG00000022671	11
ENSECAG00000022820	18
ENSECAG00000022875	6
ENSECAG00000022941	8
ENSECAG00000022955	8
ENSECAG00000022984	7
ENSECAG00000023009	6
ENSECAG00000023016	6
ENSECAG00000023034	8
ENSECAG00000023061	7
ENSECAG00000023086	7
ENSECAG00000023231	6
ENSECAG00000023311	6
ENSECAG00000023455	8
ENSECAG00000023501	12
ENSECAG00000023594	6
ENSECAG00000023800	6

Supplementary table 5. Ensembl gene IDs containing LOF variants at an allele frequency >5%

ENSECAG00000023849	6
ENSECAG00000023879	7
ENSECAG00000023942	6
ENSECAG00000024082	8
ENSECAG00000024091	6
ENSECAG00000024149	12
ENSECAG00000024243	8
ENSECAG00000024259	21
ENSECAG00000024267	6
ENSECAG00000024343	7
ENSECAG00000024438	6
ENSECAG00000024461	6
ENSECAG00000024518	6
ENSECAG00000024558	8
ENSECAG00000024623	8
ENSECAG00000024654	7
ENSECAG00000024832	7
ENSECAG00000024956	7
ENSECAG00000024979	6
ENSECAG00000025126	6
ENSECAG00000025136	8
ENSECAG00000027819	8
ENSECAG00000028160	7
ENSECAG00000028168	6
ENSECAG00000028328	7
ENSECAG00000028421	8
ENSECAG00000028845	6
ENSECAG00000028885	6
ENSECAG00000029311	6
ENSECAG00000029340	6
ENSECAG00000029414	8
ENSECAG00000029681	6
ENSECAG00000029882	8

Supplementary table 5. Ensembl gene IDs containing LOF variants at an allele frequency >5%

ENSECAG00000030081	10
ENSECAG00000030129	20
ENSECAG00000030169	9
ENSECAG00000030297	9
ENSECAG00000030546	8
ENSECAG00000030648	8
ENSECAG00000030856	6
ENSECAG00000031005	8
ENSECAG00000031280	11
ENSECAG00000032353	7
ENSECAG00000032492	7
ENSECAG00000033152	8
ENSECAG00000033354	6
ENSECAG00000033355	8
ENSECAG00000033575	12
ENSECAG00000033604	9
ENSECAG00000033922	7
ENSECAG00000034339	20
ENSECAG00000034383	11
ENSECAG00000034395	17
ENSECAG00000034911	6
ENSECAG00000035267	8
ENSECAG00000035495	9
ENSECAG00000035510	17
ENSECAG00000035604	11
ENSECAG00000035675	6
ENSECAG00000036205	10
ENSECAG00000036352	8
ENSECAG00000037493	9
ENSECAG00000037675	7
ENSECAG00000038039	6
ENSECAG00000038257	12
ENSECAG00000038754	13

Supplementary table 5. Ensembl gene IDs containing LOF variants at an allele frequency >5%

ENSECAG00000038758	8
ENSECAG00000038846	6
ENSECAG00000038976	9
ENSECAG00000040077	7
ENSECAG00000040231	7
ENSECAG00000040393	8
ENSECAG00000040529	6
ENSECAG00000040710	11
ENSECAG00000041707	13
ENSECAG00000042020	24
ENSECAG00000043572	7
ENSECAG00000043705	12
ENSECAG00000043734	7

**Supplementary table 6.** Breed specific allele frequencies for the known variants									
**Phenotype**	**Phenotype abbreviation**	**Breed**	**Allele frequency**	**Gene name**	**Deleterious**	**Causative**	**Reference allele**	**Alternate allele**	**chromosome:position**
Androgen insensitivity syndrome	AIS_QH	NA	NA	AR	yes	yes	A	G	chrX:52728703
Androgen insensitivity syndrome	AIS_TB1	NA	NA	AR	yes	yes	G	C	chrX:52872393
Androgen insensitivity syndrome	AIS_TB2	NA	NA	AR	yes	yes	C	T	chrX:52878093
Androgen insensitivity syndrome	AIS_TWH	NA	NA	AR	yes	yes	T	*	chrX:52728885
Bone fracture	BF1	WP	0.125	MSTN	yes	no	C	T	chr18:65924323
Bone fracture	BF1	FM	0.267	MSTN	yes	no	C	T	chr18:65924323
Bone fracture	BF1	Shetland	0.427	MSTN	yes	no	C	T	chr18:65924323
Bone fracture	BF1	STB	0.068	MSTN	yes	no	C	T	chr18:65924323
Bone fracture	BF1	QH	0.670	MSTN	yes	no	C	T	chr18:65924323
Bone fracture	BF1	TB	0.513	MSTN	yes	no	C	T	chr18:65924323
Bone fracture	BF1	Other	0.159	MSTN	yes	no	C	T	chr18:65924323
Bone fracture	BF1	Icelandic	0.294	MSTN	yes	no	C	T	chr18:65924323
Bone fracture	BF1	Warmblood	0.086	MSTN	yes	no	C	T	chr18:65924323
Bone fracture	BF1	Belgian	0.250	MSTN	yes	no	C	T	chr18:65924323
Bone fracture	BF1	Arabian	0.145	MSTN	yes	no	C	T	chr18:65924323
Bone fracture	BF1	Morgan	0.091	MSTN	yes	no	C	T	chr18:65924323
Bone fracture	BF1	Clydesdale	0.184	MSTN	yes	no	C	T	chr18:65924323
Bone fracture	BF2	Icelandic	0.088	MSTN	yes	no	T	G	chr18:65983696
Bone fracture	BF2	Clydesdale	0.447	MSTN	yes	no	T	G	chr18:65983696
Bone fracture	BF2	WP	0.400	MSTN	yes	no	T	G	chr18:65983696
Bone fracture	BF2	TB	0.553	MSTN	yes	no	T	G	chr18:65983696
Bone fracture	BF2	STB	0.017	MSTN	yes	no	T	G	chr18:65983696
Bone fracture	BF2	Morgan	0.091	MSTN	yes	no	T	G	chr18:65983696
Bone fracture	BF2	Warmblood	0.086	MSTN	yes	no	T	G	chr18:65983696
Bone fracture	BF2	Shetland	0.155	MSTN	yes	no	T	G	chr18:65983696
Bone fracture	BF2	Arabian	0.158	MSTN	yes	no	T	G	chr18:65983696
Bone fracture	BF2	Other	0.109	MSTN	yes	no	T	G	chr18:65983696
Bone fracture	BF2	QH	0.741	MSTN	yes	no	T	G	chr18:65983696
Bone fracture	BF2	Belgian	0.175	MSTN	yes	no	T	G	chr18:65983696
Bone fracture	BF2	FM	0.233	MSTN	yes	no	T	G	chr18:65983696

NA: Variants not present in this population

Supplementary table 6. Breed specific allele frequencies for the known variants

Bone fracture	BF3	STB	0.017	MSTN	yes	no	T	C	chr18:66608679
Bone fracture	BF3	Belgian	0.175	MSTN	yes	no	T	C	chr18:66608679
Bone fracture	BF3	Arabian	0.013	MSTN	yes	no	T	C	chr18:66608679
Bone fracture	BF3	Clydesdale	0.158	MSTN	yes	no	T	C	chr18:66608679
Bone fracture	BF3	FM	0.167	MSTN	yes	no	T	C	chr18:66608679
Bone fracture	BF3	Morgan	0.114	MSTN	yes	no	T	C	chr18:66608679
Bone fracture	BF3	Other	0.168	MSTN	yes	no	T	C	chr18:66608679
Bone fracture	BF3	Icelandic	0.088	MSTN	yes	no	T	C	chr18:66608679
Bone fracture	BF3	QH	0.792	MSTN	yes	no	T	C	chr18:66608679
Bone fracture	BF3	Warmblood	0.157	MSTN	yes	no	T	C	chr18:66608679
Bone fracture	BF3	TB	0.546	MSTN	yes	no	T	C	chr18:66608679
Bone fracture	BF3	Shetland	0.218	MSTN	yes	no	T	C	chr18:66608679
Bone fracture	BF3	WP	0.175	MSTN	yes	no	T	C	chr18:66608679
Cerebellar abiotrophy	CA	Belgian	0.000	MUTYH	yes	yes	C	T	chr2:13122415
Cerebellar abiotrophy	CA	Clydesdale	0.000	MUTYH	yes	yes	C	T	chr2:13122415
Cerebellar abiotrophy	CA	QH	0.005	MUTYH	yes	yes	C	T	chr2:13122415
Cerebellar abiotrophy	CA	Arabian	0.105	MUTYH	yes	yes	C	T	chr2:13122415
Cerebellar abiotrophy	CA	STB	0.000	MUTYH	yes	yes	C	T	chr2:13122415
Cerebellar abiotrophy	CA	FM	0.000	MUTYH	yes	yes	C	T	chr2:13122415
Cerebellar abiotrophy	CA	Other	0.000	MUTYH	yes	yes	C	T	chr2:13122415
Cerebellar abiotrophy	CA	Icelandic	0.000	MUTYH	yes	yes	C	T	chr2:13122415
Cerebellar abiotrophy	CA	WP	0.000	MUTYH	yes	yes	C	T	chr2:13122415
Cerebellar abiotrophy	CA	Morgan	0.000	MUTYH	yes	yes	C	T	chr2:13122415
Cerebellar abiotrophy	CA	TB	0.000	MUTYH	yes	yes	C	T	chr2:13122415
Cerebellar abiotrophy	CA	Warmblood	0.000	MUTYH	yes	yes	C	T	chr2:13122415
Cerebellar abiotrophy	CA	Shetland	0.000	MUTYH	yes	yes	C	T	chr2:13122415
Curly coat	CC1	Warmblood	0.000	KRT25	yes	yes	G	A	chr11:21962991
Curly coat	CC1	Clydesdale	0.000	KRT25	yes	yes	G	A	chr11:21962991
Curly coat	CC1	Arabian	0.000	KRT25	yes	yes	G	A	chr11:21962991
Curly coat	CC1	FM	0.000	KRT25	yes	yes	G	A	chr11:21962991
Curly coat	CC1	TB	0.000	KRT25	yes	yes	G	A	chr11:21962991
Curly coat	CC1	Morgan	0.000	KRT25	yes	yes	G	A	chr11:21962991
Curly coat	CC1	Belgian	0.000	KRT25	yes	yes	G	A	chr11:21962991
Curly coat	CC1	Shetland	0.000	KRT25	yes	yes	G	A	chr11:21962991

NA: Variants not present in this population

Supplementary table 6. Breed specific allele frequencies for the known variants

Curly coat	CC1	WP	0.000	KRT25	yes	yes	G	A	chr11:21962991
Curly coat	CC1	Other	0.005	KRT25	yes	yes	G	A	chr11:21962991
Curly coat	CC1	Icelandic	0.000	KRT25	yes	yes	G	A	chr11:21962991
Curly coat	CC1	STB	0.000	KRT25	yes	yes	G	A	chr11:21962991
Curly coat	CC1	QH	0.000	KRT25	yes	yes	G	A	chr11:21962991
Curly coat with hypotrichosis	CC2	NA	NA	SP6	yes	yes	C	T	chr11:24096309
Congenital liver fibrosis	CLF1	Arabian	0.013	PKHD1	yes	no	G	T	chr20:50612743
Congenital liver fibrosis	CLF1	Warmblood	0.157	PKHD1	yes	no	G	T	chr20:50612743
Congenital liver fibrosis	CLF1	Belgian	0.000	PKHD1	yes	no	G	T	chr20:50612743
Congenital liver fibrosis	CLF1	Clydesdale	0.289	PKHD1	yes	no	G	T	chr20:50612743
Congenital liver fibrosis	CLF1	FM	0.050	PKHD1	yes	no	G	T	chr20:50612743
Congenital liver fibrosis	CLF1	Shetland	0.000	PKHD1	yes	no	G	T	chr20:50612743
Congenital liver fibrosis	CLF1	Icelandic	0.029	PKHD1	yes	no	G	T	chr20:50612743
Congenital liver fibrosis	CLF1	Morgan	0.045	PKHD1	yes	no	G	T	chr20:50612743
Congenital liver fibrosis	CLF1	Other	0.132	PKHD1	yes	no	G	T	chr20:50612743
Congenital liver fibrosis	CLF1	QH	0.094	PKHD1	yes	no	G	T	chr20:50612743
Congenital liver fibrosis	CLF1	TB	0.237	PKHD1	yes	no	G	T	chr20:50612743
Congenital liver fibrosis	CLF1	STB	0.017	PKHD1	yes	no	G	T	chr20:50612743
Congenital liver fibrosis	CLF1	WP	0.225	PKHD1	yes	no	G	T	chr20:50612743
Congenital liver fibrosis	CLF2	QH	0.047	PKHD1	yes	no	A	T	chr20:50612810
Congenital liver fibrosis	CLF2	Belgian	0.075	PKHD1	yes	no	A	T	chr20:50612810
Congenital liver fibrosis	CLF2	Other	0.064	PKHD1	yes	no	A	T	chr20:50612810
Congenital liver fibrosis	CLF2	Clydesdale	0.158	PKHD1	yes	no	A	T	chr20:50612810
Congenital liver fibrosis	CLF2	FM	0.067	PKHD1	yes	no	A	T	chr20:50612810
Congenital liver fibrosis	CLF2	Arabian	0.079	PKHD1	yes	no	A	T	chr20:50612810
Congenital liver fibrosis	CLF2	Morgan	0.000	PKHD1	yes	no	A	T	chr20:50612810

NA: Variants not present in this population

Supplementary table 6. Breed specific allele frequencies for the known variants

Congenital liver fibrosis	CLF2	Shetland	0.009	PKHD1	yes	no	A	T	chr20:50612810
Congenital liver fibrosis	CLF2	TB	0.013	PKHD1	yes	no	A	T	chr20:50612810
Congenital liver fibrosis	CLF2	Icelandic	0.029	PKHD1	yes	no	A	T	chr20:50612810
Congenital liver fibrosis	CLF2	STB	0.034	PKHD1	yes	no	A	T	chr20:50612810
Congenital liver fibrosis	CLF2	WP	0.100	PKHD1	yes	no	A	T	chr20:50612810
Congenital liver fibrosis	CLF2	Warmblood	0.143	PKHD1	yes	no	A	T	chr20:50612810
Congenital liver fibrosis	CLF3	Arabian	0.013	PKHD1	yes	no	A	G	chr20:50612870
Congenital liver fibrosis	CLF3	Icelandic	0.029	PKHD1	yes	no	A	G	chr20:50612870
Congenital liver fibrosis	CLF3	QH	0.090	PKHD1	yes	no	A	G	chr20:50612870
Congenital liver fibrosis	CLF3	Clydesdale	0.263	PKHD1	yes	no	A	G	chr20:50612870
Congenital liver fibrosis	CLF3	Belgian	0.000	PKHD1	yes	no	A	G	chr20:50612870
Congenital liver fibrosis	CLF3	WP	0.175	PKHD1	yes	no	A	G	chr20:50612870
Congenital liver fibrosis	CLF3	Other	0.123	PKHD1	yes	no	A	G	chr20:50612870
Congenital liver fibrosis	CLF3	TB	0.217	PKHD1	yes	no	A	G	chr20:50612870
Congenital liver fibrosis	CLF3	FM	0.050	PKHD1	yes	no	A	G	chr20:50612870
Congenital liver fibrosis	CLF3	Morgan	0.000	PKHD1	yes	no	A	G	chr20:50612870
Congenital liver fibrosis	CLF3	Warmblood	0.157	PKHD1	yes	no	A	G	chr20:50612870
Congenital liver fibrosis	CLF3	STB	0.017	PKHD1	yes	no	A	G	chr20:50612870
Congenital liver fibrosis	CLF3	Shetland	0.000	PKHD1	yes	no	A	G	chr20:50612870
Congenital liver fibrosis	CLF4	Arabian	0.079	PKHD1	yes	no	G	A	chr20:50645852
Congenital liver fibrosis	CLF4	Icelandic	0.029	PKHD1	yes	no	G	A	chr20:50645852
Congenital liver fibrosis	CLF4	Other	0.086	PKHD1	yes	no	G	A	chr20:50645852
Congenital liver fibrosis	CLF4	Belgian	0.150	PKHD1	yes	no	G	A	chr20:50645852
Congenital liver fibrosis	CLF4	FM	0.067	PKHD1	yes	no	G	A	chr20:50645852
Congenital liver	CLF4	STB	0.025	PKHD1	yes	no	G	A	chr20:50645852

NA: Variants not present in this population

Supplementary table 6. Breed specific allele frequencies for the known variants

fibrosis									
Congenital liver fibrosis	CLF4	WP	0.100	PKHD1	yes	no	G	A	chr20:50645852
Congenital liver fibrosis	CLF4	Clydesdale	0.237	PKHD1	yes	no	G	A	chr20:50645852
Congenital liver fibrosis	CLF4	Morgan	0.000	PKHD1	yes	no	G	A	chr20:50645852
Congenital liver fibrosis	CLF4	TB	0.020	PKHD1	yes	no	G	A	chr20:50645852
Congenital liver fibrosis	CLF4	Warmblood	0.114	PKHD1	yes	no	G	A	chr20:50645852
Congenital liver fibrosis	CLF4	QH	0.052	PKHD1	yes	no	G	A	chr20:50645852
Congenital liver fibrosis	CLF4	Shetland	0.009	PKHD1	yes	no	G	A	chr20:50645852
Congenital liver fibrosis	CLF5	Clydesdale	0.895	PKHD1	yes	no	T	C	chr20:50645969
Congenital liver fibrosis	CLF5	Belgian	0.425	PKHD1	yes	no	T	C	chr20:50645969
Congenital liver fibrosis	CLF5	QH	0.264	PKHD1	yes	no	T	C	chr20:50645969
Congenital liver fibrosis	CLF5	Arabian	0.250	PKHD1	yes	no	T	C	chr20:50645969
Congenital liver fibrosis	CLF5	FM	0.483	PKHD1	yes	no	T	C	chr20:50645969
Congenital liver fibrosis	CLF5	Icelandic	0.324	PKHD1	yes	no	T	C	chr20:50645969
Congenital liver fibrosis	CLF5	Other	0.405	PKHD1	yes	no	T	C	chr20:50645969
Congenital liver fibrosis	CLF5	TB	0.408	PKHD1	yes	no	T	C	chr20:50645969
Congenital liver fibrosis	CLF5	Shetland	0.618	PKHD1	yes	no	T	C	chr20:50645969
Congenital liver fibrosis	CLF5	Morgan	0.227	PKHD1	yes	no	T	C	chr20:50645969
Congenital liver fibrosis	CLF5	Warmblood	0.414	PKHD1	yes	no	T	C	chr20:50645969
Congenital liver fibrosis	CLF5	STB	0.297	PKHD1	yes	no	T	C	chr20:50645969
Congenital liver fibrosis	CLF5	WP	0.500	PKHD1	yes	no	T	C	chr20:50645969
Coat colour, agouti	coat_bl	FM	0.150	ASIP	CC	CC	CAGCAGAA	C	chr22:26067462
Coat colour, agouti	coat_bl	Clydesdale	0.211	ASIP	CC	CC	CAGCAGAA	C	chr22:26067462
Coat colour, agouti	coat_bl	Belgian	0.575	ASIP	CC	CC	CAGCAGAA	C	chr22:26067462
Coat colour, agouti	coat_bl	Other	0.336	ASIP	CC	CC	CAGCAGAA	C	chr22:26067462
Coat colour, agouti	coat_bl	QH	0.325	ASIP	CC	CC	CAGCAGAA	C	chr22:26067462
Coat colour, agouti	coat_bl	Shetland	0.509	ASIP	CC	CC	CAGCAGAA	C	chr22:26067462

NA: Variants not present in this population

Supplementary table 6. Breed specific allele frequencies for the known variants

Coat colour, agouti	coat_bl	Icelandic	0.706	ASIP	CC	CC	CAGCAGAA	C	chr22:26067462
Coat colour, agouti	coat_bl	Arabian	0.145	ASIP	CC	CC	CAGCAGAA	C	chr22:26067462
Coat colour, agouti	coat_bl	Morgan	0.364	ASIP	CC	CC	CAGCAGAA	C	chr22:26067462
Coat colour, agouti	coat_bl	STB	0.254	ASIP	CC	CC	CAGCAGAA	C	chr22:26067462
Coat colour, agouti	coat_bl	TB	0.191	ASIP	CC	CC	CAGCAGAA	C	chr22:26067462
Coat colour, agouti	coat_bl	Warmblood	0.443	ASIP	CC	CC	CAGCAGAA	C	chr22:26067462
Coat colour, agouti	coat_bl	WP	0.375	ASIP	CC	CC	CAGCAGAA	C	chr22:26067462
Brindle 1	coat_bri	Icelandic	0.000	MBTPS2	CC	CC	T	C	chrX:17286855
Brindle 1	coat_bri	QH	0.000	MBTPS2	CC	CC	T	C	chrX:17286855
Brindle 1	coat_bri	FM	0.000	MBTPS2	CC	CC	T	C	chrX:17286855
Brindle 1	coat_bri	Morgan	0.000	MBTPS2	CC	CC	T	C	chrX:17286855
Brindle 1	coat_bri	Other	0.000	MBTPS2	CC	CC	T	C	chrX:17286855
Brindle 1	coat_bri	TB	0.000	MBTPS2	CC	CC	T	C	chrX:17286855
Brindle 1	coat_bri	Shetland	0.000	MBTPS2	CC	CC	T	C	chrX:17286855
Brindle 1	coat_bri	Belgian	0.000	MBTPS2	CC	CC	T	C	chrX:17286855
Brindle 1	coat_bri	Warmblood	0.014	MBTPS2	CC	CC	T	C	chrX:17286855
Brindle 1	coat_bri	STB	0.000	MBTPS2	CC	CC	T	C	chrX:17286855
Brindle 1	coat_bri	WP	0.000	MBTPS2	CC	CC	T	C	chrX:17286855
Brindle 1	coat_bri	Clydesdale	0.000	MBTPS2	CC	CC	T	C	chrX:17286855
Brindle 1	coat_bri	Arabian	0.000	MBTPS2	CC	CC	T	C	chrX:17286855
Coat colour, champagne	coat_champ	NA	NA	SLC36A1	CC	CC	A	G	chr14:70997
Coat colour, extension	coat_chest1	Other	0.418	MC1R	CC	CC	C	T	chr3:36979560
Coat colour, extension	coat_chest1	Icelandic	0.588	MC1R	CC	CC	C	T	chr3:36979560
Coat colour, extension	coat_chest1	Shetland	0.391	MC1R	CC	CC	C	T	chr3:36979560
Coat colour, extension	coat_chest1	Morgan	0.386	MC1R	CC	CC	C	T	chr3:36979560
Coat colour, extension	coat_chest1	QH	0.726	MC1R	CC	CC	C	T	chr3:36979560
Coat colour, extension	coat_chest1	Belgian	0.900	MC1R	CC	CC	C	T	chr3:36979560
Coat colour, extension	coat_chest1	TB	0.441	MC1R	CC	CC	C	T	chr3:36979560
Coat colour, extension	coat_chest1	Warmblood	0.443	MC1R	CC	CC	C	T	chr3:36979560
Coat colour, extension	coat_chest1	WP	0.675	MC1R	CC	CC	C	T	chr3:36979560
Coat colour, extension	coat_chest1	Clydesdale	0.079	MC1R	CC	CC	C	T	chr3:36979560
Coat colour, extension	coat_chest1	FM	0.533	MC1R	CC	CC	C	T	chr3:36979560
Coat colour, extension	coat_chest1	STB	0.153	MC1R	CC	CC	C	T	chr3:36979560

NA: Variants not present in this population

Supplementary table 6. Breed specific allele frequencies for the known variants

Coat colour, extension	coat_chest1	Arabian	0.750	MC1R	CC	CC	C	T	chr3:36979560
Coat colour, extension	coat_chest2	Other	0.014	MC1R	CC	CC	G	A	chr3:36979562
Coat colour, extension	coat_chest2	STB	0.000	MC1R	CC	CC	G	A	chr3:36979562
Coat colour, extension	coat_chest2	QH	0.000	MC1R	CC	CC	G	A	chr3:36979562
Coat colour, extension	coat_chest2	WP	0.000	MC1R	CC	CC	G	A	chr3:36979562
Coat colour, extension	coat_chest2	Warmblood	0.000	MC1R	CC	CC	G	A	chr3:36979562
Coat colour, extension	coat_chest2	Icelandic	0.000	MC1R	CC	CC	G	A	chr3:36979562
Coat colour, extension	coat_chest2	TB	0.000	MC1R	CC	CC	G	A	chr3:36979562
Coat colour, extension	coat_chest2	Belgian	0.150	MC1R	CC	CC	G	A	chr3:36979562
Coat colour, extension	coat_chest2	FM	0.000	MC1R	CC	CC	G	A	chr3:36979562
Coat colour, extension	coat_chest2	Morgan	0.000	MC1R	CC	CC	G	A	chr3:36979562
Coat colour, extension	coat_chest2	Shetland	0.000	MC1R	CC	CC	G	A	chr3:36979562
Coat colour, extension	coat_chest2	Arabian	0.000	MC1R	CC	CC	G	A	chr3:36979562
Coat colour, extension	coat_chest2	Clydesdale	0.000	MC1R	CC	CC	G	A	chr3:36979562
Coat colour, cream dilution	coat_cream	STB	0.000	SLC45A2	CC	CC	G	A	chr21:31690653
Coat colour, cream dilution	coat_cream	FM	0.000	SLC45A2	CC	CC	G	A	chr21:31690653
Coat colour, cream dilution	coat_cream	Icelandic	0.000	SLC45A2	CC	CC	G	A	chr21:31690653
Coat colour, cream dilution	coat_cream	QH	0.071	SLC45A2	CC	CC	G	A	chr21:31690653
Coat colour, cream dilution	coat_cream	Shetland	0.000	SLC45A2	CC	CC	G	A	chr21:31690653
Coat colour, cream dilution	coat_cream	Arabian	0.000	SLC45A2	CC	CC	G	A	chr21:31690653
Coat colour, cream dilution	coat_cream	Morgan	0.068	SLC45A2	CC	CC	G	A	chr21:31690653
Coat colour, cream dilution	coat_cream	TB	0.000	SLC45A2	CC	CC	G	A	chr21:31690653
Coat colour, cream dilution	coat_cream	Warmblood	0.000	SLC45A2	CC	CC	G	A	chr21:31690653
Coat colour, cream dilution	coat_cream	Clydesdale	0.000	SLC45A2	CC	CC	G	A	chr21:31690653
Coat colour, cream dilution	coat_cream	Other	0.009	SLC45A2	CC	CC	G	A	chr21:31690653
Coat colour, cream dilution	coat_cream	WP	0.050	SLC45A2	CC	CC	G	A	chr21:31690653
Coat colour, cream dilution	coat_cream	Belgian	0.000	SLC45A2	CC	CC	G	A	chr21:31690653
Coat colour,white spotting	coat_macc	NA	NA	MITF	CC	CC	A	G	chr16:21564980

NA: Variants not present in this population

Supplementary table 6. Breed specific allele frequencies for the known variants

Coat colour, phaeomelanin dilution, MFSD12-related	coat_mush	NA	NA	MFSD12	CC	CC	C	CC	chr7:2544510
Coat colour, dun	coat_ND1	TB	0.000	TBX3	CC	CC	A	G	chr8:20665434
Coat colour, dun	coat_ND1	WP	0.000	TBX3	CC	CC	A	G	chr8:20665434
Coat colour, dun	coat_ND1	Warmblood	0.014	TBX3	CC	CC	A	G	chr8:20665434
Coat colour, dun	coat_ND1	STB	0.000	TBX3	CC	CC	A	G	chr8:20665434
Coat colour, dun	coat_ND1	QH	0.028	TBX3	CC	CC	A	G	chr8:20665434
Coat colour, dun	coat_ND1	Other	0.050	TBX3	CC	CC	A	G	chr8:20665434
Coat colour, dun	coat_ND1	Morgan	0.000	TBX3	CC	CC	A	G	chr8:20665434
Coat colour, dun	coat_ND1	Shetland	0.000	TBX3	CC	CC	A	G	chr8:20665434
Coat colour, dun	coat_ND1	Icelandic	0.029	TBX3	CC	CC	A	G	chr8:20665434
Coat colour, dun	coat_ND1	FM	0.000	TBX3	CC	CC	A	G	chr8:20665434
Coat colour, dun	coat_ND1	Arabian	0.000	TBX3	CC	CC	A	G	chr8:20665434
Coat colour, dun	coat_ND1	Clydesdale	0.000	TBX3	CC	CC	A	G	chr8:20665434
Coat colour, dun	coat_ND1	Belgian	0.000	TBX3	CC	CC	A	G	chr8:20665434
Coat colour, dun	coat_ND2	Shetland	0.027	TBX3	CC	CC	A	AGCCTTCT	chr8:20665794
Coat colour, dun	coat_ND2	Warmblood	0.029	TBX3	CC	CC	A	AGCCTTCT	chr8:20665794
Coat colour, dun	coat_ND2	Other	0.114	TBX3	CC	CC	A	AGCCTTCT	chr8:20665794
Coat colour, dun	coat_ND2	TB	0.013	TBX3	CC	CC	A	AGCCTTCT	chr8:20665794
Coat colour, dun	coat_ND2	QH	0.094	TBX3	CC	CC	A	AGCCTTCT	chr8:20665794
Coat colour, dun	coat_ND2	WP	0.000	TBX3	CC	CC	A	AGCCTTCT	chr8:20665794
Coat colour, dun	coat_ND2	Belgian	0.000	TBX3	CC	CC	A	AGCCTTCT	chr8:20665794
Coat colour, dun	coat_ND2	STB	0.008	TBX3	CC	CC	A	AGCCTTCT	chr8:20665794
Coat colour, dun	coat_ND2	Morgan	0.045	TBX3	CC	CC	A	AGCCTTCT	chr8:20665794
Coat colour, dun	coat_ND2	Icelandic	0.059	TBX3	CC	CC	A	AGCCTTCT	chr8:20665794
Coat colour, dun	coat_ND2	Arabian	0.382	TBX3	CC	CC	A	AGCCTTCT	chr8:20665794
Coat colour, dun	coat_ND2	FM	0.017	TBX3	CC	CC	A	AGCCTTCT	chr8:20665794
Coat colour, dun	coat_ND2	Clydesdale	0.000	TBX3	CC	CC	A	AGCCTTCT	chr8:20665794
Coat colour, cream dilution	coat_pearl	NA	NA	SLC45A2	CC	CC	G	A	chr21:31709690
Coat colour,roan	coat_roan1	QH	0.024	KIT	CC	CC	A	G	chr3:79543439
Coat colour,roan	coat_roan1	Shetland	0.000	KIT	CC	CC	A	G	chr3:79543439
Coat colour,roan	coat_roan1	STB	0.000	KIT	CC	CC	A	G	chr3:79543439
Coat colour,roan	coat_roan1	TB	0.000	KIT	CC	CC	A	G	chr3:79543439
Coat colour,roan	coat_roan1	FM	0.000	KIT	CC	CC	A	G	chr3:79543439

NA: Variants not present in this population

Supplementary table 6. Breed specific allele frequencies for the known variants

Coat colour,roan	coat_roan1	Icelandic	0.000	KIT	CC	CC	A	G	chr3:79543439
Coat colour,roan	coat_roan1	WP	0.075	KIT	CC	CC	A	G	chr3:79543439
Coat colour,roan	coat_roan1	Belgian	0.000	KIT	CC	CC	A	G	chr3:79543439
Coat colour,roan	coat_roan1	Arabian	0.000	KIT	CC	CC	A	G	chr3:79543439
Coat colour,roan	coat_roan1	Warmblood	0.000	KIT	CC	CC	A	G	chr3:79543439
Coat colour,roan	coat_roan1	Other	0.023	KIT	CC	CC	A	G	chr3:79543439
Coat colour,roan	coat_roan1	Morgan	0.023	KIT	CC	CC	A	G	chr3:79543439
Coat colour,roan	coat_roan1	Clydesdale	0.000	KIT	CC	CC	A	G	chr3:79543439
Coat colour,roan	coat_roan2	NA	NA	KIT	CC	CC	A	C	chr3:79548356
Coat colour,roan	coat_roan3	TB	0.079	KIT	CC	CC	C	G	chr3:79545073
Coat colour,roan	coat_roan3	WP	0.050	KIT	CC	CC	C	G	chr3:79545073
Coat colour,roan	coat_roan3	Other	0.082	KIT	CC	CC	C	G	chr3:79545073
Coat colour,roan	coat_roan3	Icelandic	0.235	KIT	CC	CC	C	G	chr3:79545073
Coat colour,roan	coat_roan3	STB	0.000	KIT	CC	CC	C	G	chr3:79545073
Coat colour,roan	coat_roan3	Morgan	0.023	KIT	CC	CC	C	G	chr3:79545073
Coat colour,roan	coat_roan3	QH	0.038	KIT	CC	CC	C	G	chr3:79545073
Coat colour,roan	coat_roan3	Belgian	0.050	KIT	CC	CC	C	G	chr3:79545073
Coat colour,roan	coat_roan3	Warmblood	0.014	KIT	CC	CC	C	G	chr3:79545073
Coat colour,roan	coat_roan3	Arabian	0.039	KIT	CC	CC	C	G	chr3:79545073
Coat colour,roan	coat_roan3	Clydesdale	0.000	KIT	CC	CC	C	G	chr3:79545073
Coat colour,roan	coat_roan3	Shetland	0.109	KIT	CC	CC	C	G	chr3:79545073
Coat colour,roan	coat_roan3	FM	0.150	KIT	CC	CC	C	G	chr3:79545073
Coat colour,roan	coat_roan4	WP	0.050	KIT	CC	CC	T	A	chr3:79544372
Coat colour,roan	coat_roan4	Icelandic	0.206	KIT	CC	CC	T	A	chr3:79544372
Coat colour,roan	coat_roan4	Other	0.105	KIT	CC	CC	T	A	chr3:79544372
Coat colour,roan	coat_roan4	Shetland	0.127	KIT	CC	CC	T	A	chr3:79544372
Coat colour,roan	coat_roan4	Belgian	0.050	KIT	CC	CC	T	A	chr3:79544372
Coat colour,roan	coat_roan4	FM	0.167	KIT	CC	CC	T	A	chr3:79544372
Coat colour,roan	coat_roan4	Morgan	0.023	KIT	CC	CC	T	A	chr3:79544372
Coat colour,roan	coat_roan4	Warmblood	0.014	KIT	CC	CC	T	A	chr3:79544372
Coat colour,roan	coat_roan4	Arabian	0.039	KIT	CC	CC	T	A	chr3:79544372
Coat colour,roan	coat_roan4	QH	0.038	KIT	CC	CC	T	A	chr3:79544372
Coat colour,roan	coat_roan4	TB	0.079	KIT	CC	CC	T	A	chr3:79544372
Coat colour,roan	coat_roan4	Clydesdale	0.000	KIT	CC	CC	T	A	chr3:79544372

NA: Variants not present in this population

Supplementary table 6. Breed specific allele frequencies for the known variants

Coat colour,roan	coat_roan4	STB	0.008	KIT	CC	CC	T	A	chr3:79544372
Coat colour,roan	coat_roan5	NA	NA	KIT	CC	CC	C	A	chr3:79544334
Coat colour,roan	coat_roan6	QH	0.094	KIT	CC	CC	T	C	chr3:79540110
Coat colour,roan	coat_roan6	FM	0.183	KIT	CC	CC	T	C	chr3:79540110
Coat colour,roan	coat_roan6	TB	0.066	KIT	CC	CC	T	C	chr3:79540110
Coat colour,roan	coat_roan6	Belgian	0.250	KIT	CC	CC	T	C	chr3:79540110
Coat colour,roan	coat_roan6	STB	0.025	KIT	CC	CC	T	C	chr3:79540110
Coat colour,roan	coat_roan6	Warmblood	0.071	KIT	CC	CC	T	C	chr3:79540110
Coat colour,roan	coat_roan6	WP	0.125	KIT	CC	CC	T	C	chr3:79540110
Coat colour,roan	coat_roan6	Clydesdale	0.000	KIT	CC	CC	T	C	chr3:79540110
Coat colour,roan	coat_roan6	Icelandic	0.206	KIT	CC	CC	T	C	chr3:79540110
Coat colour,roan	coat_roan6	Shetland	0.327	KIT	CC	CC	T	C	chr3:79540110
Coat colour,roan	coat_roan6	Arabian	0.079	KIT	CC	CC	T	C	chr3:79540110
Coat colour,roan	coat_roan6	Morgan	0.068	KIT	CC	CC	T	C	chr3:79540110
Coat colour,roan	coat_roan6	Other	0.191	KIT	CC	CC	T	C	chr3:79540110
Coat colour,roan	coat_roan7	Warmblood	0.086	KIT	CC	CC	G	A	chr3:79540020
Coat colour,roan	coat_roan7	TB	0.158	KIT	CC	CC	G	A	chr3:79540020
Coat colour,roan	coat_roan7	Shetland	0.018	KIT	CC	CC	G	A	chr3:79540020
Coat colour,roan	coat_roan7	WP	0.125	KIT	CC	CC	G	A	chr3:79540020
Coat colour,roan	coat_roan7	Other	0.050	KIT	CC	CC	G	A	chr3:79540020
Coat colour,roan	coat_roan7	STB	0.000	KIT	CC	CC	G	A	chr3:79540020
Coat colour,roan	coat_roan7	Belgian	0.475	KIT	CC	CC	G	A	chr3:79540020
Coat colour,roan	coat_roan7	FM	0.150	KIT	CC	CC	G	A	chr3:79540020
Coat colour,roan	coat_roan7	Arabian	0.158	KIT	CC	CC	G	A	chr3:79540020
Coat colour,roan	coat_roan7	QH	0.080	KIT	CC	CC	G	A	chr3:79540020
Coat colour,roan	coat_roan7	Morgan	0.091	KIT	CC	CC	G	A	chr3:79540020
Coat colour,roan	coat_roan7	Clydesdale	0.000	KIT	CC	CC	G	A	chr3:79540020
Coat colour,roan	coat_roan7	Icelandic	0.294	KIT	CC	CC	G	A	chr3:79540020
Coat colour,roan	coat_roan8	Warmblood	0.000	KIT	CC	CC	T	C	chr3:79539989
Coat colour,roan	coat_roan8	Shetland	0.000	KIT	CC	CC	T	C	chr3:79539989
Coat colour,roan	coat_roan8	TB	0.046	KIT	CC	CC	T	C	chr3:79539989
Coat colour,roan	coat_roan8	QH	0.009	KIT	CC	CC	T	C	chr3:79539989
Coat colour,roan	coat_roan8	Arabian	0.000	KIT	CC	CC	T	C	chr3:79539989
Coat colour,roan	coat_roan8	Icelandic	0.176	KIT	CC	CC	T	C	chr3:79539989

NA: Variants not present in this population

Supplementary table 6. Breed specific allele frequencies for the known variants

Coat colour,roan	coat_roan8	Morgan	0.000	KIT	CC	CC	T	C	chr3:79539989
Coat colour,roan	coat_roan8	Other	0.005	KIT	CC	CC	T	C	chr3:79539989
Coat colour,roan	coat_roan8	WP	0.050	KIT	CC	CC	T	C	chr3:79539989
Coat colour,roan	coat_roan8	Clydesdale	0.000	KIT	CC	CC	T	C	chr3:79539989
Coat colour,roan	coat_roan8	FM	0.000	KIT	CC	CC	T	C	chr3:79539989
Coat colour,roan	coat_roan8	Belgian	0.025	KIT	CC	CC	T	C	chr3:79539989
Coat colour,roan	coat_roan8	STB	0.000	KIT	CC	CC	T	C	chr3:79539989
Coat colour,roan	coat_roan9	TB	0.053	KIT	CC	CC	C	T	chr3:79538738
Coat colour,roan	coat_roan9	Shetland	0.000	KIT	CC	CC	C	T	chr3:79538738
Coat colour,roan	coat_roan9	WP	0.050	KIT	CC	CC	C	T	chr3:79538738
Coat colour,roan	coat_roan9	Belgian	0.025	KIT	CC	CC	C	T	chr3:79538738
Coat colour,roan	coat_roan9	Arabian	0.000	KIT	CC	CC	C	T	chr3:79538738
Coat colour,roan	coat_roan9	FM	0.000	KIT	CC	CC	C	T	chr3:79538738
Coat colour,roan	coat_roan9	Icelandic	0.176	KIT	CC	CC	C	T	chr3:79538738
Coat colour,roan	coat_roan9	Warmblood	0.000	KIT	CC	CC	C	T	chr3:79538738
Coat colour,roan	coat_roan9	Other	0.041	KIT	CC	CC	C	T	chr3:79538738
Coat colour,roan	coat_roan9	STB	0.008	KIT	CC	CC	C	T	chr3:79538738
Coat colour,roan	coat_roan9	QH	0.066	KIT	CC	CC	C	T	chr3:79538738
Coat colour,roan	coat_roan9	Clydesdale	0.000	KIT	CC	CC	C	T	chr3:79538738
Coat colour,roan	coat_roan9	Morgan	0.000	KIT	CC	CC	C	T	chr3:79538738
Coat colour, dominant white	coat_SB1	Icelandic	0.000	KIT	CC	CC	A	T	chr3:79544206
Coat colour, dominant white	coat_SB1	Warmblood	0.000	KIT	CC	CC	A	T	chr3:79544206
Coat colour, dominant white	coat_SB1	Clydesdale	0.000	KIT	CC	CC	A	T	chr3:79544206
Coat colour, dominant white	coat_SB1	TB	0.000	KIT	CC	CC	A	T	chr3:79544206
Coat colour, dominant white	coat_SB1	Belgian	0.000	KIT	CC	CC	A	T	chr3:79544206
Coat colour, dominant white	coat_SB1	STB	0.000	KIT	CC	CC	A	T	chr3:79544206
Coat colour, dominant white	coat_SB1	QH	0.005	KIT	CC	CC	A	T	chr3:79544206
Coat colour, dominant white	coat_SB1	Arabian	0.000	KIT	CC	CC	A	T	chr3:79544206
Coat colour, dominant white	coat_SB1	WP	0.000	KIT	CC	CC	A	T	chr3:79544206
Coat colour, dominant white	coat_SB1	FM	0.000	KIT	CC	CC	A	T	chr3:79544206

NA: Variants not present in this population

Supplementary table 6. Breed specific allele frequencies for the known variants

Coat colour, dominant white	coat_SB1	Shetland	0.000	KIT	CC	CC	A	T	chr3:79544206
Coat colour, dominant white	coat_SB1	Other	0.000	KIT	CC	CC	A	T	chr3:79544206
Coat colour, dominant white	coat_SB1	Morgan	0.000	KIT	CC	CC	A	T	chr3:79544206
Coat colour, cream dilution	coat_snow	NA	NA	SLC45A2	CC	CC	G	A	chr21:31688649
Coat colour, cream dilution	coat_sun	TB	0.862	SLC45A2	CC	CC	G	A	chr21:31705726
Coat colour, cream dilution	coat_sun	Belgian	0.875	SLC45A2	CC	CC	G	A	chr21:31705726
Coat colour, cream dilution	coat_sun	Warmblood	0.714	SLC45A2	CC	CC	G	A	chr21:31705726
Coat colour, cream dilution	coat_sun	Arabian	0.855	SLC45A2	CC	CC	G	A	chr21:31705726
Coat colour, cream dilution	coat_sun	QH	0.863	SLC45A2	CC	CC	G	A	chr21:31705726
Coat colour, cream dilution	coat_sun	Clydesdale	0.684	SLC45A2	CC	CC	G	A	chr21:31705726
Coat colour, cream dilution	coat_sun	Icelandic	0.706	SLC45A2	CC	CC	G	A	chr21:31705726
Coat colour, cream dilution	coat_sun	Other	0.745	SLC45A2	CC	CC	G	A	chr21:31705726
Coat colour, cream dilution	coat_sun	FM	0.583	SLC45A2	CC	CC	G	A	chr21:31705726
Coat colour, cream dilution	coat_sun	Shetland	0.764	SLC45A2	CC	CC	G	A	chr21:31705726
Coat colour, cream dilution	coat_sun	WP	0.775	SLC45A2	CC	CC	G	A	chr21:31705726
Coat colour, cream dilution	coat_sun	Morgan	0.886	SLC45A2	CC	CC	G	A	chr21:31705726
Coat colour, cream dilution	coat_sun	STB	0.644	SLC45A2	CC	CC	G	A	chr21:31705726
Coat colour,white spotting	coat_SW1	Belgian	0.000	MITF	CC	CC	T	ATAATAACC	chr16:21579201
Coat colour,white spotting	coat_SW1	QH	0.014	MITF	CC	CC	T	ATAATAACC	chr16:21579201
Coat colour,white spotting	coat_SW1	FM	0.000	MITF	CC	CC	T	ATAATAACC	chr16:21579201
Coat colour,white spotting	coat_SW1	Icelandic	0.000	MITF	CC	CC	T	ATAATAACC	chr16:21579201
Coat colour,white spotting	coat_SW1	TB	0.000	MITF	CC	CC	T	ATAATAACC	chr16:21579201
Coat colour,white spotting	coat_SW1	Arabian	0.000	MITF	CC	CC	T	ATAATAACC	chr16:21579201
Coat colour,white spotting	coat_SW1	Shetland	0.000	MITF	CC	CC	T	ATAATAACC	chr16:21579201
Coat colour,white	coat_SW1	WP	0.000	MITF	CC	CC	T	ATAATAACC	chr16:21579201

NA: Variants not present in this population

Supplementary table 6. Breed specific allele frequencies for the known variants

spotting									
Coat colour,white spotting	coat_SW1	Other	0.005	MITF	CC	CC	T	ATAATAACC	chr16:21579201
Coat colour,white spotting	coat_SW1	Clydesdale	0.000	MITF	CC	CC	T	ATAATAACC	chr16:21579201
Coat colour,white spotting	coat_SW1	Morgan	0.000	MITF	CC	CC	T	ATAATAACC	chr16:21579201
Coat colour,white spotting	coat_SW1	Warmblood	0.000	MITF	CC	CC	T	ATAATAACC	chr16:21579201
Coat colour,white spotting	coat_SW1	STB	0.000	MITF	CC	CC	T	ATAATAACC	chr16:21579201
Coat colour, white spotting, PAX3-related	coat_SW2	Clydesdale	0.000	PAX3	CC	CC	G	A	chr6:11199026
Coat colour, white spotting, PAX3-related	coat_SW2	Belgian	0.000	PAX3	CC	CC	G	A	chr6:11199026
Coat colour, white spotting, PAX3-related	coat_SW2	QH	0.009	PAX3	CC	CC	G	A	chr6:11199026
Coat colour, white spotting, PAX3-related	coat_SW2	Shetland	0.000	PAX3	CC	CC	G	A	chr6:11199026
Coat colour, white spotting, PAX3-related	coat_SW2	FM	0.000	PAX3	CC	CC	G	A	chr6:11199026
Coat colour, white spotting, PAX3-related	coat_SW2	Arabian	0.000	PAX3	CC	CC	G	A	chr6:11199026
Coat colour, white spotting, PAX3-related	coat_SW2	Morgan	0.000	PAX3	CC	CC	G	A	chr6:11199026
Coat colour, white spotting, PAX3-related	coat_SW2	TB	0.000	PAX3	CC	CC	G	A	chr6:11199026
Coat colour, white spotting, PAX3-related	coat_SW2	Icelandic	0.000	PAX3	CC	CC	G	A	chr6:11199026
Coat colour, white spotting, PAX3-related	coat_SW2	WP	0.000	PAX3	CC	CC	G	A	chr6:11199026
Coat colour, white spotting, PAX3-related	coat_SW2	Other	0.000	PAX3	CC	CC	G	A	chr6:11199026
Coat colour, white spotting, PAX3-related	coat_SW2	Warmblood	0.000	PAX3	CC	CC	G	A	chr6:11199026
Coat colour, white spotting, PAX3-related	coat_SW2	STB	0.000	PAX3	CC	CC	G	A	chr6:11199026
Coat colour,white spotting	coat_SW3	NA	NA	MITF	CC	CC	GTGTC	*	chr16:21567245
Coat colour, white spotting, PAX3-related	coat_SW4	NA	NA	PAX3	CC	CC	G	C	chr6:11199140

NA: Variants not present in this population

Supplementary table 6. Breed specific allele frequencies for the known variants

Coat colour,white spotting	coat_W	NA	NA	MITF	CC	CC	C	G	chr16:21565012
Coat colour, dominant white	coat_W1	NA	NA	KIT	CC	CC	G	C	chr3:79545942
Coat colour, dominant white	coat_W10	NA	NA	KIT	CC	CC	GAAC	*	chr3:79566926
Coat colour, dominant white	coat_W11	NA	NA	KIT	CC	CC	C	T	chr3:79540429
Coat colour, dominant white	coat_W12	NA	NA	KIT	CC	CC	TCTGC	*	chr3:79579774
Coat colour, dominant white	coat_W13	NA	NA	KIT	CC	CC	C	G	chr3:79544066
Coat colour, dominant white	coat_W15	NA	NA	KIT	CC	CC	A	G	chr3:79550351
Coat colour, dominant white	coat_W16	NA	NA	KIT	CC	CC	T	A	chr3:79540741
Coat colour, dominant white	coat_W17a	NA	NA	KIT	CC	CC	T	C	chr3:79548265
Coat colour, dominant white	coat_W17b	NA	NA	KIT	CC	CC	A	G	chr3:79548244
Coat colour, dominant white	coat_W18	NA	NA	KIT	CC	CC	C	T	chr3:79553752
Coat colour, dominant white	coat_W19	NA	NA	KIT	CC	CC	T	C	chr3:79553776
Coat colour, dominant white	coat_W2	NA	NA	KIT	CC	CC	C	T	chr3:79549540
Coat colour, dominant white	coat_W20	Icelandic	0.000	KIT	CC	CC	T	C	chr3:79548220
Coat colour, dominant white	coat_W20	FM	0.167	KIT	CC	CC	T	C	chr3:79548220
Coat colour, dominant white	coat_W20	Morgan	0.159	KIT	CC	CC	T	C	chr3:79548220
Coat colour, dominant white	coat_W20	TB	0.309	KIT	CC	CC	T	C	chr3:79548220
Coat colour, dominant white	coat_W20	Belgian	0.100	KIT	CC	CC	T	C	chr3:79548220
Coat colour, dominant white	coat_W20	WP	0.175	KIT	CC	CC	T	C	chr3:79548220
Coat colour, dominant white	coat_W20	Other	0.177	KIT	CC	CC	T	C	chr3:79548220
Coat colour, dominant white	coat_W20	Arabian	0.013	KIT	CC	CC	T	C	chr3:79548220
Coat colour, dominant white	coat_W20	Shetland	0.218	KIT	CC	CC	T	C	chr3:79548220
Coat colour, dominant white	coat_W20	STB	0.136	KIT	CC	CC	T	C	chr3:79548220
Coat colour, dominant white	coat_W20	Warmblood	0.186	KIT	CC	CC	T	C	chr3:79548220
Coat colour, dominant	coat_W20	QH	0.165	KIT	CC	CC	T	C	chr3:79548220

NA: Variants not present in this population

Supplementary table 6. Breed specific allele frequencies for the known variants

white									
Coat colour, dominant white	coat_W20	Clydesdale	0.947	KIT	CC	CC	T	C	chr3:79548220
Coat colour, dominant white	coat_W21	Other	0.000	KIT	CC	CC	C	*	chr3:79544174
Coat colour, dominant white	coat_W21	QH	0.000	KIT	CC	CC	C	*	chr3:79544174
Coat colour, dominant white	coat_W21	Belgian	0.000	KIT	CC	CC	C	*	chr3:79544174
Coat colour, dominant white	coat_W21	Icelandic	0.059	KIT	CC	CC	C	*	chr3:79544174
Coat colour, dominant white	coat_W21	FM	0.000	KIT	CC	CC	C	*	chr3:79544174
Coat colour, dominant white	coat_W21	STB	0.000	KIT	CC	CC	C	*	chr3:79544174
Coat colour, dominant white	coat_W21	Arabian	0.000	KIT	CC	CC	C	*	chr3:79544174
Coat colour, dominant white	coat_W21	Morgan	0.000	KIT	CC	CC	C	*	chr3:79544174
Coat colour, dominant white	coat_W21	TB	0.000	KIT	CC	CC	C	*	chr3:79544174
Coat colour, dominant white	coat_W21	Clydesdale	0.000	KIT	CC	CC	C	*	chr3:79544174
Coat colour, dominant white	coat_W21	Shetland	0.000	KIT	CC	CC	C	*	chr3:79544174
Coat colour, dominant white	coat_W21	Warmblood	0.000	KIT	CC	CC	C	*	chr3:79544174
Coat colour, dominant white	coat_W21	WP	0.000	KIT	CC	CC	C	*	chr3:79544174
Coat colour, dominant white	coat_W23	NA	NA	KIT	CC	CC	C	G	chr3:79578484
Coat colour, dominant white	coat_W24	Other	0.005	KIT	CC	CC	C	T	chr3:79545245
Coat colour, dominant white	coat_W24	QH	0.000	KIT	CC	CC	C	T	chr3:79545245
Coat colour, dominant white	coat_W24	Arabian	0.000	KIT	CC	CC	C	T	chr3:79545245
Coat colour, dominant white	coat_W24	Belgian	0.000	KIT	CC	CC	C	T	chr3:79545245
Coat colour, dominant white	coat_W24	STB	0.000	KIT	CC	CC	C	T	chr3:79545245
Coat colour, dominant white	coat_W24	Shetland	0.000	KIT	CC	CC	C	T	chr3:79545245
Coat colour, dominant white	coat_W24	FM	0.000	KIT	CC	CC	C	T	chr3:79545245
Coat colour, dominant white	coat_W24	Clydesdale	0.000	KIT	CC	CC	C	T	chr3:79545245
Coat colour, dominant white	coat_W24	Morgan	0.000	KIT	CC	CC	C	T	chr3:79545245

NA: Variants not present in this population

Supplementary table 6. Breed specific allele frequencies for the known variants

Coat colour, dominant white	coat_W24	TB	0.000	KIT	CC	CC	C	T	chr3:79545245
Coat colour, dominant white	coat_W24	Icelandic	0.000	KIT	CC	CC	C	T	chr3:79545245
Coat colour, dominant white	coat_W24	WP	0.000	KIT	CC	CC	C	T	chr3:79545245
Coat colour, dominant white	coat_W24	Warmblood	0.000	KIT	CC	CC	C	T	chr3:79545245
Coat colour, dominant white	coat_W25	NA	NA	KIT	CC	CC	A	G	chr3:79578573
Coat colour, dominant white	coat_W26	NA	NA	KIT	CC	CC	T	*	chr3:79540693
Coat colour, dominant white	coat_W27	NA	NA	KIT	CC	CC	A	C	chr3:79551937
Coat colour, dominant white	coat_W3	NA	NA	KIT	CC	CC	T	A	chr3:79578535
Coat colour, dominant white	coat_W30	NA	NA	KIT	CC	CC	A	T	chr3:79548244
Coat colour, dominant white	coat_W31	NA	NA	KIT	CC	CC	T	TT	chr3:79618532
Coat colour, dominant white	coat_W32	QH	0.066	KIT	CC	CC	C	T	chr3:79538738
Coat colour, dominant white	coat_W32	STB	0.008	KIT	CC	CC	C	T	chr3:79538738
Coat colour, dominant white	coat_W32	Icelandic	0.176	KIT	CC	CC	C	T	chr3:79538738
Coat colour, dominant white	coat_W32	Morgan	0.000	KIT	CC	CC	C	T	chr3:79538738
Coat colour, dominant white	coat_W32	Arabian	0.000	KIT	CC	CC	C	T	chr3:79538738
Coat colour, dominant white	coat_W32	Belgian	0.025	KIT	CC	CC	C	T	chr3:79538738
Coat colour, dominant white	coat_W32	FM	0.000	KIT	CC	CC	C	T	chr3:79538738
Coat colour, dominant white	coat_W32	Shetland	0.000	KIT	CC	CC	C	T	chr3:79538738
Coat colour, dominant white	coat_W32	Clydesdale	0.000	KIT	CC	CC	C	T	chr3:79538738
Coat colour, dominant white	coat_W32	Warmblood	0.000	KIT	CC	CC	C	T	chr3:79538738
Coat colour, dominant white	coat_W32	Other	0.041	KIT	CC	CC	C	T	chr3:79538738
Coat colour, dominant white	coat_W32	TB	0.053	KIT	CC	CC	C	T	chr3:79538738
Coat colour, dominant white	coat_W32	WP	0.050	KIT	CC	CC	C	T	chr3:79538738
Coat colour, dominant white	coat_W33	NA	NA	KIT	CC	CC	T	A	chr3:79545248
Coat colour, dominant	coat_W34	Shetland	0.000	KIT	CC	CC	T	C	chr3:79566881

NA: Variants not present in this population

Supplementary table 6. Breed specific allele frequencies for the known variants

white									
Coat colour, dominant white	coat_W34	Arabian	0.053	KIT	CC	CC	T	C	chr3:79566881
Coat colour, dominant white	coat_W34	Icelandic	0.000	KIT	CC	CC	T	C	chr3:79566881
Coat colour, dominant white	coat_W34	Other	0.000	KIT	CC	CC	T	C	chr3:79566881
Coat colour, dominant white	coat_W34	TB	0.000	KIT	CC	CC	T	C	chr3:79566881
Coat colour, dominant white	coat_W34	Clydesdale	0.000	KIT	CC	CC	T	C	chr3:79566881
Coat colour, dominant white	coat_W34	Morgan	0.045	KIT	CC	CC	T	C	chr3:79566881
Coat colour, dominant white	coat_W34	Belgian	0.000	KIT	CC	CC	T	C	chr3:79566881
Coat colour, dominant white	coat_W34	FM	0.000	KIT	CC	CC	T	C	chr3:79566881
Coat colour, dominant white	coat_W34	Warmblood	0.100	KIT	CC	CC	T	C	chr3:79566881
Coat colour, dominant white	coat_W34	QH	0.000	KIT	CC	CC	T	C	chr3:79566881
Coat colour, dominant white	coat_W34	WP	0.000	KIT	CC	CC	T	C	chr3:79566881
Coat colour, dominant white	coat_W34	STB	0.017	KIT	CC	CC	T	C	chr3:79566881
Coat colour, dominant white	coat_W4	NA	NA	KIT	CC	CC	G	A	chr3:79549780
Coat colour, dominant white	coat_W5	NA	NA	KIT	CC	CC	G	*	chr3:79545900
Coat colour, dominant white	coat_W6	NA	NA	KIT	CC	CC	G	A	chr3:79573754
Coat colour, dominant white	coat_W7	NA	NA	KIT	CC	CC	C	G	chr3:79580000
Coat colour, dominant white	coat_W8	NA	NA	KIT	CC	CC	C	T	chr3:79545374
Coat colour, dominant white	coat_W9	NA	NA	KIT	CC	CC	C	T	chr3:79549797
Coat colour,white spotting	coat_white_splash	QH	0.745	MITF	CC	CC	C	A	chr16:21608936
Coat colour,white spotting	coat_white_splash	TB	0.500	MITF	CC	CC	C	A	chr16:21608936
Coat colour,white spotting	coat_white_splash	FM	0.567	MITF	CC	CC	C	A	chr16:21608936
Coat colour,white spotting	coat_white_splash	Belgian	0.850	MITF	CC	CC	C	A	chr16:21608936
Coat colour,white spotting	coat_white_splash	Icelandic	1.000	MITF	CC	CC	C	A	chr16:21608936
Coat colour,white spotting	coat_white_splash	Shetland	0.764	MITF	CC	CC	C	A	chr16:21608936

NA: Variants not present in this population

Supplementary table 6. Breed specific allele frequencies for the known variants

Coat colour,white spotting	coat_white_splasl	WP	0.925	MITF	CC	CC	C	A	chr16:21608936
Coat colour,white spotting	coat_white_splasl	Arabian	0.842	MITF	CC	CC	C	A	chr16:21608936
Coat colour,white spotting	coat_white_splasl	Clydesdale	0.500	MITF	CC	CC	C	A	chr16:21608936
Coat colour,white spotting	coat_white_splasl	Morgan	0.886	MITF	CC	CC	C	A	chr16:21608936
Coat colour,white spotting	coat_white_splasl	Warmblood	0.757	MITF	CC	CC	C	A	chr16:21608936
Coat colour,white spotting	coat_white_splasl	STB	0.890	MITF	CC	CC	C	A	chr16:21608936
Coat colour,white spotting	coat_white_splasl	Other	0.782	MITF	CC	CC	C	A	chr16:21608936
Dwarfism, ACAN-related	dwarf_D1	Warmblood	0.000	ACAN	yes	yes	CT	C	chr1:95291269
Dwarfism, ACAN-related	dwarf_D1	Belgian	0.000	ACAN	yes	yes	CT	C	chr1:95291269
Dwarfism, ACAN-related	dwarf_D1	FM	0.000	ACAN	yes	yes	CT	C	chr1:95291269
Dwarfism, ACAN-related	dwarf_D1	Other	0.000	ACAN	yes	yes	CT	C	chr1:95291269
Dwarfism, ACAN-related	dwarf_D1	QH	0.000	ACAN	yes	yes	CT	C	chr1:95291269
Dwarfism, ACAN-related	dwarf_D1	Shetland	0.009	ACAN	yes	yes	CT	C	chr1:95291269
Dwarfism, ACAN-related	dwarf_D1	Icelandic	0.000	ACAN	yes	yes	CT	C	chr1:95291269
Dwarfism, ACAN-related	dwarf_D1	Arabian	0.000	ACAN	yes	yes	CT	C	chr1:95291269
Dwarfism, ACAN-related	dwarf_D1	Clydesdale	0.000	ACAN	yes	yes	CT	C	chr1:95291269
Dwarfism, ACAN-related	dwarf_D1	Morgan	0.000	ACAN	yes	yes	CT	C	chr1:95291269
Dwarfism, ACAN-related	dwarf_D1	TB	0.000	ACAN	yes	yes	CT	C	chr1:95291269
Dwarfism, ACAN-related	dwarf_D1	WP	0.000	ACAN	yes	yes	CT	C	chr1:95291269
Dwarfism, ACAN-related	dwarf_D1	STB	0.000	ACAN	yes	yes	CT	C	chr1:95291269
Dwarfism, ACAN-related	dwarf_D2	QH	0.014	ACAN	yes	yes	C	T	chr1:95284530
Dwarfism, ACAN-related	dwarf_D2	Belgian	0.000	ACAN	yes	yes	C	T	chr1:95284530
Dwarfism, ACAN-related	dwarf_D2	Icelandic	0.000	ACAN	yes	yes	C	T	chr1:95284530
Dwarfism, ACAN-related	dwarf_D2	Other	0.000	ACAN	yes	yes	C	T	chr1:95284530
Dwarfism, ACAN-	dwarf_D2	TB	0.000	ACAN	yes	yes	C	T	chr1:95284530

NA: Variants not present in this population

Supplementary table 6. Breed specific allele frequencies for the known variants

related									
Dwarfism, ACAN-related	dwarf_D2	FM	0.000	ACAN	yes	yes	C	T	chr1:95284530
Dwarfism, ACAN-related	dwarf_D2	Morgan	0.000	ACAN	yes	yes	C	T	chr1:95284530
Dwarfism, ACAN-related	dwarf_D2	Warmblood	0.014	ACAN	yes	yes	C	T	chr1:95284530
Dwarfism, ACAN-related	dwarf_D2	STB	0.000	ACAN	yes	yes	C	T	chr1:95284530
Dwarfism, ACAN-related	dwarf_D2	Shetland	0.000	ACAN	yes	yes	C	T	chr1:95284530
Dwarfism, ACAN-related	dwarf_D2	WP	0.000	ACAN	yes	yes	C	T	chr1:95284530
Dwarfism, ACAN-related	dwarf_D2	Clydesdale	0.000	ACAN	yes	yes	C	T	chr1:95284530
Dwarfism, ACAN-related	dwarf_D2	Arabian	0.000	ACAN	yes	yes	C	T	chr1:95284530
Dwarfism, ACAN-related	dwarf_D3	Other	0.000	ACAN	yes	yes	C	G	chr1:95282140
Dwarfism, ACAN-related	dwarf_D3	QH	0.000	ACAN	yes	yes	C	G	chr1:95282140
Dwarfism, ACAN-related	dwarf_D3	Icelandic	0.000	ACAN	yes	yes	C	G	chr1:95282140
Dwarfism, ACAN-related	dwarf_D3	Warmblood	0.000	ACAN	yes	yes	C	G	chr1:95282140
Dwarfism, ACAN-related	dwarf_D3	Belgian	0.000	ACAN	yes	yes	C	G	chr1:95282140
Dwarfism, ACAN-related	dwarf_D3	Shetland	0.018	ACAN	yes	yes	C	G	chr1:95282140
Dwarfism, ACAN-related	dwarf_D3	TB	0.000	ACAN	yes	yes	C	G	chr1:95282140
Dwarfism, ACAN-related	dwarf_D3	STB	0.000	ACAN	yes	yes	C	G	chr1:95282140
Dwarfism, ACAN-related	dwarf_D3	WP	0.000	ACAN	yes	yes	C	G	chr1:95282140
Dwarfism, ACAN-related	dwarf_D3	FM	0.000	ACAN	yes	yes	C	G	chr1:95282140
Dwarfism, ACAN-related	dwarf_D3	Morgan	0.000	ACAN	yes	yes	C	G	chr1:95282140
Dwarfism, ACAN-related	dwarf_D3	Arabian	0.000	ACAN	yes	yes	C	G	chr1:95282140
Dwarfism, ACAN-related	dwarf_D3	Clydesdale	0.000	ACAN	yes	yes	C	G	chr1:95282140
Dwarfism, ACAN-related	dwarf_D4	NA	NA	ACAN	yes	yes	CGCAGTCC	C	chr1:95257478
Dwarfism, Friesian	dwarf_fr	QH	0.000	B4GALT7	yes	yes	C	T	chr14:3772591
Dwarfism, Friesian	dwarf_fr	TB	0.000	B4GALT7	yes	yes	C	T	chr14:3772591
Dwarfism, Friesian	dwarf_fr	Icelandic	0.000	B4GALT7	yes	yes	C	T	chr14:3772591

NA: Variants not present in this population

Supplementary table 6. Breed specific allele frequencies for the known variants

Dwarfism, Friesian	dwarf_fr	Other	0.009	B4GALT7	yes	yes	C	T	chr14:3772591
Dwarfism, Friesian	dwarf_fr	STB	0.000	B4GALT7	yes	yes	C	T	chr14:3772591
Dwarfism, Friesian	dwarf_fr	Shetland	0.000	B4GALT7	yes	yes	C	T	chr14:3772591
Dwarfism, Friesian	dwarf_fr	WP	0.000	B4GALT7	yes	yes	C	T	chr14:3772591
Dwarfism, Friesian	dwarf_fr	Warmblood	0.000	B4GALT7	yes	yes	C	T	chr14:3772591
Dwarfism, Friesian	dwarf_fr	Belgian	0.000	B4GALT7	yes	yes	C	T	chr14:3772591
Dwarfism, Friesian	dwarf_fr	FM	0.000	B4GALT7	yes	yes	C	T	chr14:3772591
Dwarfism, Friesian	dwarf_fr	Morgan	0.000	B4GALT7	yes	yes	C	T	chr14:3772591
Dwarfism, Friesian	dwarf_fr	Arabian	0.000	B4GALT7	yes	yes	C	T	chr14:3772591
Dwarfism, Friesian	dwarf_fr	Clydesdale	0.000	B4GALT7	yes	yes	C	T	chr14:3772591
Dwarfism	Dwarf_G	TB	0.000	GHI1	yes	no	C	T	chr11:15494088
Dwarfism	Dwarf_G	Belgian	0.000	GHI1	yes	no	C	T	chr11:15494088
Dwarfism	Dwarf_G	QH	0.000	GHI1	yes	no	C	T	chr11:15494088
Dwarfism	Dwarf_G	Shetland	0.127	GHI1	yes	no	C	T	chr11:15494088
Dwarfism	Dwarf_G	STB	0.000	GHI1	yes	no	C	T	chr11:15494088
Dwarfism	Dwarf_G	Warmblood	0.000	GHI1	yes	no	C	T	chr11:15494088
Dwarfism	Dwarf_G	Icelandic	0.000	GHI1	yes	no	C	T	chr11:15494088
Dwarfism	Dwarf_G	Other	0.018	GHI1	yes	no	C	T	chr11:15494088
Dwarfism	Dwarf_G	Clydesdale	0.000	GHI1	yes	no	C	T	chr11:15494088
Dwarfism	Dwarf_G	WP	0.000	GHI1	yes	no	C	T	chr11:15494088
Dwarfism	Dwarf_G	Morgan	0.000	GHI1	yes	no	C	T	chr11:15494088
Dwarfism	Dwarf_G	Arabian	0.000	GHI1	yes	no	C	T	chr11:15494088
Dwarfism	Dwarf_G	FM	0.000	GHI1	yes	no	C	T	chr11:15494088
Encephalomyelopathy	EDM	TB	0.158	ADGRL3	yes	no	T	C	chr3:73634146
Encephalomyelopathy	EDM	Warmblood	0.171	ADGRL3	yes	no	T	C	chr3:73634146
Encephalomyelopathy	EDM	QH	0.189	ADGRL3	yes	no	T	C	chr3:73634146
Encephalomyelopathy	EDM	Shetland	0.027	ADGRL3	yes	no	T	C	chr3:73634146
Encephalomyelopathy	EDM	WP	0.075	ADGRL3	yes	no	T	C	chr3:73634146
Encephalomyelopathy	EDM	Belgian	0.225	ADGRL3	yes	no	T	C	chr3:73634146
Encephalomyelopathy	EDM	FM	0.117	ADGRL3	yes	no	T	C	chr3:73634146
Encephalomyelopathy	EDM	Icelandic	0.206	ADGRL3	yes	no	T	C	chr3:73634146
Encephalomyelopathy	EDM	Other	0.059	ADGRL3	yes	no	T	C	chr3:73634146
Encephalomyelopathy	EDM	Arabian	0.053	ADGRL3	yes	no	T	C	chr3:73634146
Encephalomyelopathy	EDM	Clydesdale	0.000	ADGRL3	yes	no	T	C	chr3:73634146

NA: Variants not present in this population

Supplementary table 6. Breed specific allele frequencies for the known variants

Encephalomyelopathy	EDM	Morgan	0.091	ADGRL3	yes	no	T	C	chr3:73634146
Encephalomyelopathy	EDM	STB	0.025	ADGRL3	yes	no	T	C	chr3:73634146
Metabolic Syndrome	EMS	Icelandic	0.000	FAM174A	yes	no	T	C	chr14:69289779
Metabolic Syndrome	EMS	Warmblood	0.014	FAM174A	yes	no	T	C	chr14:69289779
Metabolic Syndrome	EMS	WP	0.100	FAM174A	yes	no	T	C	chr14:69289779
Metabolic Syndrome	EMS	FM	0.000	FAM174A	yes	no	T	C	chr14:69289779
Metabolic Syndrome	EMS	TB	0.007	FAM174A	yes	no	T	C	chr14:69289779
Metabolic Syndrome	EMS	Shetland	0.009	FAM174A	yes	no	T	C	chr14:69289779
Metabolic Syndrome	EMS	Belgian	0.000	FAM174A	yes	no	T	C	chr14:69289779
Metabolic Syndrome	EMS	QH	0.009	FAM174A	yes	no	T	C	chr14:69289779
Metabolic Syndrome	EMS	Arabian	0.237	FAM174A	yes	no	T	C	chr14:69289779
Metabolic Syndrome	EMS	STB	0.000	FAM174A	yes	no	T	C	chr14:69289779
Metabolic Syndrome	EMS	Morgan	0.000	FAM174A	yes	no	T	C	chr14:69289779
Metabolic Syndrome	EMS	Other	0.073	FAM174A	yes	no	T	C	chr14:69289779
Metabolic Syndrome	EMS	Clydesdale	0.026	FAM174A	yes	no	T	C	chr14:69289779
Recurrent uveitis	ERU1	Warmblood	0.886	NA	yes	no	G	A	chr20:50364059
Recurrent uveitis	ERU1	Icelandic	0.941	NA	yes	no	G	A	chr20:50364059
Recurrent uveitis	ERU1	TB	0.836	NA	yes	no	G	A	chr20:50364059
Recurrent uveitis	ERU1	Arabian	0.895	NA	yes	no	G	A	chr20:50364059
Recurrent uveitis	ERU1	Other	0.768	NA	yes	no	G	A	chr20:50364059
Recurrent uveitis	ERU1	Belgian	1.000	NA	yes	no	G	A	chr20:50364059
Recurrent uveitis	ERU1	WP	0.925	NA	yes	no	G	A	chr20:50364059
Recurrent uveitis	ERU1	Clydesdale	1.000	NA	yes	no	G	A	chr20:50364059
Recurrent uveitis	ERU1	QH	0.835	NA	yes	no	G	A	chr20:50364059
Recurrent uveitis	ERU1	Shetland	0.827	NA	yes	no	G	A	chr20:50364059
Recurrent uveitis	ERU1	STB	0.856	NA	yes	no	G	A	chr20:50364059
Recurrent uveitis	ERU1	FM	0.917	NA	yes	no	G	A	chr20:50364059
Recurrent uveitis	ERU1	Morgan	0.955	NA	yes	no	G	A	chr20:50364059
Recurrent uveitis	ERU2	TB	0.007	MHC1	yes	no	C	T	chr1:109163268
Recurrent uveitis	ERU2	QH	0.014	MHC1	yes	no	C	T	chr1:109163268
Recurrent uveitis	ERU2	Arabian	0.000	MHC1	yes	no	C	T	chr1:109163268
Recurrent uveitis	ERU2	Warmblood	0.000	MHC1	yes	no	C	T	chr1:109163268
Recurrent uveitis	ERU2	FM	0.133	MHC1	yes	no	C	T	chr1:109163268
Recurrent uveitis	ERU2	Icelandic	0.000	MHC1	yes	no	C	T	chr1:109163268

NA: Variants not present in this population

Supplementary table 6. Breed specific allele frequencies for the known variants

Recurrent uveitis	ERU2	Other	0.032	MHC1	yes	no	C	T	chr1:109163268
Recurrent uveitis	ERU2	Belgian	0.000	MHC1	yes	no	C	T	chr1:109163268
Recurrent uveitis	ERU2	Shetland	0.018	MHC1	yes	no	C	T	chr1:109163268
Recurrent uveitis	ERU2	WP	0.025	MHC1	yes	no	C	T	chr1:109163268
Recurrent uveitis	ERU2	Clydesdale	0.000	MHC1	yes	no	C	T	chr1:109163268
Recurrent uveitis	ERU2	STB	0.008	MHC1	yes	no	C	T	chr1:109163268
Recurrent uveitis	ERU2	Morgan	0.136	MHC1	yes	no	C	T	chr1:109163268
Recurrent uveitis	ERU3	Belgian	0.425	TIMP2	yes	no	A	G	chr11:3817009
Recurrent uveitis	ERU3	Icelandic	0.559	TIMP2	yes	no	A	G	chr11:3817009
Recurrent uveitis	ERU3	QH	0.561	TIMP2	yes	no	A	G	chr11:3817009
Recurrent uveitis	ERU3	Arabian	0.803	TIMP2	yes	no	A	G	chr11:3817009
Recurrent uveitis	ERU3	Clydesdale	0.895	TIMP2	yes	no	A	G	chr11:3817009
Recurrent uveitis	ERU3	FM	0.400	TIMP2	yes	no	A	G	chr11:3817009
Recurrent uveitis	ERU3	Shetland	0.527	TIMP2	yes	no	A	G	chr11:3817009
Recurrent uveitis	ERU3	TB	0.303	TIMP2	yes	no	A	G	chr11:3817009
Recurrent uveitis	ERU3	Warmblood	0.414	TIMP2	yes	no	A	G	chr11:3817009
Recurrent uveitis	ERU3	WP	0.700	TIMP2	yes	no	A	G	chr11:3817009
Recurrent uveitis	ERU3	Morgan	0.636	TIMP2	yes	no	A	G	chr11:3817009
Recurrent uveitis	ERU3	Other	0.495	TIMP2	yes	no	A	G	chr11:3817009
Recurrent uveitis	ERU3	STB	0.466	TIMP2	yes	no	A	G	chr11:3817009
Ehlers-Danlos Syndrome, type VI	FFS	Belgian	0.000	PLOD1	yes	yes	C	T	chr2:39927817
Ehlers-Danlos Syndrome, type VI	FFS	TB	0.000	PLOD1	yes	yes	C	T	chr2:39927817
Ehlers-Danlos Syndrome, type VI	FFS	Icelandic	0.000	PLOD1	yes	yes	C	T	chr2:39927817
Ehlers-Danlos Syndrome, type VI	FFS	Arabian	0.000	PLOD1	yes	yes	C	T	chr2:39927817
Ehlers-Danlos Syndrome, type VI	FFS	QH	0.000	PLOD1	yes	yes	C	T	chr2:39927817
Ehlers-Danlos Syndrome, type VI	FFS	FM	0.000	PLOD1	yes	yes	C	T	chr2:39927817
Ehlers-Danlos Syndrome, type VI	FFS	WP	0.000	PLOD1	yes	yes	C	T	chr2:39927817
Ehlers-Danlos Syndrome, type VI	FFS	Other	0.005	PLOD1	yes	yes	C	T	chr2:39927817
Ehlers-Danlos Syndrome, type VI	FFS	Clydesdale	0.000	PLOD1	yes	yes	C	T	chr2:39927817
Ehlers-Danlos Syndrome, type VI	FFS	Shetland	0.000	PLOD1	yes	yes	C	T	chr2:39927817

NA: Variants not present in this population

Supplementary table 6. Breed specific allele frequencies for the known variants

Ehlers-Danlos Syndrome, type VI	FFS	STB	0.000	PLOD1	yes	yes	C	T	chr2:39927817
Ehlers-Danlos Syndrome, type VI	FFS	Warmblood	0.057	PLOD1	yes	yes	C	T	chr2:39927817
Ehlers-Danlos Syndrome, type VI	FFS	Morgan	0.000	PLOD1	yes	yes	C	T	chr2:39927817
Foal immunodeficiency syndrome in the Fell and Dales Pony	FIS	NA	NA	SLC5A3	yes	yes	C	T	chr26:31894278
Gaitedness	gait	QH	0.052	LOC100147	yes	yes	C	A	chr23:22391254
Gaitedness	gait	Belgian	0.000	LOC100147	yes	yes	C	A	chr23:22391254
Gaitedness	gait	Icelandic	0.794	LOC100147	yes	yes	C	A	chr23:22391254
Gaitedness	gait	FM	0.000	LOC100147	yes	yes	C	A	chr23:22391254
Gaitedness	gait	TB	0.007	LOC100147	yes	yes	C	A	chr23:22391254
Gaitedness	gait	Arabian	0.000	LOC100147	yes	yes	C	A	chr23:22391254
Gaitedness	gait	Clydesdale	0.000	LOC100147	yes	yes	C	A	chr23:22391254
Gaitedness	gait	Shetland	0.000	LOC100147	yes	yes	C	A	chr23:22391254
Gaitedness	gait	Morgan	0.045	LOC100147	yes	yes	C	A	chr23:22391254
Gaitedness	gait	Warmblood	0.000	LOC100147	yes	yes	C	A	chr23:22391254
Gaitedness	gait	STB	0.873	LOC100147	yes	yes	C	A	chr23:22391254
Gaitedness	gait	WP	0.000	LOC100147	yes	yes	C	A	chr23:22391254
Gaitedness	gait	Other	0.141	LOC100147	yes	yes	C	A	chr23:22391254
Glycogen storage disease IV	GBED	Arabian	0.000	GBE1	yes	yes	C	A	chr26:8667651
Glycogen storage disease IV	GBED	Icelandic	0.000	GBE1	yes	yes	C	A	chr26:8667651
Glycogen storage disease IV	GBED	Other	0.000	GBE1	yes	yes	C	A	chr26:8667651
Glycogen storage disease IV	GBED	Belgian	0.000	GBE1	yes	yes	C	A	chr26:8667651
Glycogen storage disease IV	GBED	FM	0.000	GBE1	yes	yes	C	A	chr26:8667651
Glycogen storage disease IV	GBED	Clydesdale	0.000	GBE1	yes	yes	C	A	chr26:8667651
Glycogen storage disease IV	GBED	Morgan	0.000	GBE1	yes	yes	C	A	chr26:8667651
Glycogen storage disease IV	GBED	QH	0.028	GBE1	yes	yes	C	A	chr26:8667651
Glycogen storage disease IV	GBED	Shetland	0.000	GBE1	yes	yes	C	A	chr26:8667651
Glycogen storage disease IV	GBED	TB	0.000	GBE1	yes	yes	C	A	chr26:8667651
Glycogen storage	GBED	Warmblood	0.000	GBE1	yes	yes	C	A	chr26:8667651

NA: Variants not present in this population

Supplementary table 6. Breed specific allele frequencies for the known variants

disease IV									
Glycogen storage disease IV	GBED	STB	0.000	GBE1	yes	yes	C	A	chr26:8667651
Glycogen storage disease IV	GBED	WP	0.000	GBE1	yes	yes	C	A	chr26:8667651
Hemophilia A	HA_del	Belgian	0.075	FV111	yes	no	CTGTT	C	chrX:127502313
Hemophilia A	HA_del	Icelandic	0.059	FV111	yes	no	CTGTT	C	chrX:127502313
Hemophilia A	HA_del	Arabian	0.026	FV111	yes	no	CTGTT	C	chrX:127502313
Hemophilia A	HA_del	Clydesdale	0.026	FV111	yes	no	CTGTT	C	chrX:127502313
Hemophilia A	HA_del	FM	0.033	FV111	yes	no	CTGTT	C	chrX:127502313
Hemophilia A	HA_del	Morgan	0.023	FV111	yes	no	CTGTT	C	chrX:127502313
Hemophilia A	HA_del	QH	0.028	FV111	yes	no	CTGTT	C	chrX:127502313
Hemophilia A	HA_del	TB	0.007	FV111	yes	no	CTGTT	C	chrX:127502313
Hemophilia A	HA_del	Shetland	0.027	FV111	yes	no	CTGTT	C	chrX:127502313
Hemophilia A	HA_del	WP	0.050	FV111	yes	no	CTGTT	C	chrX:127502313
Hemophilia A	HA_del	Other	0.023	FV111	yes	no	CTGTT	C	chrX:127502313
Hemophilia A	HA_del	STB	0.008	FV111	yes	no	CTGTT	C	chrX:127502313
Hemophilia A	HA_del	Warmblood	0.043	FV111	yes	no	CTGTT	C	chrX:127502313
Hemophilia A	HA_SNP1	NA	NA	FV111	yes	no	C	G	chrX:127502303
Hemophilia A	HA_SNP2	NA	NA	FV111	yes	no	G	A	chrX:127502351
Ehlers-Danlos syndrome	HERDA	Belgian	0.000	PPIB	yes	yes	G	A	chr1:129307092
Ehlers-Danlos syndrome	HERDA	FM	0.000	PPIB	yes	yes	G	A	chr1:129307092
Ehlers-Danlos syndrome	HERDA	Arabian	0.000	PPIB	yes	yes	G	A	chr1:129307092
Ehlers-Danlos syndrome	HERDA	Clydesdale	0.000	PPIB	yes	yes	G	A	chr1:129307092
Ehlers-Danlos syndrome	HERDA	Morgan	0.000	PPIB	yes	yes	G	A	chr1:129307092
Ehlers-Danlos syndrome	HERDA	Icelandic	0.000	PPIB	yes	yes	G	A	chr1:129307092
Ehlers-Danlos syndrome	HERDA	Other	0.000	PPIB	yes	yes	G	A	chr1:129307092
Ehlers-Danlos syndrome	HERDA	QH	0.033	PPIB	yes	yes	G	A	chr1:129307092
Ehlers-Danlos syndrome	HERDA	Shetland	0.000	PPIB	yes	yes	G	A	chr1:129307092
Ehlers-Danlos syndrome	HERDA	STB	0.000	PPIB	yes	yes	G	A	chr1:129307092
Ehlers-Danlos syndrome	HERDA	WP	0.000	PPIB	yes	yes	G	A	chr1:129307092

NA: Variants not present in this population

Supplementary table 6. Breed specific allele frequencies for the known variants

Ehlers-Danlos syndrome	HERDA	TB	0.000	PPIB	yes	yes	G	A	chr1:129307092
Ehlers-Danlos syndrome	HERDA	Warmblood	0.000	PPIB	yes	yes	G	A	chr1:129307092
Hypoparathyroidism	HPT	NA	NA	RAPGEF5	yes	yes	G	T	chr4:54108297
Hoof wall separation syndrome	HWSD	Arabian	0.000	SERPINB11	yes	yes	G	GC	chr8:83600642
Hoof wall separation syndrome	HWSD	Belgian	0.000	SERPINB11	yes	yes	G	GC	chr8:83600642
Hoof wall separation syndrome	HWSD	TB	0.000	SERPINB11	yes	yes	G	GC	chr8:83600642
Hoof wall separation syndrome	HWSD	Clydesdale	0.000	SERPINB11	yes	yes	G	GC	chr8:83600642
Hoof wall separation syndrome	HWSD	Icelandic	0.000	SERPINB11	yes	yes	G	GC	chr8:83600642
Hoof wall separation syndrome	HWSD	QH	0.000	SERPINB11	yes	yes	G	GC	chr8:83600642
Hoof wall separation syndrome	HWSD	Other	0.023	SERPINB11	yes	yes	G	GC	chr8:83600642
Hoof wall separation syndrome	HWSD	STB	0.000	SERPINB11	yes	yes	G	GC	chr8:83600642
Hoof wall separation syndrome	HWSD	FM	0.000	SERPINB11	yes	yes	G	GC	chr8:83600642
Hoof wall separation syndrome	HWSD	Morgan	0.000	SERPINB11	yes	yes	G	GC	chr8:83600642
Hoof wall separation syndrome	HWSD	Warmblood	0.000	SERPINB11	yes	yes	G	GC	chr8:83600642
Hoof wall separation syndrome	HWSD	Shetland	0.000	SERPINB11	yes	yes	G	GC	chr8:83600642
Hoof wall separation syndrome	HWSD	WP	0.000	SERPINB11	yes	yes	G	GC	chr8:83600642
Hydrocephalus	hydro	Icelandic	0.000	B3GALNT2	yes	yes	C	T	chr1:76887901
Hydrocephalus	hydro	FM	0.000	B3GALNT2	yes	yes	C	T	chr1:76887901
Hydrocephalus	hydro	Arabian	0.000	B3GALNT2	yes	yes	C	T	chr1:76887901
Hydrocephalus	hydro	Other	0.000	B3GALNT2	yes	yes	C	T	chr1:76887901
Hydrocephalus	hydro	Belgian	0.000	B3GALNT2	yes	yes	C	T	chr1:76887901
Hydrocephalus	hydro	Shetland	0.000	B3GALNT2	yes	yes	C	T	chr1:76887901
Hydrocephalus	hydro	TB	0.000	B3GALNT2	yes	yes	C	T	chr1:76887901
Hydrocephalus	hydro	Clydesdale	0.000	B3GALNT2	yes	yes	C	T	chr1:76887901
Hydrocephalus	hydro	QH	0.000	B3GALNT2	yes	yes	C	T	chr1:76887901
Hydrocephalus	hydro	STB	0.000	B3GALNT2	yes	yes	C	T	chr1:76887901
Hydrocephalus	hydro	WP	0.000	B3GALNT2	yes	yes	C	T	chr1:76887901
Hydrocephalus	hydro	Warmblood	0.029	B3GALNT2	yes	yes	C	T	chr1:76887901

NA: Variants not present in this population

Supplementary table 6. Breed specific allele frequencies for the known variants

Hydrocephalus	hydro	Morgan	0.000	B3GALNT2	yes	yes	C	T	chr1:76887901
Hyperkalemic Periodic Paralysis, HYPP	HYPP	TB	0.000	SCN4A	yes	yes	C	G	chr11:15474228
Hyperkalemic Periodic Paralysis, HYPP	HYPP	Belgian	0.000	SCN4A	yes	yes	C	G	chr11:15474228
Hyperkalemic Periodic Paralysis, HYPP	HYPP	Icelandic	0.000	SCN4A	yes	yes	C	G	chr11:15474228
Hyperkalemic Periodic Paralysis, HYPP	HYPP	QH	0.014	SCN4A	yes	yes	C	G	chr11:15474228
Hyperkalemic Periodic Paralysis, HYPP	HYPP	Other	0.000	SCN4A	yes	yes	C	G	chr11:15474228
Hyperkalemic Periodic Paralysis, HYPP	HYPP	STB	0.000	SCN4A	yes	yes	C	G	chr11:15474228
Hyperkalemic Periodic Paralysis, HYPP	HYPP	FM	0.000	SCN4A	yes	yes	C	G	chr11:15474228
Hyperkalemic Periodic Paralysis, HYPP	HYPP	Morgan	0.000	SCN4A	yes	yes	C	G	chr11:15474228
Hyperkalemic Periodic Paralysis, HYPP	HYPP	Warmblood	0.000	SCN4A	yes	yes	C	G	chr11:15474228
Hyperkalemic Periodic Paralysis, HYPP	HYPP	Shetland	0.000	SCN4A	yes	yes	C	G	chr11:15474228
Hyperkalemic Periodic Paralysis, HYPP	HYPP	WP	0.000	SCN4A	yes	yes	C	G	chr11:15474228
Hyperkalemic Periodic Paralysis, HYPP	HYPP	Clydesdale	0.000	SCN4A	yes	yes	C	G	chr11:15474228
Hyperkalemic Periodic Paralysis, HYPP	HYPP	Arabian	0.000	SCN4A	yes	yes	C	G	chr11:15474228
Inesect bite hypersensitivity	IBH1	Other	0.395	NA	no	no	G	A	chrX:87097446
Inesect bite hypersensitivity	IBH1	Belgian	0.275	NA	no	no	G	A	chrX:87097446
Inesect bite hypersensitivity	IBH1	QH	0.274	NA	no	no	G	A	chrX:87097446
Inesect bite hypersensitivity	IBH1	Icelandic	0.559	NA	no	no	G	A	chrX:87097446
Inesect bite hypersensitivity	IBH1	Warmblood	0.471	NA	no	no	G	A	chrX:87097446
Inesect bite hypersensitivity	IBH1	FM	0.250	NA	no	no	G	A	chrX:87097446
Inesect bite hypersensitivity	IBH1	Shetland	0.173	NA	no	no	G	A	chrX:87097446
Inesect bite hypersensitivity	IBH1	TB	0.184	NA	no	no	G	A	chrX:87097446
Inesect bite hypersensitivity	IBH1	WP	0.375	NA	no	no	G	A	chrX:87097446
Inesect bite hypersensitivity	IBH1	Clydesdale	0.211	NA	no	no	G	A	chrX:87097446
Inesect bite hypersensitivity	IBH1	Morgan	0.227	NA	no	no	G	A	chrX:87097446

NA: Variants not present in this population

Supplementary table 6. Breed specific allele frequencies for the known variants

Inesect bite hypersensitivity	IBH1	STB	0.551	NA	no	no	G	A	chrX:87097446
Inesect bite hypersensitivity	IBH1	Arabian	0.303	NA	no	no	G	A	chrX:87097446
Inesect bite hypersensitivity	IBH2	Other	0.295	NA	no	no	A	G	chr1:154743141
Inesect bite hypersensitivity	IBH2	QH	0.415	NA	no	no	A	G	chr1:154743141
Inesect bite hypersensitivity	IBH2	TB	0.480	NA	no	no	A	G	chr1:154743141
Inesect bite hypersensitivity	IBH2	Icelandic	0.147	NA	no	no	A	G	chr1:154743141
Inesect bite hypersensitivity	IBH2	Warmblood	0.414	NA	no	no	A	G	chr1:154743141
Inesect bite hypersensitivity	IBH2	STB	0.449	NA	no	no	A	G	chr1:154743141
Inesect bite hypersensitivity	IBH2	Shetland	0.073	NA	no	no	A	G	chr1:154743141
Inesect bite hypersensitivity	IBH2	WP	0.400	NA	no	no	A	G	chr1:154743141
Inesect bite hypersensitivity	IBH2	Belgian	0.000	NA	no	no	A	G	chr1:154743141
Inesect bite hypersensitivity	IBH2	FM	0.483	NA	no	no	A	G	chr1:154743141
Inesect bite hypersensitivity	IBH2	Morgan	0.295	NA	no	no	A	G	chr1:154743141
Inesect bite hypersensitivity	IBH2	Arabian	0.421	NA	no	no	A	G	chr1:154743141
Inesect bite hypersensitivity	IBH2	Clydesdale	0.158	NA	no	no	A	G	chr1:154743141
Inesect bite hypersensitivity	IBH3	Shetland	0.627	NA	no	no	G	A	chr1:154538548
Inesect bite hypersensitivity	IBH3	QH	0.377	NA	no	no	G	A	chr1:154538548
Inesect bite hypersensitivity	IBH3	Icelandic	0.471	NA	no	no	G	A	chr1:154538548
Inesect bite hypersensitivity	IBH3	TB	0.217	NA	no	no	G	A	chr1:154538548
Inesect bite hypersensitivity	IBH3	Warmblood	0.300	NA	no	no	G	A	chr1:154538548
Inesect bite hypersensitivity	IBH3	WP	0.550	NA	no	no	G	A	chr1:154538548
Inesect bite hypersensitivity	IBH3	Morgan	0.545	NA	no	no	G	A	chr1:154538548
Inesect bite hypersensitivity	IBH3	Other	0.523	NA	no	no	G	A	chr1:154538548
Inesect bite hypersensitivity	IBH3	STB	0.602	NA	no	no	G	A	chr1:154538548
Inesect bite	IBH3	FM	0.633	NA	no	no	G	A	chr1:154538548

NA: Variants not present in this population

Supplementary table 6. Breed specific allele frequencies for the known variants

hypersensitivity									
Inesect bite hypersensitivity	IBH3	Arabian	0.526	NA	no	no	G	A	chr1:154538548
Inesect bite hypersensitivity	IBH3	Clydesdale	0.947	NA	no	no	G	A	chr1:154538548
Inesect bite hypersensitivity	IBH3	Belgian	0.950	NA	no	no	G	A	chr1:154538548
Incontinentia pigmenti	Inc_pig	WP	0.000	IKBKG	yes	yes	C	T	chrX:126898409
Incontinentia pigmenti	Inc_pig	Warmblood	0.014	IKBKG	yes	yes	C	T	chrX:126898409
Incontinentia pigmenti	Inc_pig	QH	0.000	IKBKG	yes	yes	C	T	chrX:126898409
Incontinentia pigmenti	Inc_pig	TB	0.000	IKBKG	yes	yes	C	T	chrX:126898409
Incontinentia pigmenti	Inc_pig	Icelandic	0.000	IKBKG	yes	yes	C	T	chrX:126898409
Incontinentia pigmenti	Inc_pig	Other	0.000	IKBKG	yes	yes	C	T	chrX:126898409
Incontinentia pigmenti	Inc_pig	STB	0.000	IKBKG	yes	yes	C	T	chrX:126898409
Incontinentia pigmenti	Inc_pig	Shetland	0.000	IKBKG	yes	yes	C	T	chrX:126898409
Incontinentia pigmenti	Inc_pig	Belgian	0.000	IKBKG	yes	yes	C	T	chrX:126898409
Incontinentia pigmenti	Inc_pig	FM	0.000	IKBKG	yes	yes	C	T	chrX:126898409
Incontinentia pigmenti	Inc_pig	Morgan	0.000	IKBKG	yes	yes	C	T	chrX:126898409
Incontinentia pigmenti	Inc_pig	Arabian	0.000	IKBKG	yes	yes	C	T	chrX:126898409
Incontinentia pigmenti	Inc_pig	Clydesdale	0.000	IKBKG	yes	yes	C	T	chrX:126898409
Epidermolysis bullosa, junctionalis, LAMC2-related	JEB	NA	NA	LAMC2	yes	yes	C	CC	chr5:17498175
Juvenile idiopathic epilepsy	JIE	TB	0.125	TRIM39-RPP21	yes	no	GTTCAGGG	TATCTTAAG	chr20:30454167
Juvenile idiopathic epilepsy	JIE	QH	0.387	TRIM39-RPP21	yes	no	GTTCAGGG	TATCTTAAG	chr20:30454167
Juvenile idiopathic epilepsy	JIE	Warmblood	0.186	TRIM39-RPP21	yes	no	GTTCAGGG	TATCTTAAG	chr20:30454167
Juvenile idiopathic epilepsy	JIE	STB	0.678	TRIM39-RPP21	yes	no	GTTCAGGG	TATCTTAAG	chr20:30454167
Juvenile idiopathic epilepsy	JIE	Shetland	0.445	TRIM39-RPP21	yes	no	GTTCAGGG	TATCTTAAG	chr20:30454167
Juvenile idiopathic epilepsy	JIE	WP	0.375	TRIM39-RPP21	yes	no	GTTCAGGG	TATCTTAAG	chr20:30454167
Juvenile idiopathic epilepsy	JIE	Other	0.282	TRIM39-RPP21	yes	no	GTTCAGGG	TATCTTAAG	chr20:30454167
Juvenile idiopathic epilepsy	JIE	FM	0.300	TRIM39-RPP21	yes	no	GTTCAGGG	TATCTTAAG	chr20:30454167
Juvenile idiopathic epilepsy	JIE	Morgan	0.523	TRIM39-RPP21	yes	no	GTTCAGGG	TATCTTAAG	chr20:30454167
Juvenile idiopathic epilepsy	JIE	Icelandic	0.324	TRIM39-RPP21	yes	no	GTTCAGGG	TATCTTAAG	chr20:30454167

NA: Variants not present in this population

Supplementary table 6. Breed specific allele frequencies for the known variants

Juvenile idiopathic epilepsy	JIE	Belgian	0.150	TRIM39-RPP21	yes	no	GTTCAGGG	TATCTTAAG	chr20:30454167
Juvenile idiopathic epilepsy	JIE	Arabian	0.645	TRIM39-RPP21	yes	no	GTTCAGGG	TATCTTAAG	chr20:30454167
Juvenile idiopathic epilepsy	JIE	Clydesdale	0.500	TRIM39-RPP21	yes	no	GTTCAGGG	TATCTTAAG	chr20:30454167
Lavender foal syndrome	LFS	NA	NA	MYO5A	yes	yes	C	*	chr1:139290592
Lordosis	Lord	NA	NA	NA	yes	no	C	T	chr20:42430846
Megacolon	LWFS	NA	NA	EDNRB	yes	yes	TC	AG	chr17:50502939
Coat colour, silver	MCOA	QH	0.000	PMEL	yes	yes	G	A	chr6:74569773
Coat colour, silver	MCOA	Shetland	0.018	PMEL	yes	yes	G	A	chr6:74569773
Coat colour, silver	MCOA	TB	0.000	PMEL	yes	yes	G	A	chr6:74569773
Coat colour, silver	MCOA	Warmblood	0.000	PMEL	yes	yes	G	A	chr6:74569773
Coat colour, silver	MCOA	FM	0.000	PMEL	yes	yes	G	A	chr6:74569773
Coat colour, silver	MCOA	Icelandic	0.088	PMEL	yes	yes	G	A	chr6:74569773
Coat colour, silver	MCOA	WP	0.000	PMEL	yes	yes	G	A	chr6:74569773
Coat colour, silver	MCOA	STB	0.000	PMEL	yes	yes	G	A	chr6:74569773
Coat colour, silver	MCOA	Arabian	0.000	PMEL	yes	yes	G	A	chr6:74569773
Coat colour, silver	MCOA	Morgan	0.000	PMEL	yes	yes	G	A	chr6:74569773
Coat colour, silver	MCOA	Other	0.000	PMEL	yes	yes	G	A	chr6:74569773
Coat colour, silver	MCOA	Belgian	0.000	PMEL	yes	yes	G	A	chr6:74569773
Coat colour, silver	MCOA	Clydesdale	0.000	PMEL	yes	yes	G	A	chr6:74569773
Melanoma	mel1	Warmblood	0.629	DPF3	yes	no	T	G	chr24:18379822
Melanoma	mel1	WP	0.450	DPF3	yes	no	T	G	chr24:18379822
Melanoma	mel1	TB	0.500	DPF3	yes	no	T	G	chr24:18379822
Melanoma	mel1	QH	0.448	DPF3	yes	no	T	G	chr24:18379822
Melanoma	mel1	Shetland	0.673	DPF3	yes	no	T	G	chr24:18379822
Melanoma	mel1	Morgan	0.432	DPF3	yes	no	T	G	chr24:18379822
Melanoma	mel1	Icelandic	0.529	DPF3	yes	no	T	G	chr24:18379822
Melanoma	mel1	Other	0.505	DPF3	yes	no	T	G	chr24:18379822
Melanoma	mel1	STB	0.847	DPF3	yes	no	T	G	chr24:18379822
Melanoma	mel1	Arabian	0.500	DPF3	yes	no	T	G	chr24:18379822
Melanoma	mel1	Clydesdale	0.737	DPF3	yes	no	T	G	chr24:18379822
Melanoma	mel1	Belgian	0.600	DPF3	yes	no	T	G	chr24:18379822
Melanoma	mel1	FM	0.850	DPF3	yes	no	T	G	chr24:18379822

NA: Variants not present in this population

Supplementary table 6. Breed specific allele frequencies for the known variants

Melanoma	mel2	Shetland	0.327	DPF3	yes	no	T	C	chr24:19216348
Melanoma	mel2	WP	0.600	DPF3	yes	no	T	C	chr24:19216348
Melanoma	mel2	TB	0.454	DPF3	yes	no	T	C	chr24:19216348
Melanoma	mel2	Warmblood	0.614	DPF3	yes	no	T	C	chr24:19216348
Melanoma	mel2	Belgian	0.700	DPF3	yes	no	T	C	chr24:19216348
Melanoma	mel2	Icelandic	0.676	DPF3	yes	no	T	C	chr24:19216348
Melanoma	mel2	Other	0.491	DPF3	yes	no	T	C	chr24:19216348
Melanoma	mel2	QH	0.406	DPF3	yes	no	T	C	chr24:19216348
Melanoma	mel2	FM	0.800	DPF3	yes	no	T	C	chr24:19216348
Melanoma	mel2	Morgan	0.591	DPF3	yes	no	T	C	chr24:19216348
Melanoma	mel2	STB	0.746	DPF3	yes	no	T	C	chr24:19216348
Melanoma	mel2	Arabian	0.447	DPF3	yes	no	T	C	chr24:19216348
Melanoma	mel2	Clydesdale	0.789	DPF3	yes	no	T	C	chr24:19216348
Melanoma	mel3	QH	0.404	DPF3	yes	no	C	T	chr24:18645181
Melanoma	mel3	STB	0.553	DPF3	yes	no	C	T	chr24:18645181
Melanoma	mel3	Other	0.439	DPF3	yes	no	C	T	chr24:18645181
Melanoma	mel3	Belgian	0.526	DPF3	yes	no	C	T	chr24:18645181
Melanoma	mel3	TB	0.459	DPF3	yes	no	C	T	chr24:18645181
Melanoma	mel3	Warmblood	0.533	DPF3	yes	no	C	T	chr24:18645181
Melanoma	mel3	Arabian	0.328	DPF3	yes	no	C	T	chr24:18645181
Melanoma	mel3	Clydesdale	0.053	DPF3	yes	no	C	T	chr24:18645181
Melanoma	mel3	Shetland	0.462	DPF3	yes	no	C	T	chr24:18645181
Melanoma	mel3	WP	0.361	DPF3	yes	no	C	T	chr24:18645181
Melanoma	mel3	FM	0.714	DPF3	yes	no	C	T	chr24:18645181
Melanoma	mel3	Icelandic	0.562	DPF3	yes	no	C	T	chr24:18645181
Melanoma	mel3	Morgan	0.619	DPF3	yes	no	C	T	chr24:18645181
Malignant hyperthermia	MH	TB	0.000	RYR1	yes	yes	C	G	chr10:9678680
Malignant hyperthermia	MH	Warmblood	0.000	RYR1	yes	yes	C	G	chr10:9678680
Malignant hyperthermia	MH	QH	0.014	RYR1	yes	yes	C	G	chr10:9678680
Malignant hyperthermia	MH	Shetland	0.000	RYR1	yes	yes	C	G	chr10:9678680
Malignant hyperthermia	MH	WP	0.000	RYR1	yes	yes	C	G	chr10:9678680
Malignant	MH	Icelandic	0.000	RYR1	yes	yes	C	G	chr10:9678680

NA: Variants not present in this population

Supplementary table 6. Breed specific allele frequencies for the known variants

hyperthermia									
Malignant hyperthermia	MH	Other	0.000	RYR1	yes	yes	C	G	chr10:9678680
Malignant hyperthermia	MH	STB	0.000	RYR1	yes	yes	C	G	chr10:9678680
Malignant hyperthermia	MH	Belgian	0.000	RYR1	yes	yes	C	G	chr10:9678680
Malignant hyperthermia	MH	FM	0.000	RYR1	yes	yes	C	G	chr10:9678680
Malignant hyperthermia	MH	Morgan	0.000	RYR1	yes	yes	C	G	chr10:9678680
Malignant hyperthermia	MH	Arabian	0.000	RYR1	yes	yes	C	G	chr10:9678680
Malignant hyperthermia	MH	Clydesdale	0.000	RYR1	yes	yes	C	G	chr10:9678680
Myositis, immune-mediated	MYHM	Shetland	0.000	MYH1	yes	yes	T	C	chr11:53345548
Myositis, immune-mediated	MYHM	TB	0.000	MYH1	yes	yes	T	C	chr11:53345548
Myositis, immune-mediated	MYHM	Warmblood	0.000	MYH1	yes	yes	T	C	chr11:53345548
Myositis, immune-mediated	MYHM	Belgian	0.050	MYH1	yes	yes	T	C	chr11:53345548
Myositis, immune-mediated	MYHM	WP	0.025	MYH1	yes	yes	T	C	chr11:53345548
Myositis, immune-mediated	MYHM	Other	0.005	MYH1	yes	yes	T	C	chr11:53345548
Myositis, immune-mediated	MYHM	QH	0.071	MYH1	yes	yes	T	C	chr11:53345548
Myositis, immune-mediated	MYHM	Arabian	0.000	MYH1	yes	yes	T	C	chr11:53345548
Myositis, immune-mediated	MYHM	Icelandic	0.000	MYH1	yes	yes	T	C	chr11:53345548
Myositis, immune-mediated	MYHM	FM	0.000	MYH1	yes	yes	T	C	chr11:53345548
Myositis, immune-mediated	MYHM	STB	0.000	MYH1	yes	yes	T	C	chr11:53345548
Myositis, immune-mediated	MYHM	Clydesdale	0.000	MYH1	yes	yes	T	C	chr11:53345548
Myositis, immune-mediated	MYHM	Morgan	0.000	MYH1	yes	yes	T	C	chr11:53345548
Myotonia	myotonia	NA	NA	CLCN1	yes	yes	A	C	chr4:96518592
Navicular disease	Nav1	QH	0.208	VSTM1	yes	no	G	A	chr10:23500725
Navicular disease	Nav1	Warmblood	0.300	VSTM1	yes	no	G	A	chr10:23500725
Navicular disease	Nav1	Icelandic	0.324	VSTM1	yes	no	G	A	chr10:23500725
Navicular disease	Nav1	TB	0.388	VSTM1	yes	no	G	A	chr10:23500725
Navicular disease	Nav1	WP	0.375	VSTM1	yes	no	G	A	chr10:23500725

NA: Variants not present in this population

Supplementary table 6. Breed specific allele frequencies for the known variants

Navicular disease	Nav1	FM	0.333	VSTM1	yes	no	G	A	chr10:23500725
Navicular disease	Nav1	Arabian	0.105	VSTM1	yes	no	G	A	chr10:23500725
Navicular disease	Nav1	Shetland	0.345	VSTM1	yes	no	G	A	chr10:23500725
Navicular disease	Nav1	Belgian	0.050	VSTM1	yes	no	G	A	chr10:23500725
Navicular disease	Nav1	Other	0.150	VSTM1	yes	no	G	A	chr10:23500725
Navicular disease	Nav1	STB	0.169	VSTM1	yes	no	G	A	chr10:23500725
Navicular disease	Nav1	Clydesdale	0.026	VSTM1	yes	no	G	A	chr10:23500725
Navicular disease	Nav1	Morgan	0.023	VSTM1	yes	no	G	A	chr10:23500725
Navicular disease	Nav2	Warmblood	0.029	IRF3	yes	no	C	T	chr10:19702335
Navicular disease	Nav2	QH	0.066	IRF3	yes	no	C	T	chr10:19702335
Navicular disease	Nav2	Icelandic	0.000	IRF3	yes	no	C	T	chr10:19702335
Navicular disease	Nav2	Belgian	0.000	IRF3	yes	no	C	T	chr10:19702335
Navicular disease	Nav2	Arabian	0.053	IRF3	yes	no	C	T	chr10:19702335
Navicular disease	Nav2	STB	0.297	IRF3	yes	no	C	T	chr10:19702335
Navicular disease	Nav2	Shetland	0.000	IRF3	yes	no	C	T	chr10:19702335
Navicular disease	Nav2	TB	0.013	IRF3	yes	no	C	T	chr10:19702335
Navicular disease	Nav2	Other	0.064	IRF3	yes	no	C	T	chr10:19702335
Navicular disease	Nav2	FM	0.000	IRF3	yes	no	C	T	chr10:19702335
Navicular disease	Nav2	WP	0.025	IRF3	yes	no	C	T	chr10:19702335
Navicular disease	Nav2	Clydesdale	0.026	IRF3	yes	no	C	T	chr10:19702335
Navicular disease	Nav2	Morgan	0.045	IRF3	yes	no	C	T	chr10:19702335
Navicular disease	Nav3	TB	0.026	IRF3	yes	no	C	T	chr10:19702353
Navicular disease	Nav3	Arabian	0.145	IRF3	yes	no	C	T	chr10:19702353
Navicular disease	Nav3	Warmblood	0.071	IRF3	yes	no	C	T	chr10:19702353
Navicular disease	Nav3	Other	0.032	IRF3	yes	no	C	T	chr10:19702353
Navicular disease	Nav3	QH	0.047	IRF3	yes	no	C	T	chr10:19702353
Navicular disease	Nav3	Belgian	0.025	IRF3	yes	no	C	T	chr10:19702353
Navicular disease	Nav3	Icelandic	0.000	IRF3	yes	no	C	T	chr10:19702353
Navicular disease	Nav3	Clydesdale	0.000	IRF3	yes	no	C	T	chr10:19702353
Navicular disease	Nav3	FM	0.017	IRF3	yes	no	C	T	chr10:19702353
Navicular disease	Nav3	Shetland	0.018	IRF3	yes	no	C	T	chr10:19702353
Navicular disease	Nav3	WP	0.025	IRF3	yes	no	C	T	chr10:19702353
Navicular disease	Nav3	Morgan	0.182	IRF3	yes	no	C	T	chr10:19702353
Navicular disease	Nav3	STB	0.085	IRF3	yes	no	C	T	chr10:19702353

NA: Variants not present in this population

Supplementary table 6. Breed specific allele frequencies for the known variants

Naked foal syndrome	NFS	NA	NA	ST14	yes	yes	G	T	chr7:39710628
Osteochondrosis	OC1	Belgian	0.725	FRZB	yes	no	C	T	chr18:60559998
Osteochondrosis	OC1	QH	0.873	FRZB	yes	no	C	T	chr18:60559998
Osteochondrosis	OC1	FM	0.367	FRZB	yes	no	C	T	chr18:60559998
Osteochondrosis	OC1	Icelandic	0.765	FRZB	yes	no	C	T	chr18:60559998
Osteochondrosis	OC1	TB	0.809	FRZB	yes	no	C	T	chr18:60559998
Osteochondrosis	OC1	Arabian	0.816	FRZB	yes	no	C	T	chr18:60559998
Osteochondrosis	OC1	Shetland	0.609	FRZB	yes	no	C	T	chr18:60559998
Osteochondrosis	OC1	WP	0.500	FRZB	yes	no	C	T	chr18:60559998
Osteochondrosis	OC1	Other	0.755	FRZB	yes	no	C	T	chr18:60559998
Osteochondrosis	OC1	Clydesdale	0.632	FRZB	yes	no	C	T	chr18:60559998
Osteochondrosis	OC1	Morgan	0.591	FRZB	yes	no	C	T	chr18:60559998
Osteochondrosis	OC1	Warmblood	0.771	FRZB	yes	no	C	T	chr18:60559998
Osteochondrosis	OC1	STB	0.924	FRZB	yes	no	C	T	chr18:60559998
Osteochondrosis	OC10	Clydesdale	0.132	CTBBD2	yes	no	A	T	chr21:49916703
Osteochondrosis	OC10	Belgian	0.125	CTBBD2	yes	no	A	T	chr21:49916703
Osteochondrosis	OC10	QH	0.245	CTBBD2	yes	no	A	T	chr21:49916703
Osteochondrosis	OC10	Shetland	0.173	CTBBD2	yes	no	A	T	chr21:49916703
Osteochondrosis	OC10	FM	0.100	CTBBD2	yes	no	A	T	chr21:49916703
Osteochondrosis	OC10	Arabian	0.158	CTBBD2	yes	no	A	T	chr21:49916703
Osteochondrosis	OC10	Morgan	0.205	CTBBD2	yes	no	A	T	chr21:49916703
Osteochondrosis	OC10	TB	0.434	CTBBD2	yes	no	A	T	chr21:49916703
Osteochondrosis	OC10	Icelandic	0.206	CTBBD2	yes	no	A	T	chr21:49916703
Osteochondrosis	OC10	WP	0.200	CTBBD2	yes	no	A	T	chr21:49916703
Osteochondrosis	OC10	Other	0.191	CTBBD2	yes	no	A	T	chr21:49916703
Osteochondrosis	OC10	Warmblood	0.243	CTBBD2	yes	no	A	T	chr21:49916703
Osteochondrosis	OC10	STB	0.364	CTBBD2	yes	no	A	T	chr21:49916703
Osteochondrosis	OC11	Icelandic	0.265	PDSS2	yes	no	G	A	chr10:58342337
Osteochondrosis	OC11	FM	0.183	PDSS2	yes	no	G	A	chr10:58342337
Osteochondrosis	OC11	Morgan	0.068	PDSS2	yes	no	G	A	chr10:58342337
Osteochondrosis	OC11	TB	0.026	PDSS2	yes	no	G	A	chr10:58342337
Osteochondrosis	OC11	Belgian	0.150	PDSS2	yes	no	G	A	chr10:58342337
Osteochondrosis	OC11	WP	0.100	PDSS2	yes	no	G	A	chr10:58342337
Osteochondrosis	OC11	Other	0.132	PDSS2	yes	no	G	A	chr10:58342337

NA: Variants not present in this population

Supplementary table 6. Breed specific allele frequencies for the known variants

Osteochondrosis	OC11	Arabian	0.000	PDSS2	yes	no	G	A	chr10:58342337
Osteochondrosis	OC11	Shetland	0.373	PDSS2	yes	no	G	A	chr10:58342337
Osteochondrosis	OC11	STB	0.119	PDSS2	yes	no	G	A	chr10:58342337
Osteochondrosis	OC11	Warmblood	0.157	PDSS2	yes	no	G	A	chr10:58342337
Osteochondrosis	OC11	QH	0.142	PDSS2	yes	no	G	A	chr10:58342337
Osteochondrosis	OC11	Clydesdale	0.000	PDSS2	yes	no	G	A	chr10:58342337
Osteochondrosis	OC12	Other	0.686	CLCA2	yes	no	T	G	chr5:74476969
Osteochondrosis	OC12	QH	0.698	CLCA2	yes	no	T	G	chr5:74476969
Osteochondrosis	OC12	Belgian	0.675	CLCA2	yes	no	T	G	chr5:74476969
Osteochondrosis	OC12	Icelandic	0.941	CLCA2	yes	no	T	G	chr5:74476969
Osteochondrosis	OC12	FM	0.633	CLCA2	yes	no	T	G	chr5:74476969
Osteochondrosis	OC12	STB	0.831	CLCA2	yes	no	T	G	chr5:74476969
Osteochondrosis	OC12	Arabian	0.921	CLCA2	yes	no	T	G	chr5:74476969
Osteochondrosis	OC12	Morgan	0.841	CLCA2	yes	no	T	G	chr5:74476969
Osteochondrosis	OC12	TB	0.632	CLCA2	yes	no	T	G	chr5:74476969
Osteochondrosis	OC12	Clydesdale	0.842	CLCA2	yes	no	T	G	chr5:74476969
Osteochondrosis	OC12	Shetland	0.709	CLCA2	yes	no	T	G	chr5:74476969
Osteochondrosis	OC12	Warmblood	0.700	CLCA2	yes	no	T	G	chr5:74476969
Osteochondrosis	OC12	WP	0.650	CLCA2	yes	no	T	G	chr5:74476969
Osteochondrosis	OC13	Other	0.027	WDR63	yes	no	T	C	chr5:75652047
Osteochondrosis	OC13	QH	0.019	WDR63	yes	no	T	C	chr5:75652047
Osteochondrosis	OC13	Arabian	0.000	WDR63	yes	no	T	C	chr5:75652047
Osteochondrosis	OC13	Belgian	0.075	WDR63	yes	no	T	C	chr5:75652047
Osteochondrosis	OC13	STB	0.186	WDR63	yes	no	T	C	chr5:75652047
Osteochondrosis	OC13	Shetland	0.000	WDR63	yes	no	T	C	chr5:75652047
Osteochondrosis	OC13	FM	0.083	WDR63	yes	no	T	C	chr5:75652047
Osteochondrosis	OC13	Clydesdale	0.026	WDR63	yes	no	T	C	chr5:75652047
Osteochondrosis	OC13	Morgan	0.000	WDR63	yes	no	T	C	chr5:75652047
Osteochondrosis	OC13	TB	0.046	WDR63	yes	no	T	C	chr5:75652047
Osteochondrosis	OC13	Icelandic	0.059	WDR63	yes	no	T	C	chr5:75652047
Osteochondrosis	OC13	WP	0.025	WDR63	yes	no	T	C	chr5:75652047
Osteochondrosis	OC13	Warmblood	0.043	WDR63	yes	no	T	C	chr5:75652047
Osteochondrosis	OC14	QH	0.212	SLC27A2	yes	no	C	T	chr1:141005757
Osteochondrosis	OC14	STB	0.237	SLC27A2	yes	no	C	T	chr1:141005757

NA: Variants not present in this population

Supplementary table 6. Breed specific allele frequencies for the known variants

Osteochondrosis	OC14	Icelandic	0.324	SLC27A2	yes	no	C	T	chr1:141005757
Osteochondrosis	OC14	Morgan	0.159	SLC27A2	yes	no	C	T	chr1:141005757
Osteochondrosis	OC14	Arabian	0.053	SLC27A2	yes	no	C	T	chr1:141005757
Osteochondrosis	OC14	Belgian	0.350	SLC27A2	yes	no	C	T	chr1:141005757
Osteochondrosis	OC14	FM	0.467	SLC27A2	yes	no	C	T	chr1:141005757
Osteochondrosis	OC14	Shetland	0.073	SLC27A2	yes	no	C	T	chr1:141005757
Osteochondrosis	OC14	Clydesdale	0.237	SLC27A2	yes	no	C	T	chr1:141005757
Osteochondrosis	OC14	Warmblood	0.086	SLC27A2	yes	no	C	T	chr1:141005757
Osteochondrosis	OC14	Other	0.155	SLC27A2	yes	no	C	T	chr1:141005757
Osteochondrosis	OC14	TB	0.020	SLC27A2	yes	no	C	T	chr1:141005757
Osteochondrosis	OC14	WP	0.125	SLC27A2	yes	no	C	T	chr1:141005757
Osteochondrosis	OC15	Shetland	0.018	QRSL1	yes	no	G	T	chr10:57949860
Osteochondrosis	OC15	Arabian	0.237	QRSL1	yes	no	G	T	chr10:57949860
Osteochondrosis	OC15	Icelandic	0.382	QRSL1	yes	no	G	T	chr10:57949860
Osteochondrosis	OC15	Other	0.218	QRSL1	yes	no	G	T	chr10:57949860
Osteochondrosis	OC15	TB	0.000	QRSL1	yes	no	G	T	chr10:57949860
Osteochondrosis	OC15	Clydesdale	0.289	QRSL1	yes	no	G	T	chr10:57949860
Osteochondrosis	OC15	Morgan	0.250	QRSL1	yes	no	G	T	chr10:57949860
Osteochondrosis	OC15	Belgian	0.050	QRSL1	yes	no	G	T	chr10:57949860
Osteochondrosis	OC15	FM	0.317	QRSL1	yes	no	G	T	chr10:57949860
Osteochondrosis	OC15	Warmblood	0.029	QRSL1	yes	no	G	T	chr10:57949860
Osteochondrosis	OC15	QH	0.151	QRSL1	yes	no	G	T	chr10:57949860
Osteochondrosis	OC15	WP	0.150	QRSL1	yes	no	G	T	chr10:57949860
Osteochondrosis	OC15	STB	0.144	QRSL1	yes	no	G	T	chr10:57949860
Osteochondrosis	OC16	QH	0.108	SPATA24	yes	no	C	T	chr14:36580417
Osteochondrosis	OC16	TB	0.125	SPATA24	yes	no	C	T	chr14:36580417
Osteochondrosis	OC16	FM	0.117	SPATA24	yes	no	C	T	chr14:36580417
Osteochondrosis	OC16	Belgian	0.050	SPATA24	yes	no	C	T	chr14:36580417
Osteochondrosis	OC16	Icelandic	0.441	SPATA24	yes	no	C	T	chr14:36580417
Osteochondrosis	OC16	Warmblood	0.114	SPATA24	yes	no	C	T	chr14:36580417
Osteochondrosis	OC16	Shetland	0.191	SPATA24	yes	no	C	T	chr14:36580417
Osteochondrosis	OC16	Arabian	0.066	SPATA24	yes	no	C	T	chr14:36580417
Osteochondrosis	OC16	Clydesdale	0.026	SPATA24	yes	no	C	T	chr14:36580417
Osteochondrosis	OC16	Morgan	0.227	SPATA24	yes	no	C	T	chr14:36580417

NA: Variants not present in this population

Supplementary table 6. Breed specific allele frequencies for the known variants

Osteochondrosis	OC16	WP	0.175	SPATA24	yes	no	C	T	chr14:36580417
Osteochondrosis	OC16	STB	0.246	SPATA24	yes	no	C	T	chr14:36580417
Osteochondrosis	OC16	Other	0.155	SPATA24	yes	no	C	T	chr14:36580417
Osteochondrosis	OC17	QH	0.392	KLF3	yes	no	C	T	chr3:90617505
Osteochondrosis	OC17	Belgian	0.400	KLF3	yes	no	C	T	chr3:90617505
Osteochondrosis	OC17	Shetland	0.391	KLF3	yes	no	C	T	chr3:90617505
Osteochondrosis	OC17	Other	0.441	KLF3	yes	no	C	T	chr3:90617505
Osteochondrosis	OC17	STB	0.610	KLF3	yes	no	C	T	chr3:90617505
Osteochondrosis	OC17	FM	0.417	KLF3	yes	no	C	T	chr3:90617505
Osteochondrosis	OC17	Icelandic	0.676	KLF3	yes	no	C	T	chr3:90617505
Osteochondrosis	OC17	Arabian	0.487	KLF3	yes	no	C	T	chr3:90617505
Osteochondrosis	OC17	Clydesdale	0.789	KLF3	yes	no	C	T	chr3:90617505
Osteochondrosis	OC17	Warmblood	0.357	KLF3	yes	no	C	T	chr3:90617505
Osteochondrosis	OC17	TB	0.368	KLF3	yes	no	C	T	chr3:90617505
Osteochondrosis	OC17	Morgan	0.636	KLF3	yes	no	C	T	chr3:90617505
Osteochondrosis	OC17	WP	0.375	KLF3	yes	no	C	T	chr3:90617505
Osteochondrosis	OC18	Icelandic	0.000	LCORL	yes	no	T	C	chr3:107374136
Osteochondrosis	OC18	STB	0.169	LCORL	yes	no	T	C	chr3:107374136
Osteochondrosis	OC18	FM	0.117	LCORL	yes	no	T	C	chr3:107374136
Osteochondrosis	OC18	Shetland	0.018	LCORL	yes	no	T	C	chr3:107374136
Osteochondrosis	OC18	QH	0.009	LCORL	yes	no	T	C	chr3:107374136
Osteochondrosis	OC18	Belgian	0.950	LCORL	yes	no	T	C	chr3:107374136
Osteochondrosis	OC18	Arabian	0.000	LCORL	yes	no	T	C	chr3:107374136
Osteochondrosis	OC18	Morgan	0.000	LCORL	yes	no	T	C	chr3:107374136
Osteochondrosis	OC18	TB	0.145	LCORL	yes	no	T	C	chr3:107374136
Osteochondrosis	OC18	WP	0.000	LCORL	yes	no	T	C	chr3:107374136
Osteochondrosis	OC18	Clydesdale	0.947	LCORL	yes	no	T	C	chr3:107374136
Osteochondrosis	OC18	Warmblood	0.529	LCORL	yes	no	T	C	chr3:107374136
Osteochondrosis	OC18	Other	0.155	LCORL	yes	no	T	C	chr3:107374136
Osteochondrosis	OC19	Other	0.136	NA	yes	no	C	T	chr10:81566455
Osteochondrosis	OC19	Icelandic	0.059	NA	yes	no	C	T	chr10:81566455
Osteochondrosis	OC19	Shetland	0.045	NA	yes	no	C	T	chr10:81566455
Osteochondrosis	OC19	QH	0.212	NA	yes	no	C	T	chr10:81566455
Osteochondrosis	OC19	Morgan	0.205	NA	yes	no	C	T	chr10:81566455

NA: Variants not present in this population

Supplementary table 6. Breed specific allele frequencies for the known variants

Osteochondrosis	OC19	WP	0.350	NA	yes	no	C	T	chr10:81566455
Osteochondrosis	OC19	TB	0.270	NA	yes	no	C	T	chr10:81566455
Osteochondrosis	OC19	Warmblood	0.129	NA	yes	no	C	T	chr10:81566455
Osteochondrosis	OC19	Belgian	0.225	NA	yes	no	C	T	chr10:81566455
Osteochondrosis	OC19	FM	0.450	NA	yes	no	C	T	chr10:81566455
Osteochondrosis	OC19	STB	0.203	NA	yes	no	C	T	chr10:81566455
Osteochondrosis	OC19	Arabian	0.197	NA	yes	no	C	T	chr10:81566455
Osteochondrosis	OC19	Clydesdale	0.053	NA	yes	no	C	T	chr10:81566455
Osteochondrosis	OC2	STB	0.127	HYAL1	yes	no	A	C	chr16:38503653
Osteochondrosis	OC2	QH	0.080	HYAL1	yes	no	A	C	chr16:38503653
Osteochondrosis	OC2	Morgan	0.114	HYAL1	yes	no	A	C	chr16:38503653
Osteochondrosis	OC2	Warmblood	0.114	HYAL1	yes	no	A	C	chr16:38503653
Osteochondrosis	OC2	Shetland	0.045	HYAL1	yes	no	A	C	chr16:38503653
Osteochondrosis	OC2	Icelandic	0.029	HYAL1	yes	no	A	C	chr16:38503653
Osteochondrosis	OC2	Arabian	0.053	HYAL1	yes	no	A	C	chr16:38503653
Osteochondrosis	OC2	Belgian	0.025	HYAL1	yes	no	A	C	chr16:38503653
Osteochondrosis	OC2	WP	0.275	HYAL1	yes	no	A	C	chr16:38503653
Osteochondrosis	OC2	TB	0.125	HYAL1	yes	no	A	C	chr16:38503653
Osteochondrosis	OC2	Clydesdale	0.000	HYAL1	yes	no	A	C	chr16:38503653
Osteochondrosis	OC2	FM	0.050	HYAL1	yes	no	A	C	chr16:38503653
Osteochondrosis	OC2	Other	0.123	HYAL1	yes	no	A	C	chr16:38503653
Osteochondrosis	OC20	Warmblood	0.500	CPVL	yes	no	C	G	chr4:60079060
Osteochondrosis	OC20	Belgian	0.125	CPVL	yes	no	C	G	chr4:60079060
Osteochondrosis	OC20	Arabian	0.474	CPVL	yes	no	C	G	chr4:60079060
Osteochondrosis	OC20	Clydesdale	0.132	CPVL	yes	no	C	G	chr4:60079060
Osteochondrosis	OC20	TB	0.408	CPVL	yes	no	C	G	chr4:60079060
Osteochondrosis	OC20	QH	0.472	CPVL	yes	no	C	G	chr4:60079060
Osteochondrosis	OC20	FM	0.417	CPVL	yes	no	C	G	chr4:60079060
Osteochondrosis	OC20	Morgan	0.545	CPVL	yes	no	C	G	chr4:60079060
Osteochondrosis	OC20	Other	0.273	CPVL	yes	no	C	G	chr4:60079060
Osteochondrosis	OC20	Shetland	0.364	CPVL	yes	no	C	G	chr4:60079060
Osteochondrosis	OC20	Icelandic	0.441	CPVL	yes	no	C	G	chr4:60079060
Osteochondrosis	OC20	STB	0.144	CPVL	yes	no	C	G	chr4:60079060
Osteochondrosis	OC20	WP	0.400	CPVL	yes	no	C	G	chr4:60079060

NA: Variants not present in this population

Supplementary table 6. Breed specific allele frequencies for the known variants

Osteochondrosis	OC21	Shetland	0.109	MATN1	yes	no	T	C	chr2:25763626
Osteochondrosis	OC21	QH	0.335	MATN1	yes	no	T	C	chr2:25763626
Osteochondrosis	OC21	WP	0.375	MATN1	yes	no	T	C	chr2:25763626
Osteochondrosis	OC21	TB	0.197	MATN1	yes	no	T	C	chr2:25763626
Osteochondrosis	OC21	STB	0.398	MATN1	yes	no	T	C	chr2:25763626
Osteochondrosis	OC21	Belgian	0.275	MATN1	yes	no	T	C	chr2:25763626
Osteochondrosis	OC21	Warmblood	0.186	MATN1	yes	no	T	C	chr2:25763626
Osteochondrosis	OC21	Icelandic	0.118	MATN1	yes	no	T	C	chr2:25763626
Osteochondrosis	OC21	Clydesdale	0.079	MATN1	yes	no	T	C	chr2:25763626
Osteochondrosis	OC21	Arabian	0.263	MATN1	yes	no	T	C	chr2:25763626
Osteochondrosis	OC21	Morgan	0.045	MATN1	yes	no	T	C	chr2:25763626
Osteochondrosis	OC21	Other	0.168	MATN1	yes	no	T	C	chr2:25763626
Osteochondrosis	OC21	FM	0.267	MATN1	yes	no	T	C	chr2:25763626
Osteochondrosis	OC23	QH	0.613	MCTP1	yes	no	A	G	chr14:73883434
Osteochondrosis	OC23	Shetland	0.582	MCTP1	yes	no	A	G	chr14:73883434
Osteochondrosis	OC23	Arabian	0.605	MCTP1	yes	no	A	G	chr14:73883434
Osteochondrosis	OC23	FM	0.683	MCTP1	yes	no	A	G	chr14:73883434
Osteochondrosis	OC23	Belgian	0.625	MCTP1	yes	no	A	G	chr14:73883434
Osteochondrosis	OC23	TB	0.362	MCTP1	yes	no	A	G	chr14:73883434
Osteochondrosis	OC23	Clydesdale	0.211	MCTP1	yes	no	A	G	chr14:73883434
Osteochondrosis	OC23	Morgan	0.727	MCTP1	yes	no	A	G	chr14:73883434
Osteochondrosis	OC23	STB	0.314	MCTP1	yes	no	A	G	chr14:73883434
Osteochondrosis	OC23	Warmblood	0.500	MCTP1	yes	no	A	G	chr14:73883434
Osteochondrosis	OC23	Other	0.609	MCTP1	yes	no	A	G	chr14:73883434
Osteochondrosis	OC23	Icelandic	0.647	MCTP1	yes	no	A	G	chr14:73883434
Osteochondrosis	OC23	WP	0.875	MCTP1	yes	no	A	G	chr14:73883434
Osteochondrosis	OC25	NA	NA	XIRP2	yes	no	G	C	chr18:47135212
Osteochondrosis	OC26	QH	0.099	GABRA6	yes	no	T	A	chr14:17329705
Osteochondrosis	OC26	Belgian	0.100	GABRA6	yes	no	T	A	chr14:17329705
Osteochondrosis	OC26	STB	0.212	GABRA6	yes	no	T	A	chr14:17329705
Osteochondrosis	OC26	TB	0.033	GABRA6	yes	no	T	A	chr14:17329705
Osteochondrosis	OC26	Icelandic	0.147	GABRA6	yes	no	T	A	chr14:17329705
Osteochondrosis	OC26	Arabian	0.158	GABRA6	yes	no	T	A	chr14:17329705
Osteochondrosis	OC26	Clydesdale	0.211	GABRA6	yes	no	T	A	chr14:17329705

NA: Variants not present in this population

Supplementary table 6. Breed specific allele frequencies for the known variants

Osteochondrosis	OC26	FM	0.017	GABRA6	yes	no	T	A	chr14:17329705
Osteochondrosis	OC26	WP	0.075	GABRA6	yes	no	T	A	chr14:17329705
Osteochondrosis	OC26	Other	0.123	GABRA6	yes	no	T	A	chr14:17329705
Osteochondrosis	OC26	Shetland	0.073	GABRA6	yes	no	T	A	chr14:17329705
Osteochondrosis	OC26	Warmblood	0.200	GABRA6	yes	no	T	A	chr14:17329705
Osteochondrosis	OC26	Morgan	0.227	GABRA6	yes	no	T	A	chr14:17329705
Osteochondrosis	OC27	QH	0.000	JAKMIP1	yes	no	T	G	chr3:115918112
Osteochondrosis	OC27	Belgian	0.000	JAKMIP1	yes	no	T	G	chr3:115918112
Osteochondrosis	OC27	FM	0.000	JAKMIP1	yes	no	T	G	chr3:115918112
Osteochondrosis	OC27	Arabian	0.000	JAKMIP1	yes	no	T	G	chr3:115918112
Osteochondrosis	OC27	Icelandic	0.000	JAKMIP1	yes	no	T	G	chr3:115918112
Osteochondrosis	OC27	WP	0.000	JAKMIP1	yes	no	T	G	chr3:115918112
Osteochondrosis	OC27	Shetland	0.000	JAKMIP1	yes	no	T	G	chr3:115918112
Osteochondrosis	OC27	TB	0.007	JAKMIP1	yes	no	T	G	chr3:115918112
Osteochondrosis	OC27	Clydesdale	0.000	JAKMIP1	yes	no	T	G	chr3:115918112
Osteochondrosis	OC27	Morgan	0.000	JAKMIP1	yes	no	T	G	chr3:115918112
Osteochondrosis	OC27	Other	0.000	JAKMIP1	yes	no	T	G	chr3:115918112
Osteochondrosis	OC27	Warmblood	0.000	JAKMIP1	yes	no	T	G	chr3:115918112
Osteochondrosis	OC27	STB	0.000	JAKMIP1	yes	no	T	G	chr3:115918112
Osteochondrosis	OC28	NA	NA	RTN3	yes	no	C	G	chr12:27646686
Osteochondrosis	OC29	NA	NA	NA	yes	no	C	G	chr18:13798499
Osteochondrosis	OC3	TB	0.901	COL5A2	yes	no	C	T	chr18:65672605
Osteochondrosis	OC3	Arabian	0.908	COL5A2	yes	no	C	T	chr18:65672605
Osteochondrosis	OC3	Belgian	0.900	COL5A2	yes	no	C	T	chr18:65672605
Osteochondrosis	OC3	FM	1.000	COL5A2	yes	no	C	T	chr18:65672605
Osteochondrosis	OC3	Other	0.709	COL5A2	yes	no	C	T	chr18:65672605
Osteochondrosis	OC3	QH	0.877	COL5A2	yes	no	C	T	chr18:65672605
Osteochondrosis	OC3	Shetland	0.700	COL5A2	yes	no	C	T	chr18:65672605
Osteochondrosis	OC3	Icelandic	0.941	COL5A2	yes	no	C	T	chr18:65672605
Osteochondrosis	OC3	Clydesdale	0.921	COL5A2	yes	no	C	T	chr18:65672605
Osteochondrosis	OC3	Morgan	0.886	COL5A2	yes	no	C	T	chr18:65672605
Osteochondrosis	OC3	STB	0.864	COL5A2	yes	no	C	T	chr18:65672605
Osteochondrosis	OC3	WP	0.975	COL5A2	yes	no	C	T	chr18:65672605
Osteochondrosis	OC3	Warmblood	0.729	COL5A2	yes	no	C	T	chr18:65672605

NA: Variants not present in this population

Supplementary table 6. Breed specific allele frequencies for the known variants

Osteochondrosis	OC4	Arabian	0.000	PROM1	yes	no	C	T	chr3:107352237
Osteochondrosis	OC4	Belgian	0.000	PROM1	yes	no	C	T	chr3:107352237
Osteochondrosis	OC4	Icelandic	0.088	PROM1	yes	no	C	T	chr3:107352237
Osteochondrosis	OC4	QH	0.052	PROM1	yes	no	C	T	chr3:107352237
Osteochondrosis	OC4	TB	0.026	PROM1	yes	no	C	T	chr3:107352237
Osteochondrosis	OC4	Clydesdale	0.000	PROM1	yes	no	C	T	chr3:107352237
Osteochondrosis	OC4	FM	0.000	PROM1	yes	no	C	T	chr3:107352237
Osteochondrosis	OC4	Morgan	0.227	PROM1	yes	no	C	T	chr3:107352237
Osteochondrosis	OC4	Other	0.045	PROM1	yes	no	C	T	chr3:107352237
Osteochondrosis	OC4	Warmblood	0.071	PROM1	yes	no	C	T	chr3:107352237
Osteochondrosis	OC4	Shetland	0.227	PROM1	yes	no	C	T	chr3:107352237
Osteochondrosis	OC4	WP	0.150	PROM1	yes	no	C	T	chr3:107352237
Osteochondrosis	OC4	STB	0.008	PROM1	yes	no	C	T	chr3:107352237
Osteochondrosis	OC5	Belgian	0.175	ATP8B4	yes	no	A	G	chr1:141266651
Osteochondrosis	OC5	Icelandic	0.147	ATP8B4	yes	no	A	G	chr1:141266651
Osteochondrosis	OC5	Arabian	0.000	ATP8B4	yes	no	A	G	chr1:141266651
Osteochondrosis	OC5	QH	0.028	ATP8B4	yes	no	A	G	chr1:141266651
Osteochondrosis	OC5	FM	0.117	ATP8B4	yes	no	A	G	chr1:141266651
Osteochondrosis	OC5	Clydesdale	0.553	ATP8B4	yes	no	A	G	chr1:141266651
Osteochondrosis	OC5	Other	0.068	ATP8B4	yes	no	A	G	chr1:141266651
Osteochondrosis	OC5	STB	0.246	ATP8B4	yes	no	A	G	chr1:141266651
Osteochondrosis	OC5	Shetland	0.091	ATP8B4	yes	no	A	G	chr1:141266651
Osteochondrosis	OC5	TB	0.000	ATP8B4	yes	no	A	G	chr1:141266651
Osteochondrosis	OC5	Morgan	0.045	ATP8B4	yes	no	A	G	chr1:141266651
Osteochondrosis	OC5	WP	0.025	ATP8B4	yes	no	A	G	chr1:141266651
Osteochondrosis	OC5	Warmblood	0.057	ATP8B4	yes	no	A	G	chr1:141266651
Osteochondrosis	OC6	Arabian	0.158	TRPM7	yes	no	A	T	chr1:140746974
Osteochondrosis	OC6	Belgian	0.125	TRPM7	yes	no	A	T	chr1:140746974
Osteochondrosis	OC6	QH	0.090	TRPM7	yes	no	A	T	chr1:140746974
Osteochondrosis	OC6	Clydesdale	0.289	TRPM7	yes	no	A	T	chr1:140746974
Osteochondrosis	OC6	FM	0.533	TRPM7	yes	no	A	T	chr1:140746974
Osteochondrosis	OC6	Icelandic	0.029	TRPM7	yes	no	A	T	chr1:140746974
Osteochondrosis	OC6	Other	0.205	TRPM7	yes	no	A	T	chr1:140746974
Osteochondrosis	OC6	TB	0.007	TRPM7	yes	no	A	T	chr1:140746974

NA: Variants not present in this population

Supplementary table 6. Breed specific allele frequencies for the known variants

Osteochondrosis	OC6	Shetland	0.273	TRPM7	yes	no	A	T	chr1:140746974
Osteochondrosis	OC6	Morgan	0.114	TRPM7	yes	no	A	T	chr1:140746974
Osteochondrosis	OC6	Warmblood	0.071	TRPM7	yes	no	A	T	chr1:140746974
Osteochondrosis	OC6	STB	0.220	TRPM7	yes	no	A	T	chr1:140746974
Osteochondrosis	OC6	WP	0.400	TRPM7	yes	no	A	T	chr1:140746974
Osteochondrosis	OC7	Belgian	0.050	HMMR	yes	no	T	C	chr14:16064796
Osteochondrosis	OC7	Icelandic	0.088	HMMR	yes	no	T	C	chr14:16064796
Osteochondrosis	OC7	Arabian	0.053	HMMR	yes	no	T	C	chr14:16064796
Osteochondrosis	OC7	Clydesdale	0.000	HMMR	yes	no	T	C	chr14:16064796
Osteochondrosis	OC7	FM	0.250	HMMR	yes	no	T	C	chr14:16064796
Osteochondrosis	OC7	STB	0.246	HMMR	yes	no	T	C	chr14:16064796
Osteochondrosis	OC7	Other	0.168	HMMR	yes	no	T	C	chr14:16064796
Osteochondrosis	OC7	QH	0.160	HMMR	yes	no	T	C	chr14:16064796
Osteochondrosis	OC7	Morgan	0.068	HMMR	yes	no	T	C	chr14:16064796
Osteochondrosis	OC7	TB	0.138	HMMR	yes	no	T	C	chr14:16064796
Osteochondrosis	OC7	WP	0.000	HMMR	yes	no	T	C	chr14:16064796
Osteochondrosis	OC7	Shetland	0.009	HMMR	yes	no	T	C	chr14:16064796
Osteochondrosis	OC7	Warmblood	0.229	HMMR	yes	no	T	C	chr14:16064796
Osteochondrosis	OC8	Arabian	0.053	NUDCD2	yes	no	A	G	chr14:16074764
Osteochondrosis	OC8	Icelandic	0.147	NUDCD2	yes	no	A	G	chr14:16074764
Osteochondrosis	OC8	TB	0.138	NUDCD2	yes	no	A	G	chr14:16074764
Osteochondrosis	OC8	Belgian	0.050	NUDCD2	yes	no	A	G	chr14:16074764
Osteochondrosis	OC8	FM	0.250	NUDCD2	yes	no	A	G	chr14:16074764
Osteochondrosis	OC8	QH	0.142	NUDCD2	yes	no	A	G	chr14:16074764
Osteochondrosis	OC8	Other	0.191	NUDCD2	yes	no	A	G	chr14:16074764
Osteochondrosis	OC8	Clydesdale	0.000	NUDCD2	yes	no	A	G	chr14:16074764
Osteochondrosis	OC8	Shetland	0.009	NUDCD2	yes	no	A	G	chr14:16074764
Osteochondrosis	OC8	Morgan	0.068	NUDCD2	yes	no	A	G	chr14:16074764
Osteochondrosis	OC8	Warmblood	0.229	NUDCD2	yes	no	A	G	chr14:16074764
Osteochondrosis	OC8	STB	0.254	NUDCD2	yes	no	A	G	chr14:16074764
Osteochondrosis	OC8	WP	0.000	NUDCD2	yes	no	A	G	chr14:16074764
Osteochondrosis	OC9	Other	0.355	ATXN7L1	yes	no	G	A	chr4:5923681
Osteochondrosis	OC9	FM	0.867	ATXN7L1	yes	no	G	A	chr4:5923681
Osteochondrosis	OC9	Icelandic	0.529	ATXN7L1	yes	no	G	A	chr4:5923681

NA: Variants not present in this population

Supplementary table 6. Breed specific allele frequencies for the known variants

Osteochondrosis	OC9	Clydesdale	0.342	ATXN7L1	yes	no	G	A	chr4:5923681
Osteochondrosis	OC9	Belgian	0.475	ATXN7L1	yes	no	G	A	chr4:5923681
Osteochondrosis	OC9	Shetland	0.436	ATXN7L1	yes	no	G	A	chr4:5923681
Osteochondrosis	OC9	Arabian	0.658	ATXN7L1	yes	no	G	A	chr4:5923681
Osteochondrosis	OC9	Warmblood	0.557	ATXN7L1	yes	no	G	A	chr4:5923681
Osteochondrosis	OC9	QH	0.514	ATXN7L1	yes	no	G	A	chr4:5923681
Osteochondrosis	OC9	STB	0.602	ATXN7L1	yes	no	G	A	chr4:5923681
Osteochondrosis	OC9	TB	0.586	ATXN7L1	yes	no	G	A	chr4:5923681
Osteochondrosis	OC9	Morgan	0.455	ATXN7L1	yes	no	G	A	chr4:5923681
Osteochondrosis	OC9	WP	0.425	ATXN7L1	yes	no	G	A	chr4:5923681
Ocular squamous cell carcinoma	occ_SCC	Icelandic	0.000	DDB2	yes	yes	C	T	chr12:11726667
Ocular squamous cell carcinoma	occ_SCC	TB	0.000	DDB2	yes	yes	C	T	chr12:11726667
Ocular squamous cell carcinoma	occ_SCC	FM	0.000	DDB2	yes	yes	C	T	chr12:11726667
Ocular squamous cell carcinoma	occ_SCC	QH	0.000	DDB2	yes	yes	C	T	chr12:11726667
Ocular squamous cell carcinoma	occ_SCC	Other	0.073	DDB2	yes	yes	C	T	chr12:11726667
Ocular squamous cell carcinoma	occ_SCC	Belgian	0.175	DDB2	yes	yes	C	T	chr12:11726667
Ocular squamous cell carcinoma	occ_SCC	Shetland	0.000	DDB2	yes	yes	C	T	chr12:11726667
Ocular squamous cell carcinoma	occ_SCC	Morgan	0.000	DDB2	yes	yes	C	T	chr12:11726667
Ocular squamous cell carcinoma	occ_SCC	Warmblood	0.000	DDB2	yes	yes	C	T	chr12:11726667
Ocular squamous cell carcinoma	occ_SCC	STB	0.000	DDB2	yes	yes	C	T	chr12:11726667
Ocular squamous cell carcinoma	occ_SCC	WP	0.050	DDB2	yes	yes	C	T	chr12:11726667
Ocular squamous cell carcinoma	occ_SCC	Clydesdale	0.000	DDB2	yes	yes	C	T	chr12:11726667
Ocular squamous cell carcinoma	occ_SCC	Arabian	0.000	DDB2	yes	yes	C	T	chr12:11726667
Prognathism	Pro1	QH	0.533	NA	yes	no	A	G	chr13:42032090
Prognathism	Pro1	Other	0.605	NA	yes	no	A	G	chr13:42032090
Prognathism	Pro1	FM	0.733	NA	yes	no	A	G	chr13:42032090
Prognathism	Pro1	Icelandic	1.000	NA	yes	no	A	G	chr13:42032090
Prognathism	Pro1	Belgian	0.725	NA	yes	no	A	G	chr13:42032090
Prognathism	Pro1	Clydesdale	0.895	NA	yes	no	A	G	chr13:42032090

NA: Variants not present in this population

Supplementary table 6. Breed specific allele frequencies for the known variants

Prognathism	Pro1	Shetland	0.718	NA	yes	no	A	G	chr13:42032090
Prognathism	Pro1	TB	0.230	NA	yes	no	A	G	chr13:42032090
Prognathism	Pro1	Warmblood	0.443	NA	yes	no	A	G	chr13:42032090
Prognathism	Pro1	Morgan	0.595	NA	yes	no	A	G	chr13:42032090
Prognathism	Pro1	STB	0.373	NA	yes	no	A	G	chr13:42032090
Prognathism	Pro1	WP	0.775	NA	yes	no	A	G	chr13:42032090
Prognathism	Pro1	Arabian	0.632	NA	yes	no	A	G	chr13:42032090
Prognathism	Pro2	QH	0.476	NA	yes	no	A	G	chr13:41985888
Prognathism	Pro2	Icelandic	1.000	NA	yes	no	A	G	chr13:41985888
Prognathism	Pro2	TB	0.171	NA	yes	no	A	G	chr13:41985888
Prognathism	Pro2	STB	0.280	NA	yes	no	A	G	chr13:41985888
Prognathism	Pro2	WP	0.800	NA	yes	no	A	G	chr13:41985888
Prognathism	Pro2	Other	0.477	NA	yes	no	A	G	chr13:41985888
Prognathism	Pro2	Morgan	0.432	NA	yes	no	A	G	chr13:41985888
Prognathism	Pro2	Shetland	0.536	NA	yes	no	A	G	chr13:41985888
Prognathism	Pro2	Belgian	0.675	NA	yes	no	A	G	chr13:41985888
Prognathism	Pro2	Warmblood	0.400	NA	yes	no	A	G	chr13:41985888
Prognathism	Pro2	Arabian	0.632	NA	yes	no	A	G	chr13:41985888
Prognathism	Pro2	FM	0.617	NA	yes	no	A	G	chr13:41985888
Prognathism	Pro2	Clydesdale	0.816	NA	yes	no	A	G	chr13:41985888
Polysaccharide storage myopathy/PSSM1/Exertic rhabdomyolysis	PSSM1	QH	0.061	GYS1	yes	yes	C	T	chr10:19203501
Polysaccharide storage myopathy/PSSM1/Exertic rhabdomyolysis	PSSM1	Shetland	0.000	GYS1	yes	yes	C	T	chr10:19203501
Polysaccharide storage myopathy/PSSM1/Exertic rhabdomyolysis	PSSM1	Icelandic	0.000	GYS1	yes	yes	C	T	chr10:19203501
Polysaccharide storage myopathy/PSSM1/Exertic rhabdomyolysis	PSSM1	Other	0.027	GYS1	yes	yes	C	T	chr10:19203501
Polysaccharide storage myopathy/PSSM1/Exertic rhabdomyolysis	PSSM1	TB	0.000	GYS1	yes	yes	C	T	chr10:19203501
Polysaccharide storage myopathy/PSSM1/Exertic rhabdomyolysis	PSSM1	Belgian	0.100	GYS1	yes	yes	C	T	chr10:19203501
Polysaccharide storage	PSSM1	FM	0.000	GYS1	yes	yes	C	T	chr10:19203501

NA: Variants not present in this population

Supplementary table 6. Breed specific allele frequencies for the known variants

myopathy/PSSM1/Exertic rhabdomyolysis									
Polysaccharide storage myopathy/PSSM1/Exertic rhabdomyolysis	PSSM1	STB	0.000	GYS1	yes	yes	C	T	chr10:19203501
Polysaccharide storage myopathy/PSSM1/Exertic rhabdomyolysis	PSSM1	Warmblood	0.000	GYS1	yes	yes	C	T	chr10:19203501
Polysaccharide storage myopathy/PSSM1/Exertic rhabdomyolysis	PSSM1	Arabian	0.000	GYS1	yes	yes	C	T	chr10:19203501
Polysaccharide storage myopathy/PSSM1/Exertic rhabdomyolysis	PSSM1	Morgan	0.000	GYS1	yes	yes	C	T	chr10:19203501
Polysaccharide storage myopathy/PSSM1/Exertic rhabdomyolysis	PSSM1	WP	0.000	GYS1	yes	yes	C	T	chr10:19203501
Polysaccharide storage myopathy/PSSM1/Exertic rhabdomyolysis	PSSM1	Clydesdale	0.000	GYS1	yes	yes	C	T	chr10:19203501
Polysaccharide storage myopathy, type 2	PSSM2_K1	WP	0.000	COL6A3	yes	no	C	G	chr6:23416882
Polysaccharide storage myopathy, type 2	PSSM2_K1	Icelandic	0.000	COL6A3	yes	no	C	G	chr6:23416882
Polysaccharide storage myopathy, type 2	PSSM2_K1	Warmblood	0.000	COL6A3	yes	no	C	G	chr6:23416882
Polysaccharide storage myopathy, type 2	PSSM2_K1	TB	0.000	COL6A3	yes	no	C	G	chr6:23416882
Polysaccharide storage myopathy, type 2	PSSM2_K1	Belgian	0.025	COL6A3	yes	no	C	G	chr6:23416882
Polysaccharide storage myopathy, type 2	PSSM2_K1	Other	0.018	COL6A3	yes	no	C	G	chr6:23416882
Polysaccharide storage myopathy, type 2	PSSM2_K1	QH	0.000	COL6A3	yes	no	C	G	chr6:23416882
Polysaccharide storage myopathy, type 2	PSSM2_K1	Shetland	0.027	COL6A3	yes	no	C	G	chr6:23416882
Polysaccharide storage myopathy, type 2	PSSM2_K1	Clydesdale	0.000	COL6A3	yes	no	C	G	chr6:23416882
Polysaccharide storage myopathy, type 2	PSSM2_K1	FM	0.000	COL6A3	yes	no	C	G	chr6:23416882
Polysaccharide storage myopathy, type 2	PSSM2_K1	STB	0.085	COL6A3	yes	no	C	G	chr6:23416882
Polysaccharide storage myopathy,	PSSM2_K1	Arabian	0.079	COL6A3	yes	no	C	G	chr6:23416882

NA: Variants not present in this population

Supplementary table 6. Breed specific allele frequencies for the known variants

type 2									
Polysaccharide storage myopathy, type 2	PSSM2_K1	Morgan	0.045	COL6A3	yes	no	C	G	chr6:23416882
Polysaccharide storage myopathy, type 2	PSSM2_P2	TB	0.158	MYOT	yes	no	A	G	chr14:37818823
Polysaccharide storage myopathy, type 2	PSSM2_P2	Warmblood	0.043	MYOT	yes	no	A	G	chr14:37818823
Polysaccharide storage myopathy, type 2	PSSM2_P2	Other	0.123	MYOT	yes	no	A	G	chr14:37818823
Polysaccharide storage myopathy, type 2	PSSM2_P2	QH	0.203	MYOT	yes	no	A	G	chr14:37818823
Polysaccharide storage myopathy, type 2	PSSM2_P2	Shetland	0.009	MYOT	yes	no	A	G	chr14:37818823
Polysaccharide storage myopathy, type 2	PSSM2_P2	WP	0.050	MYOT	yes	no	A	G	chr14:37818823
Polysaccharide storage myopathy, type 2	PSSM2_P2	Belgian	0.400	MYOT	yes	no	A	G	chr14:37818823
Polysaccharide storage myopathy, type 2	PSSM2_P2	STB	0.407	MYOT	yes	no	A	G	chr14:37818823
Polysaccharide storage myopathy, type 2	PSSM2_P2	Morgan	0.182	MYOT	yes	no	A	G	chr14:37818823
Polysaccharide storage myopathy, type 2	PSSM2_P2	Icelandic	0.000	MYOT	yes	no	A	G	chr14:37818823
Polysaccharide storage myopathy, type 2	PSSM2_P2	Arabian	0.145	MYOT	yes	no	A	G	chr14:37818823
Polysaccharide storage myopathy, type 2	PSSM2_P2	FM	0.350	MYOT	yes	no	A	G	chr14:37818823
Polysaccharide storage myopathy, type 2	PSSM2_P2	Clydesdale	0.184	MYOT	yes	no	A	G	chr14:37818823
Polysaccharide storage myopathy, type 2	PSSM2_P3_1	Warmblood	0.043	FLNC	yes	no	G	A	chr4:83837774
Polysaccharide storage myopathy, type 2	PSSM2_P3_1	QH	0.052	FLNC	yes	no	G	A	chr4:83837774
Polysaccharide storage myopathy, type 2	PSSM2_P3_1	Shetland	0.036	FLNC	yes	no	G	A	chr4:83837774
Polysaccharide storage myopathy, type 2	PSSM2_P3_1	STB	0.008	FLNC	yes	no	G	A	chr4:83837774
Polysaccharide storage myopathy, type 2	PSSM2_P3_1	FM	0.033	FLNC	yes	no	G	A	chr4:83837774

NA: Variants not present in this population

Supplementary table 6. Breed specific allele frequencies for the known variants

Polysaccharide storage myopathy, type 2	PSSM2_P3_1	Icelandic	0.059	FLNC	yes	no	G	A	chr4:83837774
Polysaccharide storage myopathy, type 2	PSSM2_P3_1	TB	0.039	FLNC	yes	no	G	A	chr4:83837774
Polysaccharide storage myopathy, type 2	PSSM2_P3_1	Belgian	0.000	FLNC	yes	no	G	A	chr4:83837774
Polysaccharide storage myopathy, type 2	PSSM2_P3_1	Arabian	0.000	FLNC	yes	no	G	A	chr4:83837774
Polysaccharide storage myopathy, type 2	PSSM2_P3_1	Morgan	0.045	FLNC	yes	no	G	A	chr4:83837774
Polysaccharide storage myopathy, type 2	PSSM2_P3_1	WP	0.025	FLNC	yes	no	G	A	chr4:83837774
Polysaccharide storage myopathy, type 2	PSSM2_P3_1	Clydesdale	0.053	FLNC	yes	no	G	A	chr4:83837774
Polysaccharide storage myopathy, type 2	PSSM2_P3_1	Other	0.059	FLNC	yes	no	G	A	chr4:83837774
Polysaccharide storage myopathy, type 2	PSSM2_P3_2	TB	0.086	FLNC	yes	no	G	A	chr4:83840299
Polysaccharide storage myopathy, type 2	PSSM2_P3_2	Warmblood	0.043	FLNC	yes	no	G	A	chr4:83840299
Polysaccharide storage myopathy, type 2	PSSM2_P3_2	Icelandic	0.059	FLNC	yes	no	G	A	chr4:83840299
Polysaccharide storage myopathy, type 2	PSSM2_P3_2	QH	0.061	FLNC	yes	no	G	A	chr4:83840299
Polysaccharide storage myopathy, type 2	PSSM2_P3_2	Shetland	0.036	FLNC	yes	no	G	A	chr4:83840299
Polysaccharide storage myopathy, type 2	PSSM2_P3_2	WP	0.025	FLNC	yes	no	G	A	chr4:83840299
Polysaccharide storage myopathy, type 2	PSSM2_P3_2	Clydesdale	0.026	FLNC	yes	no	G	A	chr4:83840299
Polysaccharide storage myopathy, type 2	PSSM2_P3_2	Other	0.064	FLNC	yes	no	G	A	chr4:83840299
Polysaccharide storage myopathy, type 2	PSSM2_P3_2	STB	0.008	FLNC	yes	no	G	A	chr4:83840299
Polysaccharide storage myopathy, type 2	PSSM2_P3_2	Morgan	0.068	FLNC	yes	no	G	A	chr4:83840299
Polysaccharide storage myopathy, type 2	PSSM2_P3_2	Belgian	0.000	FLNC	yes	no	G	A	chr4:83840299
Polysaccharide storage myopathy,	PSSM2_P3_2	FM	0.083	FLNC	yes	no	G	A	chr4:83840299

NA: Variants not present in this population

Supplementary table 6. Breed specific allele frequencies for the known variants

type 2									
Polysaccharide storage myopathy, type 2	PSSM2_P3_2	Arabian	0.000	FLNC	yes	no	G	A	chr4:83840299
Polysaccharide storage myopathy, type 2	PSSM2_P4	WP	0.075	MYOZ3	yes	no	G	A	chr14:26710261
Polysaccharide storage myopathy, type 2	PSSM2_P4	Icelandic	0.000	MYOZ3	yes	no	G	A	chr14:26710261
Polysaccharide storage myopathy, type 2	PSSM2_P4	TB	0.039	MYOZ3	yes	no	G	A	chr14:26710261
Polysaccharide storage myopathy, type 2	PSSM2_P4	Warmblood	0.057	MYOZ3	yes	no	G	A	chr14:26710261
Polysaccharide storage myopathy, type 2	PSSM2_P4	Shetland	0.018	MYOZ3	yes	no	G	A	chr14:26710261
Polysaccharide storage myopathy, type 2	PSSM2_P4	FM	0.250	MYOZ3	yes	no	G	A	chr14:26710261
Polysaccharide storage myopathy, type 2	PSSM2_P4	Other	0.009	MYOZ3	yes	no	G	A	chr14:26710261
Polysaccharide storage myopathy, type 2	PSSM2_P4	QH	0.061	MYOZ3	yes	no	G	A	chr14:26710261
Polysaccharide storage myopathy, type 2	PSSM2_P4	Belgian	0.000	MYOZ3	yes	no	G	A	chr14:26710261
Polysaccharide storage myopathy, type 2	PSSM2_P4	Arabian	0.079	MYOZ3	yes	no	G	A	chr14:26710261
Polysaccharide storage myopathy, type 2	PSSM2_P4	Morgan	0.091	MYOZ3	yes	no	G	A	chr14:26710261
Polysaccharide storage myopathy, type 2	PSSM2_P4	STB	0.008	MYOZ3	yes	no	G	A	chr14:26710261
Polysaccharide storage myopathy, type 2	PSSM2_P4	Clydesdale	0.000	MYOZ3	yes	no	G	A	chr14:26710261
Polysaccharide storage myopathy, type 2	PSSM2_P8	QH	0.042	PYROXD1	yes	no	G	C	chr6:48924749
Polysaccharide storage myopathy, type 2	PSSM2_P8	TB	0.066	PYROXD1	yes	no	G	C	chr6:48924749
Polysaccharide storage myopathy, type 2	PSSM2_P8	Other	0.045	PYROXD1	yes	no	G	C	chr6:48924749
Polysaccharide storage myopathy, type 2	PSSM2_P8	STB	0.000	PYROXD1	yes	no	G	C	chr6:48924749
Polysaccharide storage myopathy, type 2	PSSM2_P8	Shetland	0.000	PYROXD1	yes	no	G	C	chr6:48924749

NA: Variants not present in this population

Supplementary table 6. Breed specific allele frequencies for the known variants

Polysaccharide storage myopathy, type 2	PSSM2_P8	Warmblood	0.057	PYROXD1	yes	no	G	C	chr6:48924749
Polysaccharide storage myopathy, type 2	PSSM2_P8	Arabian	0.145	PYROXD1	yes	no	G	C	chr6:48924749
Polysaccharide storage myopathy, type 2	PSSM2_P8	Belgian	0.000	PYROXD1	yes	no	G	C	chr6:48924749
Polysaccharide storage myopathy, type 2	PSSM2_P8	FM	0.083	PYROXD1	yes	no	G	C	chr6:48924749
Polysaccharide storage myopathy, type 2	PSSM2_P8	WP	0.100	PYROXD1	yes	no	G	C	chr6:48924749
Polysaccharide storage myopathy, type 2	PSSM2_P8	Clydesdale	0.000	PYROXD1	yes	no	G	C	chr6:48924749
Polysaccharide storage myopathy, type 2	PSSM2_P8	Icelandic	0.029	PYROXD1	yes	no	G	C	chr6:48924749
Polysaccharide storage myopathy, type 2	PSSM2_P8	Morgan	0.091	PYROXD1	yes	no	G	C	chr6:48924749
Recurrent airway obstruction	RAO	TB	0.230	NA	yes	no	T	C	chr13:25400213
Recurrent airway obstruction	RAO	WP	0.375	NA	yes	no	T	C	chr13:25400213
Recurrent airway obstruction	RAO	QH	0.321	NA	yes	no	T	C	chr13:25400213
Recurrent airway obstruction	RAO	Warmblood	0.386	NA	yes	no	T	C	chr13:25400213
Recurrent airway obstruction	RAO	STB	0.339	NA	yes	no	T	C	chr13:25400213
Recurrent airway obstruction	RAO	Icelandic	0.382	NA	yes	no	T	C	chr13:25400213
Recurrent airway obstruction	RAO	Shetland	0.391	NA	yes	no	T	C	chr13:25400213
Recurrent airway obstruction	RAO	Arabian	0.329	NA	yes	no	T	C	chr13:25400213
Recurrent airway obstruction	RAO	Belgian	0.250	NA	yes	no	T	C	chr13:25400213
Recurrent airway obstruction	RAO	FM	0.233	NA	yes	no	T	C	chr13:25400213
Recurrent airway obstruction	RAO	Other	0.341	NA	yes	no	T	C	chr13:25400213
Recurrent airway obstruction	RAO	Clydesdale	0.289	NA	yes	no	T	C	chr13:25400213
Recurrent airway obstruction	RAO	Morgan	0.341	NA	yes	no	T	C	chr13:25400213
Racing distance	RD	Warmblood	0.157	MSTN	no	no	T	C	chr18:66608679
Racing distance	RD	Shetland	0.218	MSTN	no	no	T	C	chr18:66608679

NA: Variants not present in this population

Supplementary table 6. Breed specific allele frequencies for the known variants

Racing distance	RD	QH	0.792	MSTN	no	no	T	C	chr18:66608679
Racing distance	RD	Belgian	0.175	MSTN	no	no	T	C	chr18:66608679
Racing distance	RD	WP	0.175	MSTN	no	no	T	C	chr18:66608679
Racing distance	RD	TB	0.546	MSTN	no	no	T	C	chr18:66608679
Racing distance	RD	STB	0.017	MSTN	no	no	T	C	chr18:66608679
Racing distance	RD	Arabian	0.013	MSTN	no	no	T	C	chr18:66608679
Racing distance	RD	Icelandic	0.088	MSTN	no	no	T	C	chr18:66608679
Racing distance	RD	Other	0.168	MSTN	no	no	T	C	chr18:66608679
Racing distance	RD	Morgan	0.114	MSTN	no	no	T	C	chr18:66608679
Racing distance	RD	Clydesdale	0.158	MSTN	no	no	T	C	chr18:66608679
Racing distance	RD	FM	0.167	MSTN	no	no	T	C	chr18:66608679
Resistance susceptibility to arteritis virus	REAV1	QH	0.042	NA	yes	no	G	A	chr11:49912787
Resistance susceptibility to arteritis virus	REAV1	FM	0.250	NA	yes	no	G	A	chr11:49912787
Resistance susceptibility to arteritis virus	REAV1	Icelandic	0.000	NA	yes	no	G	A	chr11:49912787
Resistance susceptibility to arteritis virus	REAV1	TB	0.026	NA	yes	no	G	A	chr11:49912787
Resistance susceptibility to arteritis virus	REAV1	Warmblood	0.043	NA	yes	no	G	A	chr11:49912787
Resistance susceptibility to arteritis virus	REAV1	Arabian	0.132	NA	yes	no	G	A	chr11:49912787
Resistance susceptibility to arteritis virus	REAV1	Shetland	0.000	NA	yes	no	G	A	chr11:49912787
Resistance susceptibility to arteritis virus	REAV1	WP	0.000	NA	yes	no	G	A	chr11:49912787
Resistance susceptibility to arteritis virus	REAV1	Other	0.045	NA	yes	no	G	A	chr11:49912787
Resistance susceptibility to arteritis virus	REAV1	Belgian	0.000	NA	yes	no	G	A	chr11:49912787
Resistance susceptibility to arteritis virus	REAV1	Clydesdale	0.053	NA	yes	no	G	A	chr11:49912787
Resistance susceptibility to arteritis virus	REAV1	STB	0.008	NA	yes	no	G	A	chr11:49912787
Resistance susceptibility to arteritis virus	REAV1	Morgan	0.023	NA	yes	no	G	A	chr11:49912787

NA: Variants not present in this population

Supplementary table 6. Breed specific allele frequencies for the known variants

Resistance susceptibility to arteritis virus	REAV2	TB	0.026	NA	yes	no	G	C	chr11:49920362
Resistance susceptibility to arteritis virus	REAV2	Icelandic	0.206	NA	yes	no	G	C	chr11:49920362
Resistance susceptibility to arteritis virus	REAV2	Warmblood	0.086	NA	yes	no	G	C	chr11:49920362
Resistance susceptibility to arteritis virus	REAV2	Arabian	0.250	NA	yes	no	G	C	chr11:49920362
Resistance susceptibility to arteritis virus	REAV2	QH	0.108	NA	yes	no	G	C	chr11:49920362
Resistance susceptibility to arteritis virus	REAV2	Other	0.159	NA	yes	no	G	C	chr11:49920362
Resistance susceptibility to arteritis virus	REAV2	Belgian	0.150	NA	yes	no	G	C	chr11:49920362
Resistance susceptibility to arteritis virus	REAV2	Shetland	0.273	NA	yes	no	G	C	chr11:49920362
Resistance susceptibility to arteritis virus	REAV2	Clydesdale	0.474	NA	yes	no	G	C	chr11:49920362
Resistance susceptibility to arteritis virus	REAV2	STB	0.018	NA	yes	no	G	C	chr11:49920362
Resistance susceptibility to arteritis virus	REAV2	FM	0.583	NA	yes	no	G	C	chr11:49920362
Resistance susceptibility to arteritis virus	REAV2	WP	0.300	NA	yes	no	G	C	chr11:49920362
Resistance susceptibility to arteritis virus	REAV2	Morgan	0.048	NA	yes	no	G	C	chr11:49920362
Resistance susceptibility to arteritis virus	REAV3	TB	0.395	NA	yes	no	C	G	chr11:49924344
Resistance susceptibility to arteritis virus	REAV3	Warmblood	0.786	NA	yes	no	C	G	chr11:49924344
Resistance susceptibility to arteritis virus	REAV3	Belgian	0.925	NA	yes	no	C	G	chr11:49924344
Resistance susceptibility to arteritis virus	REAV3	Other	0.650	NA	yes	no	C	G	chr11:49924344
Resistance susceptibility to arteritis virus	REAV3	QH	0.835	NA	yes	no	C	G	chr11:49924344
Resistance susceptibility to arteritis virus	REAV3	Shetland	0.673	NA	yes	no	C	G	chr11:49924344
Resistance susceptibility to	REAV3	Icelandic	1.000	NA	yes	no	C	G	chr11:49924344

NA: Variants not present in this population

Supplementary table 6. Breed specific allele frequencies for the known variants

arteritis virus									
Resistance susceptibility to arteritis virus	REAV3	Arabian	0.803	NA	yes	no	C	G	chr11:49924344
Resistance susceptibility to arteritis virus	REAV3	Clydesdale	0.842	NA	yes	no	C	G	chr11:49924344
Resistance susceptibility to arteritis virus	REAV3	FM	0.933	NA	yes	no	C	G	chr11:49924344
Resistance susceptibility to arteritis virus	REAV3	WP	0.850	NA	yes	no	C	G	chr11:49924344
Resistance susceptibility to arteritis virus	REAV3	STB	0.966	NA	yes	no	C	G	chr11:49924344
Resistance susceptibility to arteritis virus	REAV3	Morgan	0.841	NA	yes	no	C	G	chr11:49924344
Resistance susceptibility to arteritis virus	REAV4	Warmblood	0.814	NA	yes	no	A	G	chr11:49929783
Resistance susceptibility to arteritis virus	REAV4	Arabian	0.829	NA	yes	no	A	G	chr11:49929783
Resistance susceptibility to arteritis virus	REAV4	TB	0.368	NA	yes	no	A	G	chr11:49929783
Resistance susceptibility to arteritis virus	REAV4	Icelandic	1.000	NA	yes	no	A	G	chr11:49929783
Resistance susceptibility to arteritis virus	REAV4	Other	0.777	NA	yes	no	A	G	chr11:49929783
Resistance susceptibility to arteritis virus	REAV4	QH	0.835	NA	yes	no	A	G	chr11:49929783
Resistance susceptibility to arteritis virus	REAV4	FM	0.967	NA	yes	no	A	G	chr11:49929783
Resistance susceptibility to arteritis virus	REAV4	WP	0.950	NA	yes	no	A	G	chr11:49929783
Resistance susceptibility to arteritis virus	REAV4	Belgian	0.925	NA	yes	no	A	G	chr11:49929783
Resistance susceptibility to arteritis virus	REAV4	Shetland	0.709	NA	yes	no	A	G	chr11:49929783
Resistance susceptibility to arteritis virus	REAV4	Morgan	0.841	NA	yes	no	A	G	chr11:49929783
Resistance susceptibility to arteritis virus	REAV4	STB	0.966	NA	yes	no	A	G	chr11:49929783
Resistance susceptibility to arteritis virus	REAV4	Clydesdale	1.000	NA	yes	no	A	G	chr11:49929783

NA: Variants not present in this population

Supplementary table 6. Breed specific allele frequencies for the known variants

Resistance susceptibility to arteritis virus	REAV5	Icelandic	1.000	NA	yes	no	C	A	chr11:49953686
Resistance susceptibility to arteritis virus	REAV5	Clydesdale	1.000	NA	yes	no	C	A	chr11:49953686
Resistance susceptibility to arteritis virus	REAV5	Belgian	0.875	NA	yes	no	C	A	chr11:49953686
Resistance susceptibility to arteritis virus	REAV5	Warmblood	0.814	NA	yes	no	C	A	chr11:49953686
Resistance susceptibility to arteritis virus	REAV5	QH	0.816	NA	yes	no	C	A	chr11:49953686
Resistance susceptibility to arteritis virus	REAV5	Shetland	0.727	NA	yes	no	C	A	chr11:49953686
Resistance susceptibility to arteritis virus	REAV5	TB	0.382	NA	yes	no	C	A	chr11:49953686
Resistance susceptibility to arteritis virus	REAV5	STB	0.966	NA	yes	no	C	A	chr11:49953686
Resistance susceptibility to arteritis virus	REAV5	FM	0.917	NA	yes	no	C	A	chr11:49953686
Resistance susceptibility to arteritis virus	REAV5	Morgan	0.773	NA	yes	no	C	A	chr11:49953686
Resistance susceptibility to arteritis virus	REAV5	Other	0.786	NA	yes	no	C	A	chr11:49953686
Resistance susceptibility to arteritis virus	REAV5	Arabian	0.855	NA	yes	no	C	A	chr11:49953686
Resistance susceptibility to arteritis virus	REAV5	WP	0.950	NA	yes	no	C	A	chr11:49953686
Resistance susceptibility to arteritis virus	REAV6	TB	0.368	NA	yes	no	A	G	chr11:49967200
Resistance susceptibility to arteritis virus	REAV6	QH	0.792	NA	yes	no	A	G	chr11:49967200
Resistance susceptibility to arteritis virus	REAV6	FM	0.933	NA	yes	no	A	G	chr11:49967200
Resistance susceptibility to arteritis virus	REAV6	Icelandic	0.824	NA	yes	no	A	G	chr11:49967200
Resistance susceptibility to arteritis virus	REAV6	Other	0.495	NA	yes	no	A	G	chr11:49967200
Resistance susceptibility to arteritis virus	REAV6	STB	0.839	NA	yes	no	A	G	chr11:49967200
Resistance susceptibility to	REAV6	Shetland	0.655	NA	yes	no	A	G	chr11:49967200

NA: Variants not present in this population

Supplementary table 6. Breed specific allele frequencies for the known variants

arteritis virus									
Resistance susceptibility to arteritis virus	REAV6	WP	0.950	NA	yes	no	A	G	chr11:49967200
Resistance susceptibility to arteritis virus	REAV6	Warmblood	0.657	NA	yes	no	A	G	chr11:49967200
Resistance susceptibility to arteritis virus	REAV6	Clydesdale	0.842	NA	yes	no	A	G	chr11:49967200
Resistance susceptibility to arteritis virus	REAV6	Belgian	0.775	NA	yes	no	A	G	chr11:49967200
Resistance susceptibility to arteritis virus	REAV6	Morgan	0.523	NA	yes	no	A	G	chr11:49967200
Resistance susceptibility to arteritis virus	REAV6	Arabian	0.776	NA	yes	no	A	G	chr11:49967200
Resistance susceptibility to arteritis virus	REAV7	Other	0.718	NA	yes	no	T	G	chr11:49977514
Resistance susceptibility to arteritis virus	REAV7	Morgan	0.659	NA	yes	no	T	G	chr11:49977514
Resistance susceptibility to arteritis virus	REAV7	TB	0.382	NA	yes	no	T	G	chr11:49977514
Resistance susceptibility to arteritis virus	REAV7	Icelandic	1.000	NA	yes	no	T	G	chr11:49977514
Resistance susceptibility to arteritis virus	REAV7	Shetland	0.673	NA	yes	no	T	G	chr11:49977514
Resistance susceptibility to arteritis virus	REAV7	Belgian	0.925	NA	yes	no	T	G	chr11:49977514
Resistance susceptibility to arteritis virus	REAV7	STB	0.924	NA	yes	no	T	G	chr11:49977514
Resistance susceptibility to arteritis virus	REAV7	QH	0.835	NA	yes	no	T	G	chr11:49977514
Resistance susceptibility to arteritis virus	REAV7	WP	0.900	NA	yes	no	T	G	chr11:49977514
Resistance susceptibility to arteritis virus	REAV7	Clydesdale	0.947	NA	yes	no	T	G	chr11:49977514
Resistance susceptibility to arteritis virus	REAV7	FM	0.967	NA	yes	no	T	G	chr11:49977514
Resistance susceptibility to arteritis virus	REAV7	Warmblood	0.814	NA	yes	no	T	G	chr11:49977514
Resistance susceptibility to arteritis virus	REAV7	Arabian	0.855	NA	yes	no	T	G	chr11:49977514

NA: Variants not present in this population

Supplementary table 6. Breed specific allele frequencies for the known variants

Resistance susceptibility to arteritis virus	REAV8	Shetland	0.082	NA	yes	no	C	T	chr11:49983675
Resistance susceptibility to arteritis virus	REAV8	TB	0.164	NA	yes	no	C	T	chr11:49983675
Resistance susceptibility to arteritis virus	REAV8	Other	0.395	NA	yes	no	C	T	chr11:49983675
Resistance susceptibility to arteritis virus	REAV8	QH	0.425	NA	yes	no	C	T	chr11:49983675
Resistance susceptibility to arteritis virus	REAV8	Warmblood	0.486	NA	yes	no	C	T	chr11:49983675
Resistance susceptibility to arteritis virus	REAV8	Belgian	0.075	NA	yes	no	C	T	chr11:49983675
Resistance susceptibility to arteritis virus	REAV8	Icelandic	0.500	NA	yes	no	C	T	chr11:49983675
Resistance susceptibility to arteritis virus	REAV8	STB	0.932	NA	yes	no	C	T	chr11:49983675
Resistance susceptibility to arteritis virus	REAV8	Arabian	0.632	NA	yes	no	C	T	chr11:49983675
Resistance susceptibility to arteritis virus	REAV8	Clydesdale	0.237	NA	yes	no	C	T	chr11:49983675
Resistance susceptibility to arteritis virus	REAV8	FM	0.483	NA	yes	no	C	T	chr11:49983675
Resistance susceptibility to arteritis virus	REAV8	WP	0.525	NA	yes	no	C	T	chr11:49983675
Resistance susceptibility to arteritis virus	REAV8	Morgan	0.705	NA	yes	no	C	T	chr11:49983675
Laryngeal paralysis, generic	RLN	QH	0.241	NA	yes	no	C	A	chr15:31725377
Laryngeal paralysis, generic	RLN	WP	0.075	NA	yes	no	C	A	chr15:31725377
Laryngeal paralysis, generic	RLN	Shetland	0.036	NA	yes	no	C	A	chr15:31725377
Laryngeal paralysis, generic	RLN	TB	0.033	NA	yes	no	C	A	chr15:31725377
Laryngeal paralysis, generic	RLN	Icelandic	0.206	NA	yes	no	C	A	chr15:31725377
Laryngeal paralysis, generic	RLN	Arabian	0.263	NA	yes	no	C	A	chr15:31725377
Laryngeal paralysis, generic	RLN	STB	0.025	NA	yes	no	C	A	chr15:31725377
Laryngeal paralysis, generic	RLN	FM	0.117	NA	yes	no	C	A	chr15:31725377

NA: Variants not present in this population

Supplementary table 6. Breed specific allele frequencies for the known variants

Laryngeal paralysis, generic	RLN	Morgan	0.068	NA	yes	no	C	A	chr15:31725377
Laryngeal paralysis, generic	RLN	Other	0.236	NA	yes	no	C	A	chr15:31725377
Laryngeal paralysis, generic	RLN	Warmblood	0.114	NA	yes	no	C	A	chr15:31725377
Laryngeal paralysis, generic	RLN	Clydesdale	0.079	NA	yes	no	C	A	chr15:31725377
Laryngeal paralysis, generic	RLN	Belgian	0.175	NA	yes	no	C	A	chr15:31725377
Laryngeal paralysis, generic	RLN_height1	Icelandic	0.000	NA	no	no	C	T	chr1:56992379
Laryngeal paralysis, generic	RLN_height1	Arabian	0.092	NA	no	no	C	T	chr1:56992379
Laryngeal paralysis, generic	RLN_height1	Belgian	0.600	NA	no	no	C	T	chr1:56992379
Laryngeal paralysis, generic	RLN_height1	Morgan	0.045	NA	no	no	C	T	chr1:56992379
Laryngeal paralysis, generic	RLN_height1	Other	0.159	NA	no	no	C	T	chr1:56992379
Laryngeal paralysis, generic	RLN_height1	Clydesdale	0.500	NA	no	no	C	T	chr1:56992379
Laryngeal paralysis, generic	RLN_height1	FM	0.150	NA	no	no	C	T	chr1:56992379
Laryngeal paralysis, generic	RLN_height1	STB	0.059	NA	no	no	C	T	chr1:56992379
Laryngeal paralysis, generic	RLN_height1	Shetland	0.018	NA	no	no	C	T	chr1:56992379
Laryngeal paralysis, generic	RLN_height1	QH	0.071	NA	no	no	C	T	chr1:56992379
Laryngeal paralysis, generic	RLN_height1	WP	0.050	NA	no	no	C	T	chr1:56992379
Laryngeal paralysis, generic	RLN_height1	TB	0.112	NA	no	no	C	T	chr1:56992379
Laryngeal paralysis, generic	RLN_height1	Warmblood	0.114	NA	no	no	C	T	chr1:56992379
Laryngeal paralysis, generic	RLN_height2	QH	0.085	NA	no	no	G	T	chr1:57037172
Laryngeal paralysis, generic	RLN_height2	Icelandic	0.000	NA	no	no	G	T	chr1:57037172
Laryngeal paralysis, generic	RLN_height2	Shetland	0.082	NA	no	no	G	T	chr1:57037172
Laryngeal paralysis, generic	RLN_height2	Belgian	0.625	NA	no	no	G	T	chr1:57037172
Laryngeal paralysis, generic	RLN_height2	WP	0.025	NA	no	no	G	T	chr1:57037172
Laryngeal paralysis, generic	RLN_height2	TB	0.132	NA	no	no	G	T	chr1:57037172
Laryngeal paralysis,	RLN_height2	Other	0.155	NA	no	no	G	T	chr1:57037172

NA: Variants not present in this population

Supplementary table 6. Breed specific allele frequencies for the known variants

generic									
Laryngeal paralysis, generic	RLN_height2	FM	0.183	NA	no	no	G	T	chr1:57037172
Laryngeal paralysis, generic	RLN_height2	STB	0.034	NA	no	no	G	T	chr1:57037172
Laryngeal paralysis, generic	RLN_height2	Arabian	0.105	NA	no	no	G	T	chr1:57037172
Laryngeal paralysis, generic	RLN_height2	Warmblood	0.114	NA	no	no	G	T	chr1:57037172
Laryngeal paralysis, generic	RLN_height2	Clydesdale	0.553	NA	no	no	G	T	chr1:57037172
Laryngeal paralysis, generic	RLN_height2	Morgan	0.295	NA	no	no	G	T	chr1:57037172
Severe combined immunodeficiency disease, autosomal	SCID	TB	0.000	PRKDC	yes	yes	ATCATC	A	chr9:36395748
Severe combined immunodeficiency disease, autosomal	SCID	FM	0.000	PRKDC	yes	yes	ATCATC	A	chr9:36395748
Severe combined immunodeficiency disease, autosomal	SCID	Belgian	0.000	PRKDC	yes	yes	ATCATC	A	chr9:36395748
Severe combined immunodeficiency disease, autosomal	SCID	Other	0.000	PRKDC	yes	yes	ATCATC	A	chr9:36395748
Severe combined immunodeficiency disease, autosomal	SCID	WP	0.000	PRKDC	yes	yes	ATCATC	A	chr9:36395748
Severe combined immunodeficiency disease, autosomal	SCID	Clydesdale	0.000	PRKDC	yes	yes	ATCATC	A	chr9:36395748
Severe combined immunodeficiency disease, autosomal	SCID	Morgan	0.000	PRKDC	yes	yes	ATCATC	A	chr9:36395748
Severe combined immunodeficiency disease, autosomal	SCID	Icelandic	0.000	PRKDC	yes	yes	ATCATC	A	chr9:36395748
Severe combined immunodeficiency disease, autosomal	SCID	Warmblood	0.000	PRKDC	yes	yes	ATCATC	A	chr9:36395748
Severe combined immunodeficiency disease, autosomal	SCID	Arabian	0.026	PRKDC	yes	yes	ATCATC	A	chr9:36395748
Severe combined immunodeficiency disease, autosomal	SCID	QH	0.000	PRKDC	yes	yes	ATCATC	A	chr9:36395748
Severe combined immunodeficiency disease, autosomal	SCID	Shetland	0.000	PRKDC	yes	yes	ATCATC	A	chr9:36395748
Severe combined immunodeficiency disease, autosomal	SCID	STB	0.000	PRKDC	yes	yes	ATCATC	A	chr9:36395748
Small stature	Small_H	Clydesdale	0.000	HMGA2	yes	no	G	A	chr6:82553845
Small stature	Small_H	Icelandic	0.000	HMGA2	yes	no	G	A	chr6:82553845

NA: Variants not present in this population

Supplementary table 6. Breed specific allele frequencies for the known variants

Small stature	Small_H	Other	0.045	HMGA2	yes	no	G	A	chr6:82553845
Small stature	Small_H	Belgian	0.000	HMGA2	yes	no	G	A	chr6:82553845
Small stature	Small_H	Morgan	0.000	HMGA2	yes	no	G	A	chr6:82553845
Small stature	Small_H	Shetland	0.236	HMGA2	yes	no	G	A	chr6:82553845
Small stature	Small_H	STB	0.000	HMGA2	yes	no	G	A	chr6:82553845
Small stature	Small_H	WP	0.400	HMGA2	yes	no	G	A	chr6:82553845
Small stature	Small_H	TB	0.000	HMGA2	yes	no	G	A	chr6:82553845
Small stature	Small_H	FM	0.000	HMGA2	yes	no	G	A	chr6:82553845
Small stature	Small_H	QH	0.000	HMGA2	yes	no	G	A	chr6:82553845
Small stature	Small_H	Arabian	0.000	HMGA2	yes	no	G	A	chr6:82553845
Small stature	Small_H	Warmblood	0.000	HMGA2	yes	no	G	A	chr6:82553845
Small stature	Small_O	QH	0.000	OSTN	yes	no	G	A	chr19:30992750
Small stature	Small_O	Arabian	0.000	OSTN	yes	no	G	A	chr19:30992750
Small stature	Small_O	Belgian	0.000	OSTN	yes	no	G	A	chr19:30992750
Small stature	Small_O	STB	0.000	OSTN	yes	no	G	A	chr19:30992750
Small stature	Small_O	Warmblood	0.000	OSTN	yes	no	G	A	chr19:30992750
Small stature	Small_O	Clydesdale	0.000	OSTN	yes	no	G	A	chr19:30992750
Small stature	Small_O	FM	0.000	OSTN	yes	no	G	A	chr19:30992750
Small stature	Small_O	TB	0.000	OSTN	yes	no	G	A	chr19:30992750
Small stature	Small_O	Shetland	0.509	OSTN	yes	no	G	A	chr19:30992750
Small stature	Small_O	WP	0.000	OSTN	yes	no	G	A	chr19:30992750
Small stature	Small_O	Morgan	0.000	OSTN	yes	no	G	A	chr19:30992750
Small stature	Small_O	Icelandic	0.000	OSTN	yes	no	G	A	chr19:30992750
Small stature	Small_O	Other	0.032	OSTN	yes	no	G	A	chr19:30992750
Coat colour, albinism, oculocutaneous type VI	TE1	NA	NA	SLC24A5	yes	yes	A	T	chr1:143145865
Thrombasthenia	Thromb1	Shetland	0.000	ITGA2B	yes	yes	G	C	chr11:19245752
Thrombasthenia	Thromb1	Belgian	0.000	ITGA2B	yes	yes	G	C	chr11:19245752
Thrombasthenia	Thromb1	Warmblood	0.000	ITGA2B	yes	yes	G	C	chr11:19245752
Thrombasthenia	Thromb1	Arabian	0.000	ITGA2B	yes	yes	G	C	chr11:19245752
Thrombasthenia	Thromb1	Clydesdale	0.000	ITGA2B	yes	yes	G	C	chr11:19245752
Thrombasthenia	Thromb1	FM	0.000	ITGA2B	yes	yes	G	C	chr11:19245752
Thrombasthenia	Thromb1	WP	0.000	ITGA2B	yes	yes	G	C	chr11:19245752
Thrombasthenia	Thromb1	TB	0.000	ITGA2B	yes	yes	G	C	chr11:19245752

NA: Variants not present in this population

Supplementary table 6. Breed specific allele frequencies for the known variants

Thrombasthenia	Thromb1	Other	0.000	ITGA2B	yes	yes	G	C	chr11:19245752
Thrombasthenia	Thromb1	QH	0.005	ITGA2B	yes	yes	G	C	chr11:19245752
Thrombasthenia	Thromb1	Icelandic	0.000	ITGA2B	yes	yes	G	C	chr11:19245752
Thrombasthenia	Thromb1	STB	0.000	ITGA2B	yes	yes	G	C	chr11:19245752
Thrombasthenia	Thromb1	Morgan	0.000	ITGA2B	yes	yes	G	C	chr11:19245752
Thrombasthenia	Thromb2	NA	NA	ITGA2B	yes	yes	CAGGTGAG	*	chr11:19247983
Vitiligo	Vit1	Shetland	0.073	NUBPL	no	no	A	G	chr1:170926834
Vitiligo	Vit1	TB	0.000	NUBPL	no	no	A	G	chr1:170926834
Vitiligo	Vit1	Arabian	0.000	NUBPL	no	no	A	G	chr1:170926834
Vitiligo	Vit1	Belgian	0.075	NUBPL	no	no	A	G	chr1:170926834
Vitiligo	Vit1	WP	0.025	NUBPL	no	no	A	G	chr1:170926834
Vitiligo	Vit1	STB	0.000	NUBPL	no	no	A	G	chr1:170926834
Vitiligo	Vit1	Morgan	0.000	NUBPL	no	no	A	G	chr1:170926834
Vitiligo	Vit1	Clydesdale	0.000	NUBPL	no	no	A	G	chr1:170926834
Vitiligo	Vit1	FM	0.317	NUBPL	no	no	A	G	chr1:170926834
Vitiligo	Vit1	Other	0.045	NUBPL	no	no	A	G	chr1:170926834
Vitiligo	Vit1	QH	0.000	NUBPL	no	no	A	G	chr1:170926834
Vitiligo	Vit1	Icelandic	0.147	NUBPL	no	no	A	G	chr1:170926834
Vitiligo	Vit1	Warmblood	0.000	NUBPL	no	no	A	G	chr1:170926834
Vitiligo	Vit2	WP	0.000	PHF11	no	no	T	C	chr17:21505125
Vitiligo	Vit2	STB	0.000	PHF11	no	no	T	C	chr17:21505125
Vitiligo	Vit2	TB	0.000	PHF11	no	no	T	C	chr17:21505125
Vitiligo	Vit2	Icelandic	0.206	PHF11	no	no	T	C	chr17:21505125
Vitiligo	Vit2	Morgan	0.068	PHF11	no	no	T	C	chr17:21505125
Vitiligo	Vit2	QH	0.009	PHF11	no	no	T	C	chr17:21505125
Vitiligo	Vit2	FM	0.000	PHF11	no	no	T	C	chr17:21505125
Vitiligo	Vit2	Other	0.018	PHF11	no	no	T	C	chr17:21505125
Vitiligo	Vit2	Clydesdale	0.000	PHF11	no	no	T	C	chr17:21505125
Vitiligo	Vit2	Shetland	0.173	PHF11	no	no	T	C	chr17:21505125
Vitiligo	Vit2	Belgian	0.025	PHF11	no	no	T	C	chr17:21505125
Vitiligo	Vit2	Warmblood	0.029	PHF11	no	no	T	C	chr17:21505125
Vitiligo	Vit2	Arabian	0.000	PHF11	no	no	T	C	chr17:21505125
Vitiligo	Vit3	QH	0.042	PTP4A1	no	no	A	G	chr20:57714448
Vitiligo	Vit3	Icelandic	0.118	PTP4A1	no	no	A	G	chr20:57714448

NA: Variants not present in this population

Supplementary table 6. Breed specific allele frequencies for the known variants

Vitiligo	Vit3	Arabian	0.171	PTP4A1	no	no	A	G	chr20:57714448
Vitiligo	Vit3	Clydesdale	0.053	PTP4A1	no	no	A	G	chr20:57714448
Vitiligo	Vit3	FM	0.050	PTP4A1	no	no	A	G	chr20:57714448
Vitiligo	Vit3	TB	0.000	PTP4A1	no	no	A	G	chr20:57714448
Vitiligo	Vit3	Other	0.100	PTP4A1	no	no	A	G	chr20:57714448
Vitiligo	Vit3	STB	0.008	PTP4A1	no	no	A	G	chr20:57714448
Vitiligo	Vit3	Shetland	0.000	PTP4A1	no	no	A	G	chr20:57714448
Vitiligo	Vit3	Morgan	0.000	PTP4A1	no	no	A	G	chr20:57714448
Vitiligo	Vit3	Warmblood	0.071	PTP4A1	no	no	A	G	chr20:57714448
Vitiligo	Vit3	WP	0.150	PTP4A1	no	no	A	G	chr20:57714448
Vitiligo	Vit3	Belgian	0.050	PTP4A1	no	no	A	G	chr20:57714448

NA: Variants not present in this population
